# An Immunity-Triggering Effector from the Barley Smut Fungus *Ustilago hordei* Resides in an Ustilaginaceae-Specific Cluster Bearing Signs of Transposable Element-Assisted Evolution

**DOI:** 10.1371/journal.ppat.1004223

**Published:** 2014-07-03

**Authors:** Shawkat Ali, John D. Laurie, Rob Linning, José Antonio Cervantes-Chávez, Denis Gaudet, Guus Bakkeren

**Affiliations:** 1 Agriculture & Agri-Food Canada, Pacific Agri-Food Research Centre, Summerland, British Columbia, Canada; 2 Department of Botany, University of British Columbia, Vancouver, British Columbia, Canada; 3 Agriculture & Agri-Food Canada, Lethbridge Research Centre, Lethbridge, Alberta, Canada; Purdue University, United States of America

## Abstract

The basidiomycete smut fungus *Ustilago hordei* was previously shown to comprise isolates that are avirulent on various barley host cultivars. Through genetic crosses we had revealed that a dominant avirulence locus *UhAvr1* which triggers immunity in barley cultivar Hannchen harboring resistance gene *Ruh1*, resided within an 80-kb region. DNA sequence analysis of this genetically delimited region uncovered the presence of 7 candidate secreted effector proteins. Sequence comparison of their coding sequences among virulent and avirulent parental and field isolates could not distinguish *UhAvr1* candidates. Systematic deletion and complementation analyses revealed that *UhAvr1* is *UHOR_10022* which codes for a small effector protein of 171 amino acids with a predicted 19 amino acid signal peptide. Virulence in the parental isolate is caused by the insertion of a fragment of 5.5 kb with similarity to a common *U. hordei* transposable element (TE), interrupting the promoter of *UhAvr1* and thereby changing expression and hence recognition of UhAVR1p. This rearrangement is likely caused by activities of TEs and variation is seen among isolates. Using GFP-chimeric constructs we show that *UhAvr1* is induced only in mated dikaryotic hyphae upon sensing and infecting barley coleoptile cells. When infecting Hannchen, UhAVR1p causes local callose deposition and the production of reactive oxygen species and necrosis indicative of the immune response. *UhAvr1* does not contribute significantly to overall virulence. *UhAvr1* is located in a cluster of ten effectors with several paralogs and over 50% of TEs. This cluster is syntenous with clusters in closely-related *U. maydis* and *Sporisorium reilianum*. In these corn-infecting species, these clusters harbor however more and further diversified homologous effector families but very few TEs. This increased variability may have resulted from past selection pressure by resistance genes since *U. maydis* is not known to trigger immunity in its corn host.

## Author Summary

Upon host infection, plant pathogens secrete suites of virulence effectors to suppress defense responses and support their own development. In certain cases, hosts evolve resistance genes that recognize such effectors or their actions to initiate defense responses. By deleting candidate genes, we identified the immune-triggering effector *UhAvr1* from *Ustilago hordei*, a barley-infecting basidiomycete smut fungus. We show that this effector is expressed only when hyphae sense and infect barley coleoptile epidermal cells. Its presence in the fungus causes a necrotic reaction immediately upon penetration resulting in complete immunity in barley cultivars having resistance gene *Ruh1*. We show that fungal isolates that have mutated to change the expression of this non-crucial protein are avoiding recognition by the host, hence overcoming restriction by its immune response. In virulent isolates, transposable elements, known as genome modifiers, have separated the *UhAvr1* coding region from its transcription signals. *UhAvr1* is located in a larger cluster of ten effectors and is similar to clusters with more and further diversified effectors in the related maize pathogens *U. maydis* and *Sporisorium reilianum*. This study should lead us to discovering a mechanism by which this major cereal crop protects itself against this pathogen.

## Introduction

Pathogenic microbes secrete hundreds of compounds and proteins into their host as part of the infection strategy. This arsenal of virulence factors, often small proteins with a predicted signal peptide (SP), effectors or candidate secreted effector proteins (CSEPs), functions to facilitate entry, to subdue defense responses that may be triggered through their recognition by the hosts' surveillance system, to divert nutrients and to ensure proliferation [1]–[4]. Plants use a variety of defense mechanisms to avoid pathogen invasion and subsequent disease, including physical barriers, preformed antimicrobial compounds, but also activation of defenses. In particular, defenses can be induced by the recognition of highly conserved pathogen molecules (Pathogen- Associated Molecular Patterns or PAMPs) resulting in a broad-based PAMP-triggered immunity (PTI). Certain pathogen effectors, whether secreted into the host apoplast or vessels and taken up, or delivered directly into cells to perform their function, are inadvertently recognized directly or through their action by a highly sophisticated system of which resistance (*R*) genes are a part, to elicit effector-triggered immunity or ETI [5]–[8]. Induced immunity includes cell wall strengthening, the generation of an environment toxic to the pathogen, encasement of the pathogen and localized programmed cell death (PCD) to arrest pathogen development [9]. Although the latter affects development of biotrophic pathogens, necrotrophic pathogens and hemibiotrophs at later stages of infection might have evolved to take advantage of triggering PCD [10], [11]. Earlier studies in several pathosystems demonstrated the presence of genetically dominant avirulence (*Avr*) genes in pathogens, the products of which have been shown more recently to often be effectors, interacting genetically with also often dominant host *R* genes. This concept was developed by Harold Flor using the flax-rust *Melampsora lini* pathosystem [12] and simultaneously by his contemporary, Arend Oort who studied the wheat-loose smut *Ustilago tritici* pathosystem but because of WWII could only publish his results in 1944 in Dutch [13].

In pathogen populations, there is strong selection to avoid recognition resulting in rapidly evolving effectors and, in response, evolving host *R* genes or effector targets [14], [15]. This natural arms race is accelerated in agricultural settings where invading pathogens necessitate the introduction of resistant host cultivars from breeding programs, thereby triggering boom-bust cycles. In many cases, *Avr* genes are present in genomic regions displaying high flexibility, such as telomeres [16], heterochromatic locations [17], [18], or are surrounded by transposable elements (TEs) [17], [19]–[22] which can facilitate effector gene mutation.

Basidiomycete smut fungi are important pathogens that cause disease world-wide and are of economic importance on many *Poaceae* [23]–[25]. *Ustilago maydis*, the maize smut fungus, has become the paradigm for molecular genetic studies on biotrophic basidiomycete plant pathogens [26], [27]. The barley covered smut fungus, *U. hordei*, is closely related but differs in important aspects: in *U. hordei*, race- and strain-specific virulence compatibility interactions exist whereas no dominant avirulence functions that genetically interact with dominant host resistance genes on a gene-for-gene basis have been identified in *U. maydis*. Moreover, *U. hordei* can infect only at the seed germination stage to develop quiescently in the meristematic region until sporulation occurs mainly in the seed heads [28] (**Figure S1**), a characteristic shared with many smut fungi, such as the maize-infecting *Sporisorium reilianum* [29]. In contrast, *U. maydis* can infect any above-ground part of the maize plant at any plant age, resulting in the proliferation and sporulation of the fungus in tumors it incites. In addition, *U. hordei* has differently organized mating-type loci affecting its biology [30], [31] and it has a larger genome due to a much higher content of repeats and TEs [32].

Six *Avr* genes have been genetically identified in *U. hordei* which in different combinations constitute 14 different reported races; six corresponding resistance genes have been proposed in barley [33]–[36]. *UhAvr1* determines avirulence towards barley cultivar Hannchen which has matching resistance gene *Ruh1* which we recently mapped to the short arm of barley chromosome 7H [37]. The *UhAvr1* locus was located to an approximately 80-kb region contained on Bacterial Artificial Chromosome (BAC) clone 3-A2, using a marker-based approach in a mapping population of 54 progeny segregating for avirulence towards Hannchen, resulting from a cross between parental lines Uh362 (*MAT-2 Uhavr1*) and Uh364 (*MAT-1 UhAvr1*) [38]. We show here that this locus spans a cluster of predicted secreted protein genes on chromosome 18 and we identify through targeted deletions and complementation the gene with the *UhAvr1* avirulence function, coding for a predicted secreted effector. The locus is syntenic to cluster 19A in both *U. maydis* and *S. reilianum* that also contains small proteins predicted to be secreted [26], [39], but has evolved differently. *UhAvr1* is located in a transposon- and repeat-rich region and transposon activity seems responsible for breaking the avirulence towards Hannchen. In virulent isolates, it appears that insertion of transposable element sequences in the promoter of *UhAvr1* has changed its expression.

## Results

### Sequence comparison of CSEP genes among *Avr1* and *vir1* phenotypes

**Figure 1:**
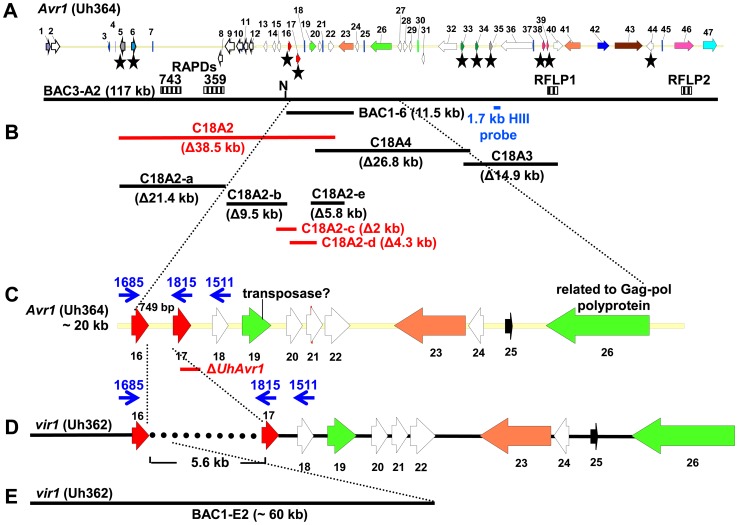
Map of *UhAvr1* and *Uhavr1* locus regions. **A.** *UhAvr1* locus region in *U. hordei* strain Uh364 with arrows representing all predicted ORFs with their direction of transcription (see **Table S1** for gene calls and similarity to *U. maydis* cluster 19A homologs; [26]). Asterisks indicate the predicted secreted proteins encoding genes and N denotes the unique NotI site. BAC clone BAC3-A2 containing 117 kb of this locus is shown by a solid black bar. Complementing Xba1-fragment in subclone BAC1-6 is indicated underneath. Indicated by vertically striped boxes are RAPD markers 743 and 359 which identified and delimited the original region by revealing two and one recombinants in the population, respectively (∼2 and ∼1 cM distance; [38]) and, at the other genetic boundary, RFLP2 which revealed three different recombinants in the population or approximately 6 cM in distance; the 1.7 kb HindIII probe and RFLP marker 1 revealed no recombinants. **B.** Lines denote the regions in kb deleted in the respective Uh364 mutants; in red are the deletion mutants resulting in a virulent phenotype, whereas the others remained avirulent on Hannchen. **C.** Enlarged region containing gene 17 (*UHOR_10022* as *UhAvr1*) and ten other ORFs. The red line below indicates the C-terminal deletion in gene 17 in mutant Uh1289 resulting in a virulent phenotype. The blue arrows and numbers refer to specific primers. **D.** Comparison to the syntenous region in the virulent parent Uh362 revealed the replacement of 634 bp by an insertion of a 5.5-kb, shown by the dotted line, part of which matches TE-related sequences. **E.** An overlapping BAC clone, BAC1E-2, containing the syntenous region and extending 1.2 kb past the end of gene 16 in the virulent parent Uh362, was used for sequencing.

BAC clone BAC3A-2, genetically harbouring *U. hordei* avirulence gene *UhAvr1*, was sequenced by GPS transposon insertion resulting in an assembled sequence of 117 kb. The presence of repeats and TE sequences made assembly challenging. On this BAC insert we identified 47 ORFs (**Figure 1A** **, Table S1**). Hybridization to DNA blots of separated chromosomes located this region to a 667-kb chromosome ([38], **Figure S2**), designated as *U. hordei* Chr 18, a homolog of *U. maydis* Chr19 in our recent comparative genome study [32]. In light of publications in which a number of avirulence gene products were CSEPs, we hypothesized that UhAVR1p could also be a secreted effector. On the sequenced BAC clone, ten predicted CSEPs were identified but only seven were likely candidates: gene 5 and gene 6 were located outside the genetic interval identified previously and gene 44 was very close to RFLP marker 2 which revealed three recombinants (**Figure 1A** **, Table S1** [38]).

A change from avirulence to virulence would likely be caused by a mutation in the candidate gene such as a point mutation, leading to an amino acid change or a protein truncation, or a gene deletion or a change in transcription. To identify the *UhAvr1* gene, we first checked the presence of the CSEP genes in the virulent parent Uh362. A PCR-amplification product for all ten genes was obtained from genomic DNA indicating their presence in the genome of the virulent parent. DNA sequence analysis of the seven candidate *UhAvr1* genes did reveal point mutations in three of the alleles in Uh362 (**Table 1**). Since CSEP 35 displayed an amino acid difference that could have changed its charge between the virulent and avirulent form, gene 35 was deleted in parental avirulent strain Uh364. However, when crossed with virulent parent Uh362, it did not result in virulence on cultivar Hannchen harboring *Ruh1*. We therefore expanded the sequence comparisons to include a collection of field isolates from different parts of the world, four avirulent and six virulent on Hannchen (**Table S2**). Three of the six remaining likely candidate *UhAvr1* genes were identical when comparing allelic sequences from virulent or avirulent isolates but in the other three, a few mutations were found (**Table 1** **, Figure S3**). Unfortunately, none of the revealed mutations could be correlated with the *Avr1* or *avr1* phenotypes. This indicated that there were other changes outside of the sequences we investigated, that were responsible for the change in phenotype, or that the avirulence function did not reside in the selected effector candidates.

**Table 1:** Mutations found in 7 CSEPs among isolates.

CSEP gene^1^	mutations in Uh362 (*avr1*)^2^	mutation in other isolates^3^
**16**	−21, +165 (G-to-A, changed V^55^ to I^55^)	+165 (G-to-A) in Uh1273 (*Avr1*), Uh1283 (*Avr1*), Uh362 (*avr1*)
		+518 (A-to-G) in Uh1283 (*Avr1*)
**17**	no	+506,507 (TT-to-GA changed I^169^ to R^169^) in Uh813 (*Avr1*) and Uh1273 (*Avr1*) but not in other *Avr1* isolates
**33**	no	no
**34**	+520 (T-to-C in STOP, adds 41 aa)	+520 (T-to-C in STOP, adds 41 aa) in Uh813 (*Avr1*)
35	+233 (G-to-A, R^78^ into a H^78^)	ND
**38**	no	no
**39**	no	no

1 genes in bold are *UhAvr1* candidates.

2 bp position is indicated with the amino acid (aa) change in parentheses (**Figure S3**); ND, not determined.

3 world-wid isolates (**Table S2**).

### Identification of *UhAvr1* by deletion analysis

**Figure 2:**
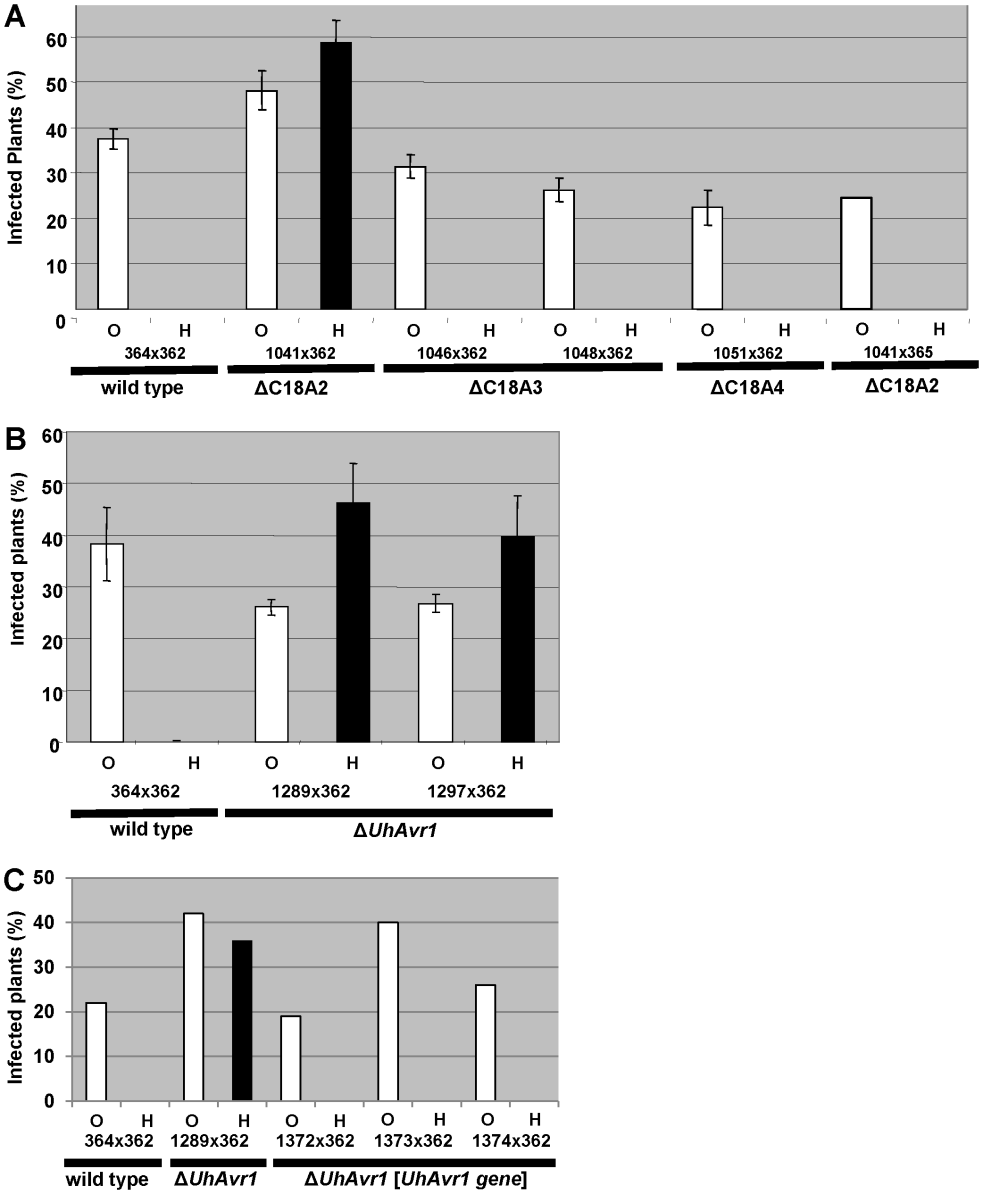
Pathogenicity test of deletion and complementation mutants. **A.** Fragment C18A2 contains the functional *UhAvr1* as this deletion mutant (strain Uh1041, **Figure 1B** **, Table S2**) crossed with parental virulent strain Uh362 was virulent towards Hannchen (black bar). All other deletion mutants and wild type are avirulent towards Hannchen because of the presence of a functional *UhAvr1*, whereas all are virulent towards Odessa. Y-axis, percent infected plants out of the total number of inoculated plants. Average of three independent inoculation experiments with standard deviation is shown as error bars. Uh1041×avirulent Uh365, a Uh364 sibling of opposite mating type (**Table S2**), is a control cross. **B.** Deletion of the 3′-part of gene 17 (strains Uh1289 and Uh1297, **Table S2**) proves this gene represents *UhAvr1*. **C.** Random integration of gene 17 sequences including 5′- and 3′-flanking regions, complementing single Δ*UhAvr1* deletion strain Uh1289, is sufficient to fully prevent disease on Hannchen (transformants Uh1372, Uh1373 and Uh1374, **Tables S2 and S3**).

Since no likely candidate for *UhAvr1* was found, a systematic deletion analysis of the 80-kb region delimited by the markers (**Figure 1**) was conducted using a marker-exchange method. In a first round, the region was divided into three sections, ranging from 15 to 38 kb in size, taking into account the location of the various predicted CSEP genes in the region (**Figure 1B** **, Figure S4**). No phenotypic differences or abnormal growth were observed for any of the haploid basidiospore deletion mutants and proper mating with compatible haploid basidiospores, such as virulent parental strain Uh362 necessary for pathogenicity tests, occurred. Mated strains were tested for pathogenicity by inoculating them on differential barley cultivars Hannchen (*Ruh1*) and Odessa (*ruh1*). Deletion of fragment C18A2 from avirulent parental strain Uh364 yielded strain Uh1041 (Uh364 Δ*18A2*) (**Table S2**) and resulted in disease on Hannchen after mating with compatible virulent wild-type strain Uh362 (**Figure 2A**), clearly indicating that the 38.5 kb fragment C18A2 contained avirulence gene *UhAvr1*. When Uh1041 was crossed with avirulent strain Uh365, a sibling to parental strain Uh364 but of opposite mating type, the resulting dikaryon caused disease on Odessa but not on Hannchen (**Figure 2A**), indicating complementation with the avirulence function and showing that no other functions in the recognition of the dominant *UhAvr1* allele had been inadvertently compromised in the mutant.

Fragment 18A2 harboring two CSEPs as the likely *UhAvr1* allele, was further divided to make five additional deletion mutants (sub-sections C18A2-a to C18A2-e; **Figure 1B**). To generate the deletion constructs, primers were designed in such a way that the two CSEPs would be deleted in two different deletion constructs. Sixty-four PCR-positive deletion mutants were obtained for the five deletion constructs, which were further verified by DNA blot analysis (**Figure S5**). Nine deletion mutants were selected, two from each, except for C18A2-b for which only one expected deletion mutant was obtained. Among these, the two mutants for C18A2-c and the two for C18A2-d were virulent towards both barley cultivars Odessa and Hannchen in pathogenicity tests after mating with Uh362 (**Figure S5G**). The overlapping fragments C18A2-c and C18A2-d shared only gene 17 encoding a CSEP (**Figure 1B**) that was a strong candidate for UhAVR1p. To confirm this, another deletion mutant was produced in which the 3′ 319 bp of the ORF of this gene in parental strain Uh364 was deleted (**Figure S6A and B**). Two independent deletion mutants, Uh1289 and Uh1297, had this small deletion which resulted in virulence towards both Hannchen and Odessa when crossed with Uh362, producing disease in 40–50% of the plants (**Figure 2B**), and confirmed that gene 17 (*UHOR_10022*, GenBank CCF49778.1) is necessary for *UhAvr1* avirulence function.

### Complementation of the virulent Uh364 deletion mutants

A 11.5-kb XbaI fragment cloned in a modified BAC vector (pUSBAC5, converted for use in *Ustilago* species by introducing a specific hygromycin B resistance cassette [38]) yielded construct BAC1-6 which contained two predicted CSEPs: gene 16 and 17 (**Figure 1B**). This clone partially overlaps with fragment C18A2. C18A2 deletion mutant Uh1041, virulent towards Hannchen, was complemented with BAC1-6 and two stable transformants (Uh1205 and Uh1207; **Table S2**) were inoculated on barley cultivars Odessa and Hannchen after mating with compatible virulent strain Uh362. No abnormal growth or defect in mating behavior had been observed in these haploid complemented strains. After mating with Uh362, the complemented strains caused the same level of disease on Odessa as the wild-type cross and the deletion mutant, infecting from 30–40% of the plants. On Hannchen however, the level of disease was severely reduced compared to the deletion mutant and only ∼2.5% of the plants showed infected seed heads (**Figure S7**). Incomplete restoration of avirulence could have resulted from the integration of an incomplete fragment at random locations in the genome, affecting transcription. Similar results were obtained for *Fusarium oxysporum* f. sp *lycopersici* mutant strains complemented with the *Six1* avirulence gene that did not restore complete avirulence towards tomato lines that contained the resistance gene *I-3* [40].This suggested that BAC1-6 contained the functional *UhAvr1* gene. To exclude any possible effects from other genes contained on the BAC clone or on the deleted C18A2 fragment, single *UhAvr1* deletion mutant strain Uh1289 was complemented with complete wild-type gene 17 sequences, including 659 bp and 630 bp from the upstream and downstream regions, respectively. Three independent transformants, strains Uh1372, Uh1373 and Uh1374 (**Table S2**), completely prevented disease on Hannchen (**Table S3**), confirming that gene 17 is sufficient to restore avirulence and hence codes for *UhAvr1* (**Figure 2C**).

*UhAvr1* codes for a predicted full-length protein of 170 amino acids with a calculated Mw of 21 kDa. SignalP 4.1 predicts a 19 amino acids SP resulting in a processed mature protein of 18.9 kDa. Mature UhAVR1p has predicted coil, helix and extended beta structures but could not be modeled on any currently existing crystal structures (**Figure S8**) and no clear similarities could be found to known proteins or other domains.

### Loss of avirulence is due to TE-activity upstream of the *UhAvr1* ORF

**Figure 3:**
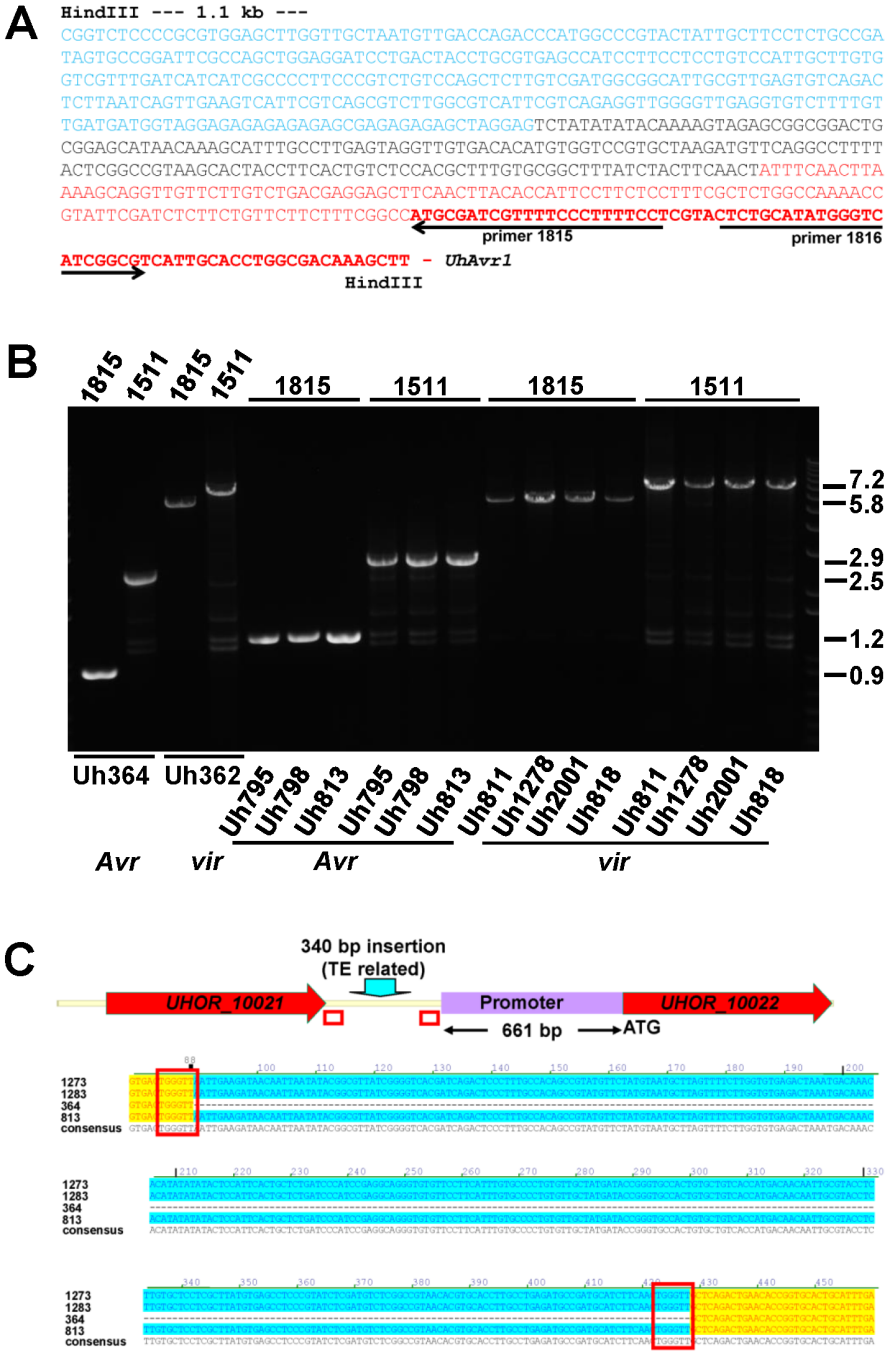
Analysis of DNA sequences surrounding *UhAvr1* in virulent and avirulent isolates. **A.** Inverse PCR of HindIII-digested, diluted and religated Uh362 gDNA, using outward-facing primers 1815 and 1816, generated a product of 1.8 kb. DNA sequencing from primer 1815 revealed the intact *UhAvr1* ORF (red bold face) plus 115 bp of the 5′ upstream region (red) before the sequence started to diverge, indicating the insertion site. This unknown sequence consisted of 166 bases revealing no match to any *U. hordei* genome sequence or any sequences in public databases (black). Adjacent, the sequence (in blue) matched known TE sequences in the *U. hordei* genome sequence, in particular related to retrotransposon protein (E-value = 2.9e-36). Highly similar sequences were found on the same chromosome 18 in Uh364 (*Avr1*) at positions 18.6 and 27.2 kb from the *UhAvr1* gene. **B.** EtBr-stained agarose gel showing length polymorphisms of PCR products among various *U. hordei* isolates avirulent (*Avr*) or virulent (*vir*) towards Hannchen, as indicated at the bottom (**Table S2**). On top, numbers refer to the primers used in combination with the “anchor” primer 1685 (**Figure 1C and D**). Sizes on the right are in Kb. **C.** Sequence comparison of the intergenic region between gene 16 and *UhAvr1* suggests TE activity. The numbers indicate the base pair position within the 749 bp-intergenic region in the avirulent parent Uh364 and were compared to three different field isolates also avirulent on Hannchen (see **Table S2**). Note the insertion of 340 bp, matching TE sequences, upstream of the *UhAvr1* promoter in the three other isolates (highlighted in blue) leaving a 661-bp promoter sequence apparently sufficient for avirulence function. This insert is flanked by two 6-bp direct repeats (TGGGTT, boxed), one of which is found in Uh364, possibly representing a “footprint”, i.e., a target site duplication, suggestive of (past) TE activity. See **Figure S3C** for details, also revealing other sequence variation in the remainder of the intergenic region among the field isolates.

From a BAC library constructed from genomic DNA from virulent strain Uh362, a BAC clone, BAC1-E2, was identified using gene 1 sequences as a probe (**Figure 1E**). We had found that all predicted CSEP ORFs could be amplified by PCR from genomic DNA of strain Uh362, but amplification of *UhAvr1*, gene 21 and subsequent genes further to the right could not be achieved from BAC1-E2; a probe representing gene 18 did not hybridize to a DNA blot from this BAC clone suggesting its insert did not cover this region. Sequencing and assembly of this BAC clone proved challenging due to the presence of many repeats and TEs, as it had been for BAC3-A2. Comparative analysis revealed indeed that one end of the BAC clone insert extended only 1187 bp past the stop codon of gene 16. Synteny between the virulent and avirulent parents however, was apparent only up to 115 bp upstream of the start codon of *UhAvr1* (**Figure 1C–E** **, Figure S3B**). WUBLAST analysis found a 400-bp sequence after this break point, matching to two retrotransposon proteins (*UHOR_14086* and *UHOR_14170*) in the *U. hordei* genome. To reveal the sequence upstream of the *UhAvr1* ORF in the genome of Uh362, an inverse PCR was conducted with *UhAvr1* ORF-specific primers on HindIII-digested and self-ligated genomic DNA. Sequence analysis confirmed the presence of intact *UhAvr1* sequences including 115 bases upstream of its start codon. Further 5′, the sequence diverged revealing no other Uh364-derived sequences and after 166 bases matched sequences with high similarity to the common long-terminal repeat sequence LTR5 from *U. hordei* Tuh3, a copia-type retrotransposon also found in the mating-type region (**Figure 3A**, [32], [41]). A PCR product of 5.8 kb however, was amplified from gDNA from the virulent parent when using primer 1685 at the 3′-end of gene 16 and primer 1815 located at the 5′-end of *UhAvr1* (**Figure 1D** **,** **Figure 3B** **, Table S4**). Several other primer combinations confirmed that an insertion of approximately 5.5 kb had occurred and that the genes to the right were preserved with respect to the organization in Uh364 (**Figure 3B** and not shown). Consistently, hybridization of three probes representing gene 16 (left of the breakpoint), *UhAvr1* and gene 23 (approximately 12 kb right of the breakpoint) to separated chromosomes of parental strains Uh364 and Uh362, clearly revealed that all genes were located to the same Chr18 (**Figure S2**). Combined with sequence information from this insertion, the data suggested that in strain Uh362, TE activity had inserted a seemingly intact TE consisting of gag-pol sequences flanked by LTRs in the intergenic region between gene 16 and *UhAvr1*. We speculated that this event separated the *UhAvr1* ORF from promoter elements thereby likely changing its expression and hence recognition in Hannchen, making this isolate virulent on this cultivar.

*UhAvr1* is located in a region of the genome that is, with the mating-type region, among the richest in repeats and TEs, approaching 50% compared to an overall genome content of 8–10% [32]. This elevated presence/retention of TEs and repeats at this effector locus suggests that this region represents a more dynamic part of the genome, enabling evolutionary changes as proposed for other pathosystems [17], [19]–[22]. If this effector region is under selection pressure, e.g., to modify the expression of *UhAvr1* to avoid triggering immune responses, it is conceivable that TE activity and insertions might have played a role. Since TE activity rates cannot easily be investigated, we assessed possible variation at the *UhAvr1* locus in a number of field isolates. In Uh364, avirulent on Hannchen, 749 bp separate gene 16 and *UhAvr1* from each other in the genome and amplification with primers 1685 and 1815 or 1511 (**Figure 1C** **, Table S4**) yields PCR products of 898 and 2462 bp respectively (**Figure 3B**). In five available avirulent field isolates, larger products were obtained for both primer combinations (results for three isolates are shown in **Figure 3B**) and upon sequencing revealed identical 340-bp insertions in the intergenic region in isolates Uh813, Uh1273 and Uh1283 (**Figure 3C**). This insertion also matched TE sequences in the *U. hordei* genome and was flanked by 6-bp repeats (TGGGTT), possibly a footprint of TE activity. This particular insertion, though apparently not affecting avirulence in these isolates, was not found in the virulent parent Uh362. The region was also analyzed from eight *U. hordei* field isolates virulent on Hannchen (Uh805, Uh811, Uh815, Uh818, Uh820, Uh822, Uh1278 and Uh2001-246; **Table S2**). Primer combination 1685 and 1815 or 1511produced PCR products of approximately 5.8 and 7.2 kb respectively, similar to the products obtained from Uh362 (**Figure 3C**). However, upon sequencing, variation was revealed among the TE sequences in the different virulent strains. One predominant mutation found in four virulent strains (Uh362, Uh805, Uh815, and Uh820) was a 10-bp insertion of a repeat (GAGAGAGAGC) that was however absent from three other virulent strains (Uh811, Uh818, and Uh822; **Figure S3C**). The 340-bp insertion discovered in three of the avirulent field isolates was not found in these eight virulent field isolates. Overall, the variation found in sequences surrounding *UhAvr1* in field isolates both avirulent and virulent on Hannchen, and the similarity of those sequences to various *U. hordei*-specific repeats and TEs, suggest various transposition events have occurred in different isolates resulting in a variety of combinations upon which selection could act.

### *UhAvr1* causes programmed cell death and is expressed in hyphae during plant infection

**Figure 4:**
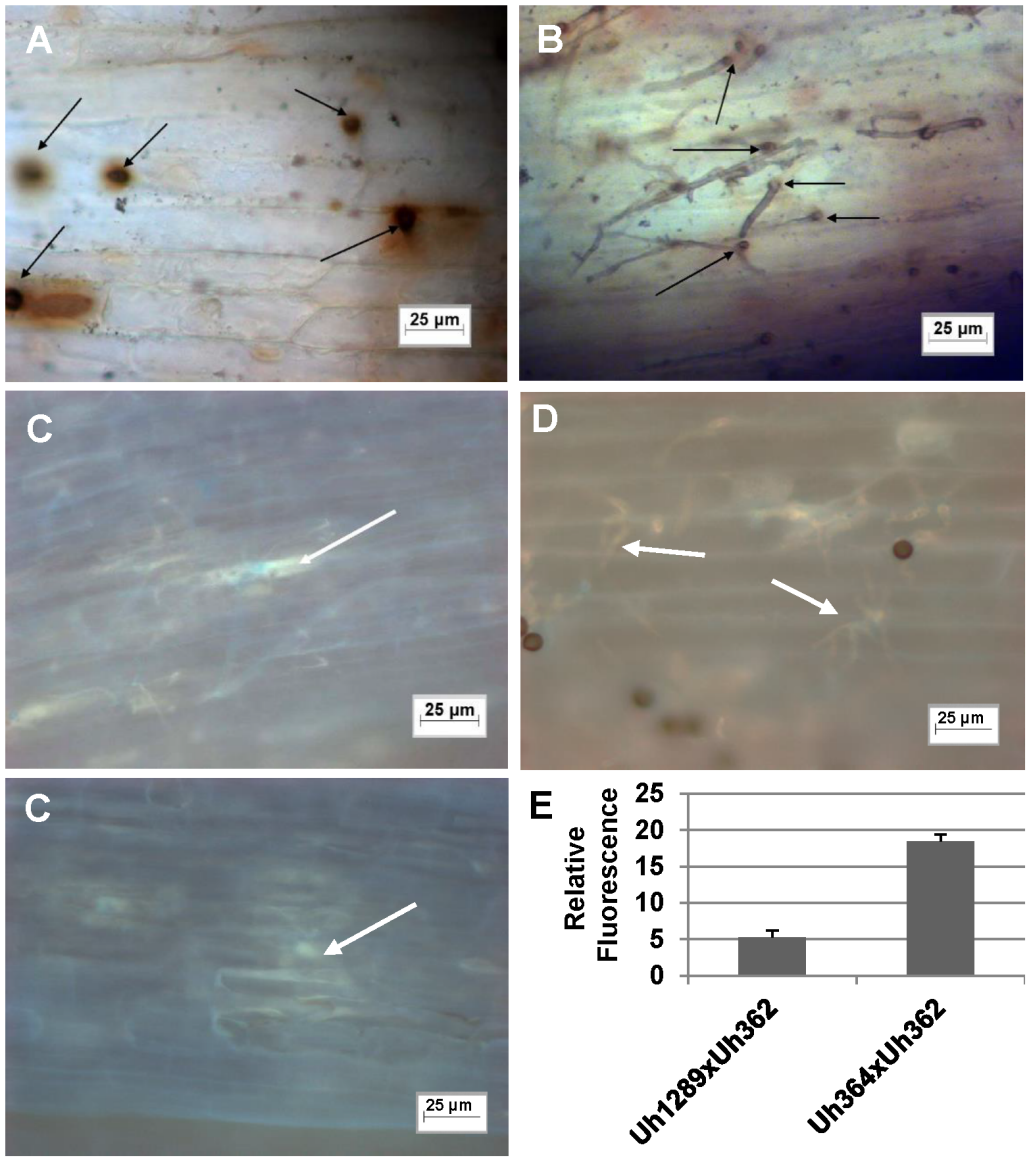
Light microscopic analysis of compatible and incompatible infection types. **A.** and **C.** Infection by teliospores from wild-type cross Uh364 (*MAT-1, UhAvr1*)×Uh362 (*MAT-2, Uhavr1*) on Hannchen (*Ruh1*) coleoptiles leads to restricted growth of infection hyphae after mating and penetration. Hypersensitive response-associated reactions include an extensive oxidative burst (DAB staining in **A** associated with infection sites (arrows) at 72 hrs after inoculation) and accumulation of callose and associated fluorescence around the restricted hyphae (arrows in **C** after 120 hrs; two panels showing different representative sites). **B** and **D.** Inoculation of *U. hordei* teliospores from cross Uh1289 (Uh364, Δ*UhAvr1*)×Uh362 (*MAT-2, Uhavr1*) showing compatibility on cv. Hannchen, leading to invasive growth where the oxidative burst is not extensively triggered at infection sites (arrows, DAB staining in **B** at 72 hrs after inoculation), and less callose and associated fluorescence around the spreading hyphae is observed at 120 hrs (arrows in **D**). **E.** Quantitation of fluorescence, representing callose, averaged over several penetration sites.

Previous electronmicroscopy work revealed necrosis in cells immediately surrounding penetration sites early upon infection during an incompatible interaction on Hannchen [42]. We performed a microscopic analysis of the natural infection process by teliospores, previously produced on universal susceptible cultivar Odessa. Infection of coleoptiles of cultivar Hannchen by teliospores from crossed wild-type progenitor strains Uh364 (*MAT-1 UhAvr1*)×Uh362 (*MAT-2 Uhavr1*) caused extensive production of reactive oxygen species as visualized by DAB staining suggesting cell death could have been initiated (**Figure 4A**), and extensive callose deposition seemingly restricting pathogen development (**Figure 4C and E**). In stark contrast, teliospores produced from cross Uh1289 (Uh364 *MAT-1*, Δ*UhAvr1*)×Uh362 (*MAT-2 Uhavr1*) caused a natural infection of coleoptile epidermal cells of cultivar Hannchen, showing hyphal development, very little oxidative damage (**Figure 4B**) and limited, diffuse callose depositions (**Figure 4D and E**), illustrating a compatible interaction.

**Figure 5:**
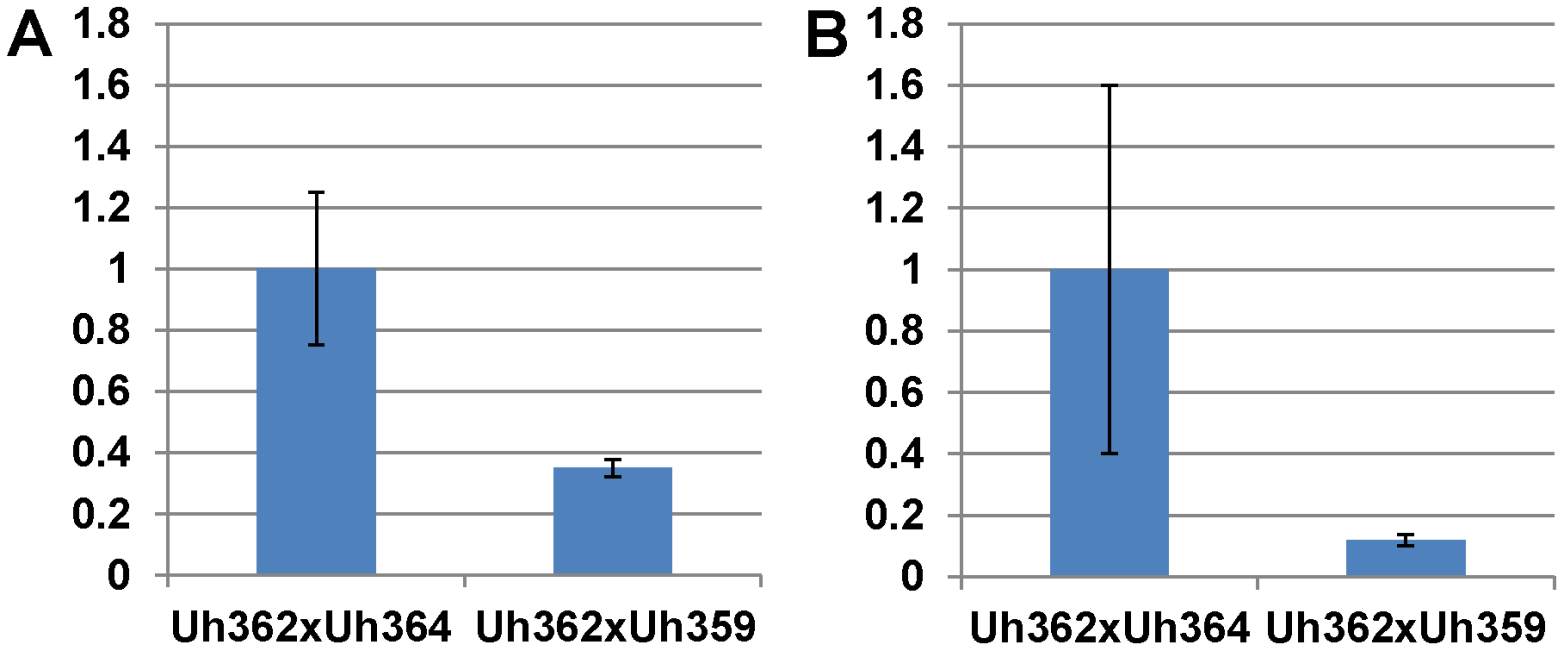
Expression of *UhAvr1* early in infection of coleoptiles. Quantitative Reverse Transcriptase PCR analysis measuring *UhAvr1* gene transcript levels in total RNA isolated from barley cv. Hannchen (*Ruh1*) coleoptiles 48 hrs after inoculation with mated cell cultures of crosses as indicated: Uh362 (*MAT-2, Uhavr1*)×Uh364 (*MAT-1, UhAvr1*) versus Uh362×Uh359 (*MAT-1, Uhavr1*). **A.** Measurable *UhAvr1* product was found when Uh364 was present but amplification from cross Uh362×Uh359 appeared not until cycle 38, at the limit of detection (P = 0.06, student t-test). **B.** cDNA from the *UhAvr1* target and the *U. hordei eIF-2B* reference genes was pre-amplified for 10 cycles before quantitative RT-PCR with nested primers resulting in significant variation.

Expression analysis of *UhAvr1* by quantitative RT-PCR during infection proved challenging. No expression could be detected in haploid avirulent or virulent cells grown in liquid media, or during mating interactions on plates (data not shown). Weak and variable expression was observed in mated cells and teliospores applied to barley coleoptiles but always only when avirulent strain Uh364 was employed (**Figure 5A**); linear pre-amplification of cDNA to increase signal strength [43] corroborated these results but introduced variation (**Figure 5B**). This suggested that the expression of *UhAvr1* might be induced only upon direct contact with, or actual infection of coleoptile epidermal cells. The low amount of transcript is likely due to the very few contact and penetration sites present resulting in a very small proportion of responding cells in the biological material (the inoculated coleoptiles) from which the RNA was isolated; this resulted in variable qRT-PCR results. From the combined data it was evident that in the virulent parental strain Uh362 (or its virulent sibling Uh359) the level of *UhAvr1* mRNA bordered on the limit of detection, but was at most only 10% of the level seen in Uh364 after pre-amplification (**Figure 5B**).

**Figure 6:**
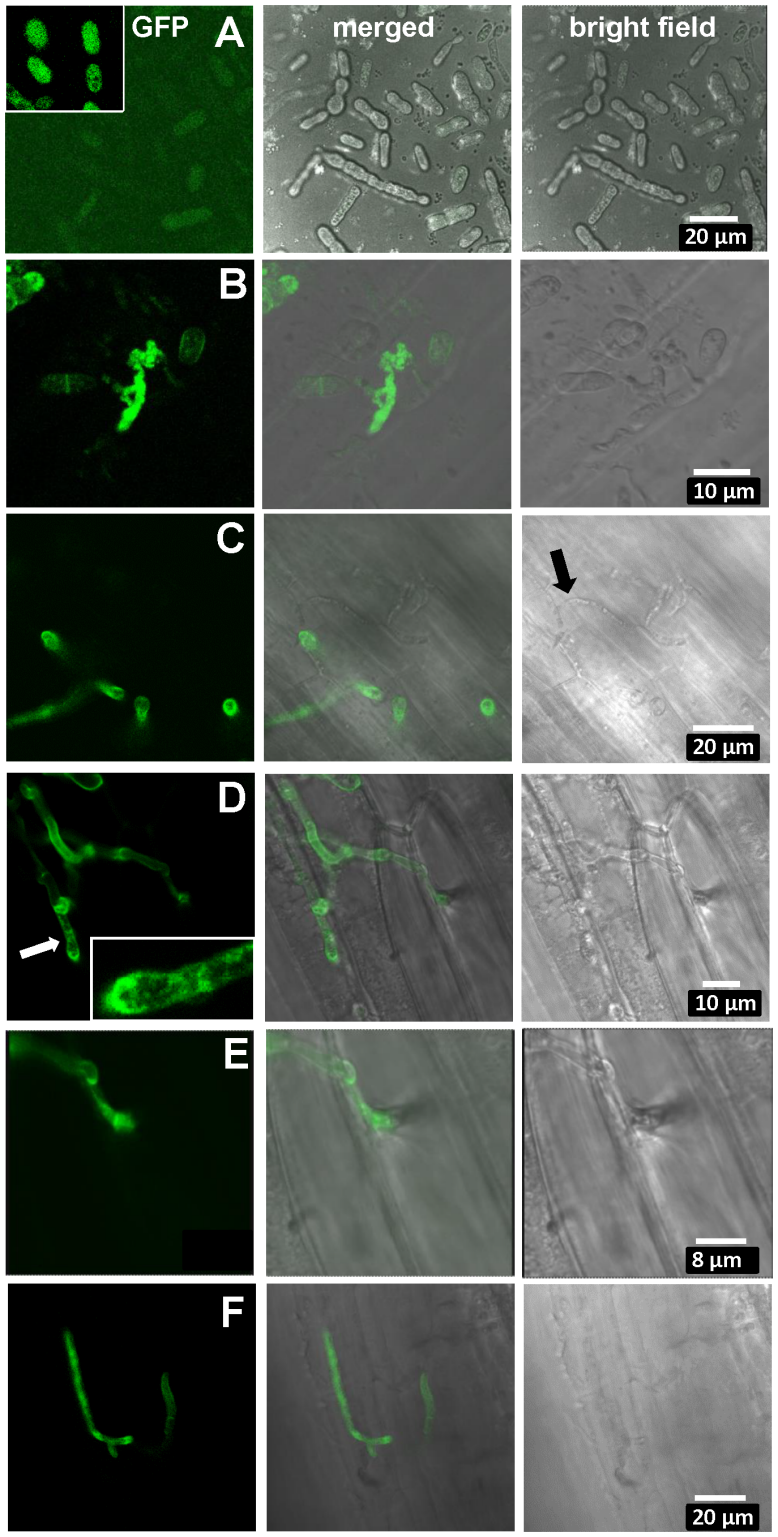
Expression of UhAVR1:GFP chimers during infection. Confocal microscopy of mated *U. hordei* strains transformed with various GFP constructs. **A.** Free-floating and mated cells of cross Uh1353×Uh362 (*MAT-2, Uhavr1*) showing no green fluorescence, whereas GFP expressed from the strong constitutive *otef* promoter in strain Uh364 (Uh1351) shows bright fluorescence (insert); protein blot analysis verified no expression from Uh1353 and strong expression of just GFP from Uh1351 under these conditions (**Figure S9B**). **B.** As a control, Uh1357 (*MAT-1* Δ*UhAvr1* [otef:*UhAvr1*:*GFP*])×Uh362 on compatible Odessa coleoptiles at 48 hai shows strong GFP expression from the *otef* promoter in the same recipient strain. **C.** Uh1353×Uh362 on compatible Odessa coleoptiles at 48 hai shows septated hyphae on the surface devoid of cytoplasm and not fluorescing (arrow) whereas in invaded dikaryotic hyphae expression of the UhAVR1:GFP chimer is induced from its native promoter upon host “sensing” and penetration. **D.** As in C but at 100 hai, showing extended, septated hyphae. Fluorescence is visible in the hyphal cell wall but appears punctuated seemingly in vesicle-like structures in the growing points being concentrated at the tip (insert). **E.** Enlargement from D of penetration site on the right. **F.** Same cross as in C induces *UhAvr1* expression in incompatible Hannchen at 48 hai, but no HR is seen.

Therefore, to substantiate the tentative expression results and to possibly localize UhAVR1p, a chimeric gene construct was made of *UhAvr1* with its native promoter but linked to a green fluorescent protein (GFP) moiety at its C-terminal end. This was then used to replace by marker-exchange the Δ*UhAvr1* deletion in strain Uh1289, thereby putting the chimer in its original expression site (**Figure S6C**). Confocal microscopy of this constructed strain Uh1353 (**Table S2**) clearly corroborated the qRT-PCR expression results since no fluorescence was observed in haploid or mated cells at the time of inoculation of barley coleoptiles (**Figure 6A**), whereas bright fluorescence comparable to GFP expressed from the strong *U. maydis otef* promoter [44] (**Figure 6B**) was apparent after 48 hrs in mated dikaryotic hyphae upon infection (**Figure 6C**) and while extending in the coleoptile later during the infection (**Figure 6D**). GFP fluorescence was seen in growing hyphal tips, possibly in vesicle-like structures (**Figure 6D and E**) and in older hyphae associated with the cell wall.

On cv. Hannchen, a very similar infection by fluorescent hyphae was observed but no obvious HR reaction, such as increased autofluorescence and/or cell collapse, was seen at 72 hrs (**Figure 6F**). This suggested that the chimeric protein seemed unable to trigger the *R* gene-based immunity. Indeed, pathogenicity tests with these complemented strains (Uh1353, Uh1354, Uh1355, **Table S2**) when mated with Uh362, were causing similar levels of disease on both Odessa and Hannchen (**Table S3**). In contrast, as reported above, strains complemented with wild-type *UhAvr1* gene sequence including promoter and terminator elements did not cause any disease on Hannchen (Uh1372, Uh1373, Uh1374; **Figure 2C** **, Table S3**). These experiments suggested that the C-terminal GFP-moiety interfered with the process that led to resistance triggering. We have not been able to verify in these strains whether or not the intact chimer is produced when infecting and if so whether possibly proper translocation and targeting to the proper location is affected by the presence of a C-terminal moiety. Whether C-terminal extensions interfere with protein structure and/or obstruct proper recognition of the host target(s), *R* gene or *R*-gene complex, needs further study but has been shown to occur in the flax rust fungus [45].

When attempting to complement virulent deletion strain Uh1289 (Δ*UhAvr1*) with the *UhAvr1* ORF under the control of the strong constitutive *Ustilago Hsp70* promoter, the resulting transformants, when mated with compatible parental strain Uh362, did not trigger resistance in cultivar Hannchen and yielded levels of disease similar as on Odessa or from control crosses (**Table S3**, crosses 19–21, compare cross 6). Similar constructs with the *Hsp70* or *otef* promoters driving the *UhAvr1* ORF now linked at its C-terminal end to either the HA epitope tag or a GFP moiety, yielded transformants that similarly gave comparable levels of disease on both Odessa and Hannchen (**Table S3**, crosses 4, 5, 13–18). Protein blot analysis confirmed the production of the expected chimeric proteins in the transformants (**Figure S9**) and we assumed from these assays that the wild-type UhAVR1p effector is similarly expressed from the *Hsp70* promoter in the transformants mentioned above. In many pathogens studied, cloned avirulence effectors have been shown to assert their avirulence function when reintroduced and expressed from non-native, strong promoters. In *U. hordei*, the expression of *UhAvr1* is finely tuned (**Figure 6**) and it is possible that this regulation is essential for proper relocation and function, including *R*-triggered immunity.

### UhAVR1p is not crucial for virulence

In several pathosystems, the deletion of avirulence effector genes was shown to affect virulence on host cultivars not harboring the cognate *R* gene. We tested in the ΔC18A2 deletion mutant whether or not genes 6 to 22, which included *UhAvr1* and two other CSEP genes, have any virulence functions in *U. hordei*. To this end, an equivalent C18A2 deletion was generated in a *MAT-2* mating partner by crossing Uh1041 (*MAT-1* Δ*C18A2*) with virulent parent Uh362 (*MAT-2 Uhavr1*) on barley cultivar Hannchen. Carboxin-resistant basidiospores of mating type *MAT-2* were collected by germinating teliospores from infected heads and lack of fragment C18A2 was verified by DNA blot analysis (**Figure S10A**). Each of three individual C18A2 deletion mutant progeny (Uh1116, Uh1117, Uh1118) was back-crossed with Uh1041, resulting in virulence towards Odessa that was similar to the wild-type cross (**Figure S10B**). One cross tested on Hannchen seemed also not affected in virulence compared to the single deletion mutant (**Figure 2A**). We concluded that genes 6 to 22 do not contribute significantly to virulence on barley. Δ*UhAvr1* mutants crossed with Uh362 (*Uhavr1*) are always included in our pathogenicity tests and over many experiments, virulence, expressed as number of plants infected per total number of plants inoculated, has not differed significantly from wild-type crosses. This suggests that effector UhAVR1p is not contributing significantly to virulence. It is difficult to express virulence in a quantitative manner in this pathosystem and a subtle advantage of expressing *UhAvr1* may play out at the population level over time.

### The *UhAvr1* locus resides in an evolving cluster of effectors in both *U. hordei* and *U. maydis*

**Figure 7:**
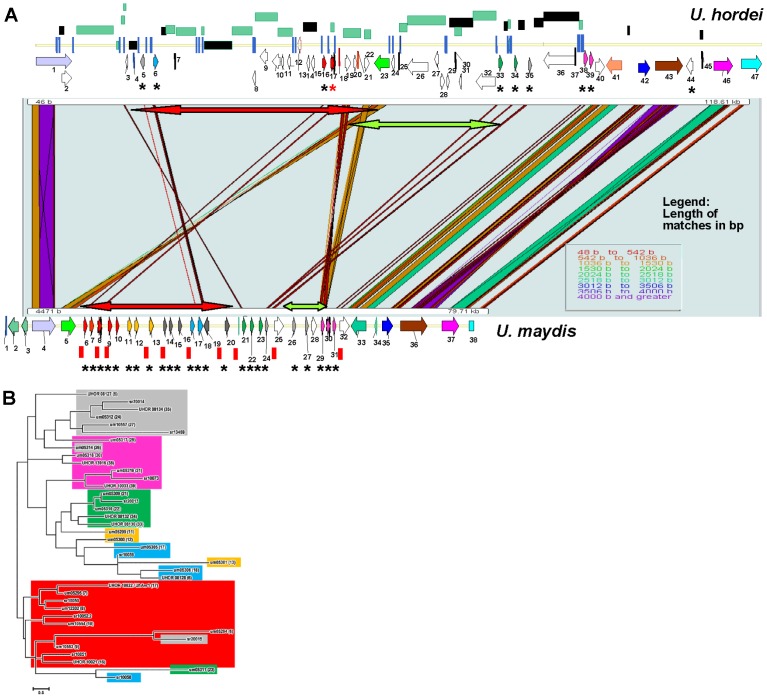
Comparison of the *UhAvr1* loci in the parental strains and to the syntenous *U. maydis* cluster of effectors. **A.** Comparison of the *UhAvr1* locus (top) to the syntenic region in *U. maydis* harbouring cluster 19A (bottom) to illustrate the gross rearrangements present in the two genomes. Both loci are drawn to scale and PatternHunter output [97] was used to visualize synteny. Arrows indicate the position and direction of transcription of the genes and asterisks indicate predicted CSEPs. Light and dark green rectangles represent regions with LTRs and repeats, respectively. The red and green-colored two-sided arrows represent two regions that are inverted. Blue vertical lines represent small repeats and red vertical bars represent the 10-bp repeats scattered over the *U. maydis* genome and suggested to be TE footprints [32]. Gene numbering in *U. hordei* is as in **Table S1**; gene 17 is *UhAvr1* (red asterisk in the top panel). Gene numbering in *U. maydis* is given in **Table S5**. **B.** Unrooted molecular phylogenetic tree of nine CSEPs found at the *UhAvr1* locus of strain Uh364, 24 predicted CSEPs in the syntenous clusters 19A in *U. maydis* [26], and selected homologs in *S. reilianum* [39], revealed likely family members. The color code reflects the paralogous and homologous groups in the three species which are only depicted for *U. hordei* and *U. maydis* in panel B. The evolutionary history was inferred by using the Maximum Likelihood method based on the JTT matrix-based model [98] conducted in MEGA5 [99].

The sequence analysis of clone BAC3-A2 revealed that the *UhAvr1* locus is orthologous to a region on *U. maydis* chromosome Chr 19, spanning a cluster of 24 CSEPs, called cluster 19A, the largest of such clusters in the *U. maydis* genome [26] (**Table S5**). A similar cluster is found in *S. reilianum*, harboring 29 CSEPs [39]. In *U. maydis*, deletion of this cluster resulted in reduced disease on maize seedlings. SIMAP analysis [46] and two-directional BLASTp searches were used to find orthologs for the *U. hordei* predicted CSEPs at this region in the *U. maydis* genome (**Table S1**). There is synteny with conserved gene order between these *U. hordei* and *U. maydis* genomic regions flanking the predicted CSEPs (**Figure 7A**). However, the region containing the CSEPs is much diverged and rearrangements, including changes of gene orientation and several translocations of genes within the cluster, are apparent. For example, *DigA* (*Uh* gene 1 and *Um* gene 4) is conserved but a homolog of the adjacent oligosaccharyltransferase gene (*Um* gene 5) is found 52 kb away in an inverted orientation in *U. hordei* (*Uh* gene 23). On the other end, conserved homologs of *U. maydis* genes 35, 36 and 37 are found in a syntenous region in *U. hordei* (genes 42, 43 and 46, respectively), except that a CSEP gene (*Uh* gene 44) with homology to two *Um* CSEPs that are however located on a different *Um* Chr 10, and repeat sequences have inserted.

Overall, the *U. hordei* cluster region in between the syntenic blocks bordered by *Uh* genes 1 and 40, is 40.6 kb larger than in *U. maydis*, in part the result of the presence of TE and repetitive DNA sequences. Other important differences are in the complement of the predicted CSEP genes. In *U. maydis*, four families of CSEP genes that are arranged in tandem in clusters of several paralogs, were described [26] (**Figure 7A**). One *U. maydis* family (genes *um05299*, *um05300* and *um05301*, genes 11, 12 and 13) is not represented in the *U. hordei* region or its genome [32] and seems species-specific. A molecular phylogenetic tree was generated and to reveal possible derived family members, we included several family members from the *S. reilianum* 19A CSEP cluster [39] (**Figure 7B**). *UhAvr1* (*UHOR_10022*, gene 17) and its adjacent paralog *UHOR_10021* (gene 16) are homologous to *U. maydis* CSEPs um05294 and um05295 (genes 6 and 7) residing in the *tin1-1* to *tin1-5* cluster, an expanded family of five adjacent, weakly related paralogous effectors [47]; *S. reilianum* has 3 homologs, sr10050, sr10051 and sr10052.2 (**Figure 7A and B**). Such an expansion in *U. maydis* is also seen for *UHOR_10033* (gene 39) and *UHOR_13916* (38) with three related paralogs in *U. maydis* (um05317, um05318 and um05319, genes 29, 30 and 31) and four in *S. reilianum* (sr10073, sr10075, sr10077 and sr10079), and for *UHOR_08134* (gene 35) with various homologs in both *U. maydis* and *S. reilianum*. Overall, in *U. hordei*, the related families are more dispersed and separated from adjacent genes, sometimes in inverted orientation, by TE and repeat sequences. Virtually no such repeat sequences are present in the *U. maydis* cluster although *U. maydis* gene um05316 (gene 28) codes for a transposase indicating possible (past) TE activity (**Figure 7A**).

## Discussion

Previously we showed that the *UhAvr1* locus was located to an approximately 80-kb region contained on BAC clone 3-A2 [38]. In this study, we sequenced its insert to discover, among others, ten ORFs encoding small predicted secreted proteins at this genetic locus. RAPD and AFLP markers limited these to seven most-likely candidate avirulence-triggering effector genes. Sequence comparison of these ORFs from the virulent and avirulent parents used to generate the mapping population, as well as from ten additional virulent and avirulent field isolates from a world-wide collection, revealed that the change from avirulence (*UhAvr1*) to virulence (*Uhavr1*) is not due to mutations in the ORFs or the presence or absence of ORFs in the two parental strains. We subsequently identified *UHOR_10022* through targeted deletions and complementation-based approaches as being *U. hordei* avirulence gene *UhAvr1*.

Quantitative RT-PCR analysis to verify expression of *UhAvr1* during infection proved challenging because of the very low levels of fungal biomass at this stage relative to the tissue mass in the coleoptile. However, when microscopically investigating single cell events, the substantial fluorescence emanating from the UhAvr1:GFP fusion transcribed from its native promoter in its original genome location, only shortly after contact with barley coleoptiles (**Figure 6**), showed that this gene is induced during infection. In RNA samples isolated from immature and mature infected seed heads, no *UhAvr1* mRNA could be detected by quantitative RT-PCR whereas high levels of expression were detected for *U. hordei* actin (UHOR_08813) and eIF-2B (UHOR_07772) genes that were used as references in the analysis (data not shown). This shows that *UhAvr1* is expressed only during early infection, being highly regulated and suggests that UhAVR1p is needed only early during infection. In many plant pathogenic fungi and oomycetes, a subset of predicted effectors, some of which trigger avirulence, are expressed only upon infection and sometimes only in specific infection structures such as appressoria or haustoria [26], [45], [48]–[51].

Expression of *UhAvr1* provides complete immunity in barley cultivars harboring *Ruh1* and we show that UhAVR1p harbors an avirulence function which is somehow recognized by RUH1. Previously we had shown by electron microscopy that this interaction caused hyphal restriction, likely due to the deposition of electron-dense material, and necrosis in cells immediately surrounding penetration sites early upon infection [42]. This correlates well with the timing of expression of *UhAvr1* and the accumulation of callose and associated fluorescence just around penetration sites and around restricted hyphae within 72 hours of infection (**Figure 4C**).

Sequence analysis among the limited collection of field isolates virulent and avirulent on Hannchen, suggest that *UhAvr1* may encode a rather monomorphic protein; only two point mutations were identified in *UhAvr1*. In only one avirulent strain Uh813 that translated into a single amino acid substitution. Whether this points to indirect recognition of this avirulence effector by RUH1, more in line with the ‘guard model’ stipulating purifying selection as the guard recognizes modifications of the AVR protein on the guardee and imposes selection pressure against its function [52], [53] and which favours gene inactivation or deletion [54], whereas direct interaction according to the ‘receptor-ligand model’ tends to result in diversifying selection that generates highly divergent avirulence effector alleles in pathogen populations to escape this recognition by the *R* gene products [55]–[58], remains to be investigated. Future experiments are geared towards finding the target and mode of interaction of UhAVR1p.

In UhAVR1p, no clear similarities could be found to known proteins or domains. Interestingly, a RxLR tetrad is found in the paralogous *U. maydis* effectors um05295 (amino acid positions 99–102) and um10554 (125–128), and sr10052 (89–92) from *Sporisorium reilianum*. When compared, the RxLR motifs line up with a PDFR tetrad in UhAVR1 (**Figure S8**). The RxLR motif has been proposed to be involved in binding of specific plant and mammalian cell wall phospholipids (phosphatidylinositol 3-phosphate or PI3P), mediating effector uptake. However, among various fungal and oomycete effectors, this motif has been shown to allow for some variation [59] and its function in uptake has been controversial [60], [61]. Alternatively, PI3Ps are enriched in intracellular organelle membranes, specifically from early endosomes [62], [63] and we are investigating possible targeting of UhAVR1 to such locations. Intriguingly, if 20 amino acids were cleaved off, UhAVR1 is predicted to be myristoylated, suggesting a membrane association is involved. Several effectors have been shown to be myristoylated and that this was required for function [64], [65]. Moreover, amino acid K39 has a high probability of being a sumoylation site (**Figure S8**). Sumoylation is a reversible post-translational modification that affects an increasing number of biological processes by altering intracellular localization and protein-protein interactions.

We were not able to ascertain the virulence function(s) of *UhAvr1* in this study. Examples exist of avirulence effectors with a clear role in virulence, such as AVR-a10 and AVR-k1 from *Blumeria graminis* that enhance fungal penetration in barley epidermal cells [66]. Similarly, AVR3a from *P. infestans* can suppress necrotic responses in *Nicotiana benthamiana* induced by INF1 elicitor [67]. Experiments expressing *UhAvr1* in *Nicotiana* leaves did not support a role in the suppression of cell death initiated by several elicitors. This could be due to unavailable or too diverged targets of UhAVR1p in this heterologous system. A homologous system in barley would be needed, possibly in young coleoptiles if timing of expression is essential. In this context it is important to note that infection of barley by *U. hordei* only occurs early at seed germination, since the fungus needs to reach meristematic tissue; older plants or leaves cannot be successfully inoculated. In the related study by Brefort *et al.* [47], a *U. maydis* strain lacking the Tin1-1 to Tin1-5 effectors (genes 6–10 in **Figure 7**, with UhAVR1p closest related to Tin1-2) caused strong induction of endochitinases, SA-binding proteins and the apoplastic peroxidase POX12 in maize, indicative of enhanced defense responses and a possible role for these effectors in suppressing basal host immunity.

Whatever function UhAVR1p has, it does not seem to contribute significantly to virulence as shown in **Figure 2B** (and **Figure S10B**, where the paralogous gene 16 is also deleted). In *U. maydis*, deletion of the paralogous *tin1-1* to *tin1-5* effector family did not cause a statistically significant reduction in virulence [47]. However, it is very difficult to assess relative infection rates in this pathosystem for which no good quantitative measures exist and which relies on the number of infected plants out of a significant number of inoculated plants showing often considerable variation. Functional redundancy may exist in effectors located at other sites in the genome such as effector gene *sr13459*, a potential homolog of *UHOR_08134* (gene 35), which is located on a different Chr 20 in *S. reilianum*. Although not easily measurable as reduced virulence in a few plant experiments, on a population level the UhAVR1 effector may contribute to overall fitness or virulence. It has also been argued that effectors (alleles) that contribute to virulence or fitness are maintained in a pathogen population [e.g. 68–70]. A TE insertion inactivated *UhAvr1* but in the isolates we investigated, the genetic information of the ORF was still present. It is therefore possible that virulent isolates that have retained the (inactive) *UhAvr1* ORF sequences may have a selective advantage because subsequent re-activation of the *UhAvr1* ORF, i.e., by hooking it up again behind a promoter through transposition or gene conversion, will again bring about this population-level advantage if the selection pressure, i.e., plants with *Ruh1*, disappeared from the environment the fungal population occupies.

Our analysis of the *U. hordei* genome revealed many TEs and repeats with Repeat-Induced Point mutations, likely inactivating them [32]. However, complete TE (LTR-like) sequences with intact conserved predicted (gag/pol) proteins were also found indicating that these elements could be active transposons. In addition, comparison of the genomes of the three smuts, *U. hordei*, *U. maydis* and *Sporisorium reilianum*, suggested that a recent expansion had occurred of a few related TEs newly introduced in the *U. hordei* lineage after separation from a common ancestor, also indicating active elements (at least in its recent evolution). In our study, sequence comparisons between *UhAvr1* loci from isolates avirulent and virulent on Hannchen revealed TE sequence variants upstream of *UhAvr1* and that virulence towards *Ruh1* was the result of TE activity and insertion of TE-derived sequences in the promoter region of *UhAvr1* changing expression and likely recognition. TE activity and insertion at avirulence effector loci causing *in situ* mutations or changes in transcription leading to virulence phenotypes, have been described before in ascomycete pathogens [e.g. 19,21,71–73] but not in basidiomycetes.

At the locus, sequence variation involving TE sequences among various field isolates indicated transposition events, possibly of independent nature suggesting TE activity is an important mechanism to overcome resistance to *Ruh1*. In some field isolates, sequence variation was identical such as in virulent strains Uh362, Uh805, Uh815 and Uh820 (**Figure S3C**). Considering the geographic area the latter three were collected from (Kenya, Canary Island and Tunisia, respectively), this likely reflects a common ancestral event and regional spread; Uh362 was derived from a Canadian isolate backcrossed with an African isolate long ago to obtain homozygous material and likely acquired the virulent allele from this region. Similarly, the identical 340-bp insertion found in isolates Uh813, Uh1273 and Uh1283 (**Figure 3C**), respectively from Iran, Azerbaijan and Turkey, could have a regional ancestral origin. This illustrates the difficulty of sampling pathogens from a crop plant that is widely traded and grown in certain areas. In order to assess more-comprehensive variation, one would need to sample isolates from truly wild barley populations in remote locations.

TEs play important roles in shaping genomes, causing rearrangements such as deletions, inversions, duplications, translocations, but also neo-functionalizations. Recently, genome analyses of several fungal and oomycete pathogens revealed that many effector genes reside in TE and repeat-rich regions (including at telomeres), a feature that may have evolved to allow for variations necessary for parasites under high host selection pressures to quickly adapt when their virulence effectors are triggering defenses [e.g.], [18], [22], [68], [74]–[77]. The *UhAvr1* gene is located in a region of the genome that sports ten CSEP genes and is, with the mating-type region, among the richest in repeats and TEs, approaching 50% (**Figure 7A** [32], [41]). Incidentally, the *UhAvr1* locus revealed conserved synteny in regions flanking cluster 19A, the largest cluster of CSEP genes in *U. maydis*, and to some extend among its coded effectors (**Figure 7**). Transcription of the *U. maydis* CSEP genes is induced after infection of maize and deletion of this whole cluster severely reduces disease [26]. It appears that these species, including related *S. reilianum*, share some of these likely ancestral genes but that possibly because of their obligate biotrophic interaction with diverse hosts, these effectors have evolved differently. Phylogeny revealed expanded CSEP gene families in *U. maydis* and *S. reilianum*. Interestingly, in the *U. maydis*-maize pathosystem, no effector-*R* gene interactions involving avirulence and resistance genes have been genetically identified to date [78]–[80]. It is possible that the higher number of paralogs in the *U. maydis* (and *S. reilianum*) effector gene families represent past diversifying selection acting on these effectors to avoid host recognition and making *U. maydis* better adapted to host populations. This could have resulted from adaptation to changed effector target molecules or the defeat of major resistance genes over time.

While in *U. hordei* the mechanism to avoid host recognition involves the activity of TEs, *U. maydis* and *S. reilianum* have more streamlined genomes with few deleterious repeats and TEs [26], [32], [39]. The question arises how the latter organisms have created the needed variation. One scenario could be past TE activity, followed by purging of TEs and repeats brought about by a highly active homologous recombination system known to exist in *U. maydis*. The numerous small (10 bp) repeats in the *U. maydis* genome have been suggested to be footprints of past TE activities [32], [39] and 26 are found exactly in between the effector genes in the *U. maydis* cluster 19A (**Figure 7A**). Alternatively, if TE activity did not play role in these organisms, highly active recombination followed by genetic drift may have caused sufficient variability. However, the evolution of these pathogens is more complex and involves sex [81]; *U. hordei* with its bipolar mating system which promotes inbreeding, may select for the use of TEs as genome modifiers whereas *U. maydis* and *S. reilianum* with their tetrapolar mating systems which cause reduced inbreeding potential, can create variation through recombining with outside partners [77]. Undoubtedly, the selection pressure imposed by the host has had a major impact on maintaining the variability among populations, as has been shown for the *U. maydis*-maize interaction [82].

## Materials and Methods

### Plant and fungal strains

Two barley cultivars, ‘Odessa’ (*ruh1*, universal susceptible) and a differential, ‘Hannchen’ (*Ruh1*) were used for pathogenicity assays. Fungal strains and mutants generated are listed in **Table S2**. *U. hordei* haploid parental strains Uh364 (alias Uh4857-4, *MAT-1 UhAvr1*) and Uh362 (alias Uh4854-10, *MAT-2 Uhavr1*) were described previously [38].

### Fungal growth conditions and *U. hordei* transformation

Haploid *U. hordei* strains were grown in liquid Potato Dextrose Broth (PDB), complete medium (CM [83]) or YEPS (1% yeast extract, 2% peptone, 2% sucrose), while 2.5 µg/ml carboxin (Sigma-Aldrich), 100 µg/ml Hygromycin B (Calbiochem, La Jolla, CA, USA) or 40 µg/ml Zeocin (Invitrogen, Valencia, CA, USA) were added when appropriate. Strains were grown at 22°C. For genetic transformation of *U. hordei*, protoplasts were prepared according to a modified protocol [84], instead using 384 mg/ml Vinoflow FCE (Gusmer Enterprises) as enzyme mix for digesting the fungal cell wall [85]. Protoplasts were transformed with 5 µg DNA mixed with 1 µl of a 15 mg/ml heparin (Sigma) in STC (10 mM Tris-HCl pH 7.5, 100 mM CaCl_2_, 1M sorbitol) solution and selected on double-complete medium plate (DCM) supplemented with 1 M sorbitol and appropriate antibiotic. After 5–7 days incubation at 22°C, colonies from DCM-S were transferred to CM medium and incubated for two days at 22°C before transferring to liquid CM medium for further analysis.

### Pathogenicity assays

Two haploid cultures of opposite mating type (OD600 of ∼1, tested in mating assays as described in [86]) were mixed 1∶1 v/v before inoculation of barley seeds. Seeds were dehulled, surface sterilized for 3 min with 70% EtOH, followed by 10 min with 1% bleach, and rinsed several times with sterile ddH_2_O. Surface-sterilized seeds were dipped in mated cultures and a vacuum of 20 psi was applied for 20 min. Subsequently, excess inoculum was drained and seeds were kept for 6 hrs at room temperature before sowing in potting mix (Pro-Mix BX) at a density of 3 seeds per 3×3″ pot of which 18 were placed in a tray. Plants were grown in controlled-environment chambers with an 18 hour light-6 hour dark cycle at 22°C. Disease ratings were scored at heading, approximately 2 months after planting, by counting infected plants among all inoculated plants. The same inoculum was always applied to both barley cultivars Hannchen (*Ruh1*) and Odessa (*ruh1*) simultaneously to verify effectiveness.

### Sequencing and analysis of BAC clones and ORFs

BAC3-A2 containing the *UhAvr1* locus from the avirulent parent Uh364 was sequenced using the GPS-Mutagenesis System (New England Biolabs) with a few modifications. In the donor vector, the kanamycin resistance cassette within the transprimer was replaced with a phleomycin resistance cassette driven by both the Em7 bacterial promoter and the *U. maydis* glyceraldehydes-3-phosphate dehydrogenase (GAPDH) promoter and terminator [87]. This generated an insertion that could be used directly as a marker-exchange construct to generate deletions within *U. hordei* through homologous recombination. After *in vitro* recombination and transformation in *E. coli*, BAC clones from 6×96 random bacterial colonies were sequenced using primers N and S, yielding paired sequence reads from the ends outwards of the randomly inserted transprimers. These DNA sequences and several BAC end-sequences covering this region from clones of the source BAC genomic library [41] were entered in the PCAP.REP genome assembly program [88]. To place certain sequences and to verify their location, physical mapping was performed by using the unique Not1 restriction enzyme sites in the transprimer and BAC insert and measuring generated fragment sizes on CHEF gels (data not shown). BAC clone 1-E2 covering the *Uhavr1* locus in the virulent parental strain Uh362, was recovered from a BAC library via hybridization. This BAC clone, as well as BAC3-A2 for confirmation, were sequenced using the 454 technology at the Plant Biotechnology Institute (Saskatoon, SK). The resulting reads were assembled using the Newbler program (Roche Applied Science). Alignment of the BAC sequences from the virulent parent along the avirulent backbone was facilitated by a custom Perl script. The order of contigs was confirmed by PCR and gaps were corrected through manual sequencing. Genes were predicted using FGENESH [89] and VectorNTI (Invitrogen). Predicted proteins were searched for secretion signals using the SignalP 3.0 Server (http://www.cbs.dtu.dk/services/SignalP/), by TargetP v1.1 [90] to identify and remove proteins that were predicted to be mitochondrial, and by ProtComP 9.0 (http://linux1.softberry.com/berry.phtml) which compares them to proteins in the LocDB and PotLocDB databases which hold proteins with known or reliably predicted localization. The sequence of clone BAC3-A2 was contributed to the Uh364 genome sequencing effort ([32] http://www.helmholtz-muenchen.de/en/ibis/institute/groups/fungal-microbial-genomics/resources/muhdb/index.html) and is part of UHOR_scaffold_5.00017, NCBI #CAGI01000148.1 with UHOR_10022, protein ID CCF49778.1, at position 159450–160022; the sequence of the region containing the breakpoint in virulent parent Uh362 on BAC clone1-E2 is accessible under NCBI #KF640593. To sequence ORFs in Uh362 and field isolates, predicted CSEP genes and intergenic regions were amplified by PCR. Primers were designed 100 bp upstream and 100 bp downstream of the ORFs (**Table S4**) using the Primer3 software (http://sourceforge.net/projects/primer3/files/). Sequencing of the purified products was carried out using the Big Dye terminator v3 chemistry (Applied Biosystems). Large PCR products were generated using LongAmp DNA Polymerase (New England Biolabs, M0323S).

### Deletion analysis of the *UhAvr1*-containing region

One gene, *UHOR_08134*, was deleted using a double-jointed PCR method [91] and the hygromycin resistance cassette to generate a marker-exchange construct. All other deletion mutants involving individual target genes or clusters of genes were constructed using marker-exchange plasmids generated by the DelsGate method [92]. Briefly, primers were designed separately for each construct to amplify by PCR 1.5 to 2 kb of 5′- and 3′- sequences flanking the target region (**Table S4**), using Uh364 genomic DNA as template. Primers 5L and 5R were then used for the amplification of a 5′-flanking fragment adding an I-SceI recognition sequence tail upstream and an *attB1* sequence tail downstream of the flank sequence. Primers 3L and 3R were used to amplify the 3′-flanking fragment, adding the *attB2* sequence tail upstream and the I-SceI sequence tail downstream. The two PCR-amplified fragments were then gel-purified using the QIAquick Gel extraction kit (Qiagen) and subsequently recombined into the pDnorCbx vector (NCBI accession # EU360889 [92]) using the Gateway BP Clonase II enzyme Mix (Invitrogen). To assess the resulting marker-exchange plasmids, two PCR reactions were performed using 5′- gene-specific primer 5R in combination with the SceIF primer, and 3′- gene-specific primer 3L in combination with primer SceIR primer (**Table S4**). SceIF and SceIR primers were designed for the I-SceI enzyme recognition site in the forward and reverse orientation, respectively. The deletion constructs were verified by sequencing and were then linearized with I-SceI enzyme (New England, Biolabs) and used directly for *U. hordei* transformation.

Carboxin-resistant mutants were analyzed for proper gene deletion by PCR reactions on purified gDNA. Sixty to as many as 300 carboxin-resistant colonies sometimes needed to be screened (depending on the region targeted) to get at least four PCR positive transformants for each construct which were then verified by DNA blot analysis. For DNA blot hybridization, 10 µg of gDNA was digested with selected restriction enzymes and run out in 0.8% agarose gels in 1xTAE buffer (40 mM Tris-acetate, 1 mM EDTA). Blotting to nylon membranes (Amersham Biosciences, Buckinghamshire, UK) and hybridization were carried out following standard procedures [93]. DNA probes for either the 5′- or 3′-flanks were amplified using PCR and labeled with [α-^32^P] dCTP using the random primer labeling system kit (Amersham Biosciences) according to manufacturer's recommendations. The efficiency of homologous recombination was different for different constructs and seemed dependent on the size of the deletion fragment; the efficiency was higher for small fragments.

### Plasmid constructs

Gene expressing constructs were designed to make use of the GateWay technology (Invitrogen). *U. hordei* ORFs, either with or without the sequence coding for the SP, but without their stop codon, were amplified by PCR with a CACC tetranucleotide sequence at the 5′-end to allow for directional cloning into Gateway entry vector pENTR/D-TOPO (Invitrogen; **Table S4**). Cloned inserts were sequenced and were subsequently transferred to a designed GateWay destination vector, pUBleX1Int:GateWay:HA (a derivative of *Ustilago*-specific integrative expression vector pUBleX1Int [94]), using LR recombineering. For the transient assays and microscopy after bombardment, the above-mentioned pENTR clones (UHOR_10022-SP-STOP) were recombined into a modified pMCG161 vector (ChromDB at http://www.chromdb.org; NCBI accession no. AY572837) to create N- or C-terminal GFP-expressing chimers from the maize ubiquitin promoter (Ubi:GFP:UhAvr1-SP and Ubi:UhAvr1-SP:GFP). A control construct expressed just GFP. Details on the constructs and destination vectors can be obtained from the authors.

### Quantitative RT-PCR analysis

Barley cv. Hannchen coleoptiles were inoculated with mated cell cultures as described above and infection was allowed to proceed for 48 hrs on sterile filter paper in petri dishes in the dark at 22°C. Coleoptiles from 3 biological replicates were dissected from the seed and roots and total RNA from 100 mg of sample was isolated using Trizol Reagent (Invitrogen, Cat. No. 15596-018). Ten µg of total RNA was then treated with TURBO DNase (Applied Biosystems, Cat. No. AM22380. After quantitation, cDNA synthesis was carried using SuperScript III Reverse Transcriptase (Invitrogen, Cat. No. 18080-093). Quantitative RT-PCR assays were carried out on a CFX96 Real-Time System (Bio-Rad) with the following cycling conditions: (1) 2 min 95°C incubation, (2) cycling at 95°C 10 sec, 55°C 30 sec for 40 cycles, (3) melt curve from 65°C to 95°C at 0.5 degree increments. Analyses and statistics were carried out with the Bio-Rad CFX Manage Software. To overcome the very low expression levels observed, nested real time PCR was carried out as per [43]. An initial 10 cycle pre-amplification with flanking primers 1689+1249 for *UhAvr1* and 1804+1805 for reference gene *UheIF-2B* ( = UHOR_07772; **Table S4**) was carried out on a Bio-Rad MyCycler (conditions: (1) 95°C 2 min., (2) 10 cycles of 95°C 30 sec, 55°C 30 sec, 72°C 60 sec), followed by the qRT-PCR process above performed with nested internal primers 1798+1799 for *UhAvr1* and 1811+1812 for reference gene *UheIF-2B* (**Table S4**).

### Protein blot analysis

Total protein was isolated from frozen ground cells, as described [95]. Protein samples were boiled for 5 min and spun briefly for 30 sec before being separated by 12.5% SDS-PAGE on a Bio-Rad Mini-Protean III apparatus. Protein was transferred from the gel to Sequi-Blot PVDF Western blotting membrane (Bio-Rad) using a Bio-Rad liquid transfer apparatus following the manufacturer's protocols. Membranes were probed with 200 ng/ml rat anti-HA (hemagglutenin) high affinity monoclonal antibody (Roche Applied Science) or anti-GFP (Clontech Living Colors JL-8 anti-GFP monoclonal). For detection of primary bound antibody, membranes were incubated with peroxidase-conjugated AffiniPure Goat Anti-Rat-Ig (H+L) secondary antibody according to supplier's instruction. For visualization of bound antibody, the Enhanced Chemiluminescence system (ECL) plus Western Blotting Detection Reagents (Amersham Biosciences/GE Healthcare) were used.

### Microscopic analyses

To inoculate barley coleoptiles with teliospores, seed hulls were removed by hand to expose the embryo. Seeds were surface sterilized as above and germinated for 48 hrs in the dark at 18°C on sterile filter paper. Emerged coleoptiles were then dusted gently with a paintbrush with teliospores previously released from an infected seed head by gentle grinding. Alternatively, seeds germinated for 24 hrs with emerged coleoptiles, were immersed in cell cultures of OD600 ∼1, mated for 24 hrs after mixing *MAT-1* and *MAT-2* strains in a 1∶1 ratio, under a vacuum of 20 psi for 20 min, after which the inoculum was drained. After inoculation, seedlings were kept moist and were further incubated in the dark at 18°C. Observation of GFP-expressing fungal infection was done on a Leica SP2-AOBS laser scanning confocal microscope at 488 nm excitation and detection at 499–552 nm.

For light microscopy, seedlings were sampled at 72, 96, 120 and 144 hrs following inoculation. Plants were gently washed and crown tissues consisting of a 1 to 2 cm section of the coleoptile surrounding the crown region were excised, split longitudinally in half and both halves were mounted in lactophenol-cotton (aniline) blue to stain for callose [96]. Sections were viewed with a Zeiss Universal microscope using the 330–385 nm and 460–490 nm excitation and emission filters, respectively, and a HBO103W/2 light source. For detection of the oxidative burst, hydrogen peroxide was detected by vacuum infiltrating dissected coleoptiles for 10 min with 1 mg/ml 3,3′- diaminobenzidine tetrahydrochloride (DAB, Sigma) in 10 mM Na_2_HPO_4_, pH 7 and 0.05% v/v Tween 20, incubation for 6 hrs, and subsequent bleaching in a 3∶1∶1 ethanol ∶ acetic acid ∶ glycerol solution. The numbers of DAB stained sites and their relative size on both halves of 1 cm coleoptile sections were counted from a minimum of 5 seedlings per replication. Three replications were employed and the study was repeated two times. For quantitation of callose, average fluorescence associated with penetration sites was measured on 5 (compatible interaction) to 11 (incompatible interaction) TIF images imported into ImageJ software (National Institutes of Health, Bethesda, Maryland) and the average background fluorescence was subtracted. Data were analyzed using PROC GLM with SAS software (SAS Institute, Cary, NC, USA) and means were separated using Duncan's multiple range test (P≤0.05).

## Supporting Information

Figure S1 ***Ustilago hordei*** **life cycle.** **A.** Diploid (2n) teliospores are survival structures. When conditions are right, they germinate, in nature often under the hull of healthy seed that germinate at the same time. **B.** During teliospore germination, meiosis occurs and four haploid basidiospores are formed on the basidium. **C.** Mating type segregates 1∶1 (*MAT-1* ∶ *MAT-2*). Cells of opposite mating type can sense each other through the action of pheromones and pheromone receptors upon which each partner forms thin mating hyphae. When mating hyphae meet, fusion takes place whereby the dikaryotic state, characteristic for basidiomycetes, is reconstituted. This fusion also brings together the *b* mating type gene products from each mating specificity, bW1 and bE2 (and bW2 and bE1) from the respective partner, which form a hetero dimeric protein. This dimer can now regulate transcription of a large number of genes involved in the switch from budding growth to filamentous, pathogenic growth able to infect host tissues. The formed dikaryotic hyphae represent the biotrophic cell type that requires the barley host for completion of the life cycle; haploid cells are saprobic, non-pathogenic and can be manipulated in the lab. Dikaryotic hyphae grow over the surface of the germinating barley coleoptile, led by a cytoplasm-filled growing point and leaving behind septated empty hyphae, until direct penetration through a swelling at the hyphal tip (an appressorium-like structure) leads to infection of epidermal cells. Infection can only occur at early seed germination. **D.** Hyphae grow inter- and intracellularly, penetrating cell layers to reach the meristematic region of the growing point. Without causing visible symptoms, the fungus only starts proliferating once the barley meristematic region develops into seed spike tissue: **E.** β-glucuronidase -expressing fungus stained with X-gluc). **F.** Cells round off and form spore walls. **G.** Massive sporulation takes place in the developing head where seeds are replaced. Sometimes flag leaves develop pustules with teliospores.
(PDF)

Figure S2 **Identification of the** ***U. hordei*** **chromosomes and Not1 fragments harboring UHOR_10022 sequences in strain Uh364, avirulent, and strain Uh362, virulent on cv. Hannchen.** **A.** Chromosomes were separated on a CHEF gel as described [41] with sizes of yeast chromosomes in kilobases (kb) as markers on the left. *UhAvr1* has been identified on chromosome 18 (chr18) of an estimated 667 kb in isolate Uh364 [32]. EtBr, Ethidium bromide-stained agarose gel; autorad, DNA blot of the corresponding gel on the left, hybridized to ^32^P-labeled gene fragments as indicated (**Figure 1**). Three separate gel panels were used. **B.** CHEF gel of Not1-digested gDNA fragments revealing a polymorphism by the *UhAvr1* gene as a probe.
(PDF)

Figure S3 **DNA sequence comparisons of alleles from** ***U. hordei*** **isolates from various genes at the** ***UhAvr1*** **locus.** DNA sequence alignments in ClustalX covering: **A.** gene 16 (UHOR_10021), **B.** the gene 16-*UhAvr1* (UHOR_10022) intergenic region in avirulent strains, **C.** the gene 16-*UhAvr1* intergenic region in virulent strains, **D.** gene *UhAvr1*, **E.** gene 30 (UHOR_08130), **F.** gene 34 (UHOR_08132), **G.** gene 38 (UHOR_13916), and **H.** gene 39 (UHOR_10033), among various isolates. The highlighted asterisks or sequences indicate the ORFs, and the highlighted isolate names indicate an *UhAvr1* genotype (see **Table S2**).
(PDF)

Figure S4 **Deletion analysis of the** ***UhAvr1*** **locus.** **A.** The three overlapping bars (C18A2, C18A4 and C18A3) represent the fragments (with their sizes in kb) that were deleted in the three independent deletion mutants, respectively. The genomic region of the parental avirulent strain Uh364 with all predicted genes is given above (see **Figure 1**). **B.** DNA blot analysis of genomic DNA of C18A2 transformants, digested with XhoI. One of the transformants, number 5, revealed a band of 5.4 kb expected for the correct deletion mutant and was used for pathogenicity analysis; transformants 1 to 4 revealed both wild-type (wt) and the deletion construct fragments, indicative of an ectopic integration event. **C.** DNA blot analysis of genomic DNA of C18A3 transformants, digested with *Sal*I. All transformants revealed a fragment of 5.8 kb expected for the correct deletion mutant. **D.** DNA blot analysis of genomic DNA of C18A4 transformants, digested with AvaI. Transformant 3 revealed a band of the expected size of 7.2 kb to replace the wild-type fragment and was used for pathogenicity analysis. The cartoon above each gel is a schematic representation of the wild-type region in Uh364 (left) and deletion mutant (right). The probe used for each individual analysis was part of the 3′-flanking fragment of the deletion construct used and is indicated as a solid blue line in these cartoons.
(PDF)

Figure S5 **Deletion analysis of fragment C19A2 and pathogenicity tests.** **A.** The five overlapping bars (C18A2-a to e) represent the fragments (with their sizes in kb) that were deleted in the five independent deletion mutants, respectively. The genomic region of the parental avirulent strain Uh364 with all predicted genes is given above (see **Figure 1**). **B.** DNA blot analysis of genomic DNA of C18A2-a transformants, digested with BglII. Three of the transformants (lanes 1, 3 and 4) show a band of 2.7 kb expected for a proper gene deletion compared to the 2.1 kb fragment present in the wild-type Uh364 strain (wt). **C.** DNA blot analysis of genomic DNA of C18A2-b transformants, digested with BglII. One of the transformants (lane 2) shows a band of 3.2 kb expected for a proper gene deletion. **D.** DNA blot analysis of genomic DNA of C18A2-c transformants, digested with BglII. All transformants contained a band of 3.6 kb expected for a proper gene deletion. **E.** DNA blot analysis of genomic DNA of C18A2-d transformants, digested with HindIII. Four transformants (lanes 1, 5, 6 and 7) show a band of 6.2 kb expected for a proper gene deletion. **F.** DNA blot analysis of genomic DNA of C18A2-e transformants, digested with PstII. Three transformants (lanes 1, 5 and 6) show a band of 1.4 kb expected for a proper gene deletion. The cartoon above each gel is a schematic representation of the wild-type region in Uh364 (left) and deletion mutant (right). The probes used for each individual analysis were part of the 3′- (3F) or 5′- (5F) flanking fragment of the respective deletion construct used and are indicated as a solid blue line in these cartoons. **G.** Pathogenicity test of the deletion mutants (two per deletion as indicated on the X-axis). All mutants were crossed with Uh362 (*Uhavr1*). Mutants deleted for fragments C18A2-c and C18A2-d were virulent towards Hannchen, shown by red bars in the figure, indicating that the functional *UhAvr1* gene is located on these fragments. All other deletion mutants and the wild-type cross (Uh364×Uh362) were virulent towards Odessa (as a control for infection) and avirulent towards Hannchen which showed that they had an intact *UhAvr1* gene. Y-axis, percent infected plants out of the total number of inoculated plants. Average of three independent inoculation experiments with standard deviation is shown as error bars.
(PDF)

Figure S6 **Construction of Δ** ***UhAvr1*** **and** ***UhAvr1:gfp*** **chimeric replacement mutants.** **A.** Schematic representation of the deletion mutant with the red bar representing the 3′-part of *UhAvr1* that was deleted; the blue bars represent the flanks used in the deletion construct. **B.** Cartoon showing the 3′-end of *UhAvr1* replaced by the carboxin resistance gene (Cbx^r^) and DNA blot analysis of several transformants; total gDNA was digested with BglII and the blue bars indicate the probe. Two of the deletion mutant strains showing a band of expected size of 3.6 kb were used in pathogenicity tests. **C.** Cartoon showing the replacement construct which reconstitutes the complete *UhAvr1* ORF minus STOP codon while linking a GFP moiety to the C-terminus; Cbx^r^ is replaced by zeomycin resistance (Zeo^r^). DNA blot analysis of several transformants; total gDNA was digested with BglII and the blue bar indicates the probe.
(PDF)

Figure S7 **Clone BAC1-6 restores avirulence to the virulent C18A2 deletion mutant.** **A.** The 38.5 kb-fragment C18A2 deleted in the respective mutant Uh1041 is enlarged to show the location of the different ORFs; asterisks indicate the predicted CSEPs (compare **Figure 1**). The position of the complementing 11.5 kb-fragment in BAC1-6 clone is represented by a black line; the overlap contains two predicted CSEPs: gene 16 (*UHOR_10021*) and gene 17 (*UhAvr1*). **B.** Pathogenicity test of the deletion mutant strain (Uh1041) complemented with BAC1-6 as indicated on the X-axis. All mutants were crossed with Uh362 (*Uhavr1*). While the deletion mutants are fully virulent towards Hannchen, the complemented strains cause very low disease on Hannchen even though they are fully virulent towards Odessa (as a control for infection). This indicates BAC1-6 harbors *UhAvr1*. The Y-axis shows the percent of infected plants out of the total inoculated plants. The data shown here is an average of three independent experiments with standard deviation as the error bar.
(PDF)

Figure S8 **Identification of specific domains in UhAVR1p and comparison to other Ustilaginaceae effectors.** **A.** Secondary structure prediction using SWISS-MODEL. **B.** A CLUSTAL 2.1 multiple sequence alignment of UhAVR1p and three effector homologs from *U. maydis* and *Sporisorium reilianum*.
(PDF)

Figure S9 **Complementation analysis of deletion mutants transformed with genes 16 and** ***UhAvr1*** **and their virulence toward barley.** **A.** Protein blot analysis of the deletion mutant Uh1041 (Δ*C18A2*) as control (lane 13) and Uh1041 complemented with the full length ORFs of genes 16 and *UhAvr1* with or without their respective signal peptides (-SP) as indicated under the lanes. All genes, lacking a STOP codon, were expressed from the constitutive *U. maydis Hsp70* promoter, attaching the HA epitope tag at the C-terminal end. Cells were grown in liquid medium prior to mating with compatible strain Uh362 for pathogenicity tests and protein was extracted from a sample. Proteins were detected using anti-HA antibody. The relevant sizes in kDa are indicated on the left. **B.** Protein blot analysis of Uh1289 (Δ*UhAvr1*) complemented with *otef*:*UhAvr1:gfp* (lanes 2–4). In strains Uh1353 and Uh1354, *UhAvr1:gfp* is located at the original *UhAvr1* site and has its native promoter; under these conditions it is not expressed (lanes 5 and 6). Lane 1: Uh1351 is Uh364 expressing just GFP from the *otef* promoter and is used as a control. **C.** Results of the pathogenicity tests with the deletion mutant strain Uh1041 and its transformants complemented with each individual gene with C-terminal HA tag as indicated and described in A. Black bars, results on cv. Odessa; red bars, results on cv. Hannchen. The Y-axis indicates the disease incidence as percentage of diseased plants among the total number of inoculated plants. Results of the pathogenicity tests for the GFP-chimers are given in **Table S3**.
(PDF)

Figure S10 **Analysis of virulence towards barley cultivars of a cross of strains both deleted for the C18A2 fragment.** **A.** To obtain a C18A2 deletion strain with mating type 2 (*MAT-2*) for back crossing with mutant strain Uh1041 (*MAT-1* Δ*C18A2*), Uh1041 was first crossed with Uh362 (*MAT-2 Uhavr1*) and teliospores were produced on Hannchen. Random haploid *MAT-2* progeny basidiospores were obtained from germinated teliospores that were carboxin-resistant ensuring the presence of Uh364-inherited chromosome 18. DNA blot analysis of genomic DNA of these progeny confirmed that the proper C18A2 deletion was inherited, presumably replacing the Uh362 chr18. DNA was digested with XhoI and probed with the 3′-flank that was used for construction of the original deletion construct. Three of the deletion mutants that showed a fragment of 5.4 kb (lane 2: Uh1116; lane 3: Uh1117; lane 3: Uh1118) were used for the pathogenicity tests. **B.** Pathogenicity tests of crosses between mating partners both deleted for fragment C18A2 (**Table S2**). Virulence towards both barley cultivars Odessa and Hannchen seemed not significantly different. The Y-axis shows the disease incidence as a percent of infected plants out of total inoculated plants.
(PDF)

Table S1 ***U. hordei*** **genes located on BAC3-A2 (117 kb) and their homologs in** ***U. maydis*** **.**
(PDF)

Table S2 **Strains used in this work.**
(PDF)

Table S3 **Pathogenicity data of** ***U. hordei*** **controls, deletion mutants and complementing transformants.** Results from pathogenicity tests of C18A2 deletion mutant complemented with UHOR_10022 with various C-terminal moieties (HA or GFP) on barley cultivars ‘Odessa’ and ‘Hannchen’.
(PDF)

Table S4 **Primers used in this work.**
(PDF)

Table S5 **Annotated genes in the region of the** ***U. maydis*** **19A cluster.**
(PDF)
