# Supplementary figures and images for: An Immunity-Triggering Effector from the Barley Smut Fungus Ustilago hordei Resides in an Ustilaginaceae-Specific Cluster Bearing Signs of Transposable Element-Assisted Evolution
Source: PLoS Pathog. 2014 Jul 3;10(7):e1004223. doi: 10.1371/journal.ppat.1004223 (PMC4081816; doi:10.1371/journal.ppat.1004223)

**Figure S1.** Ali *et al.*

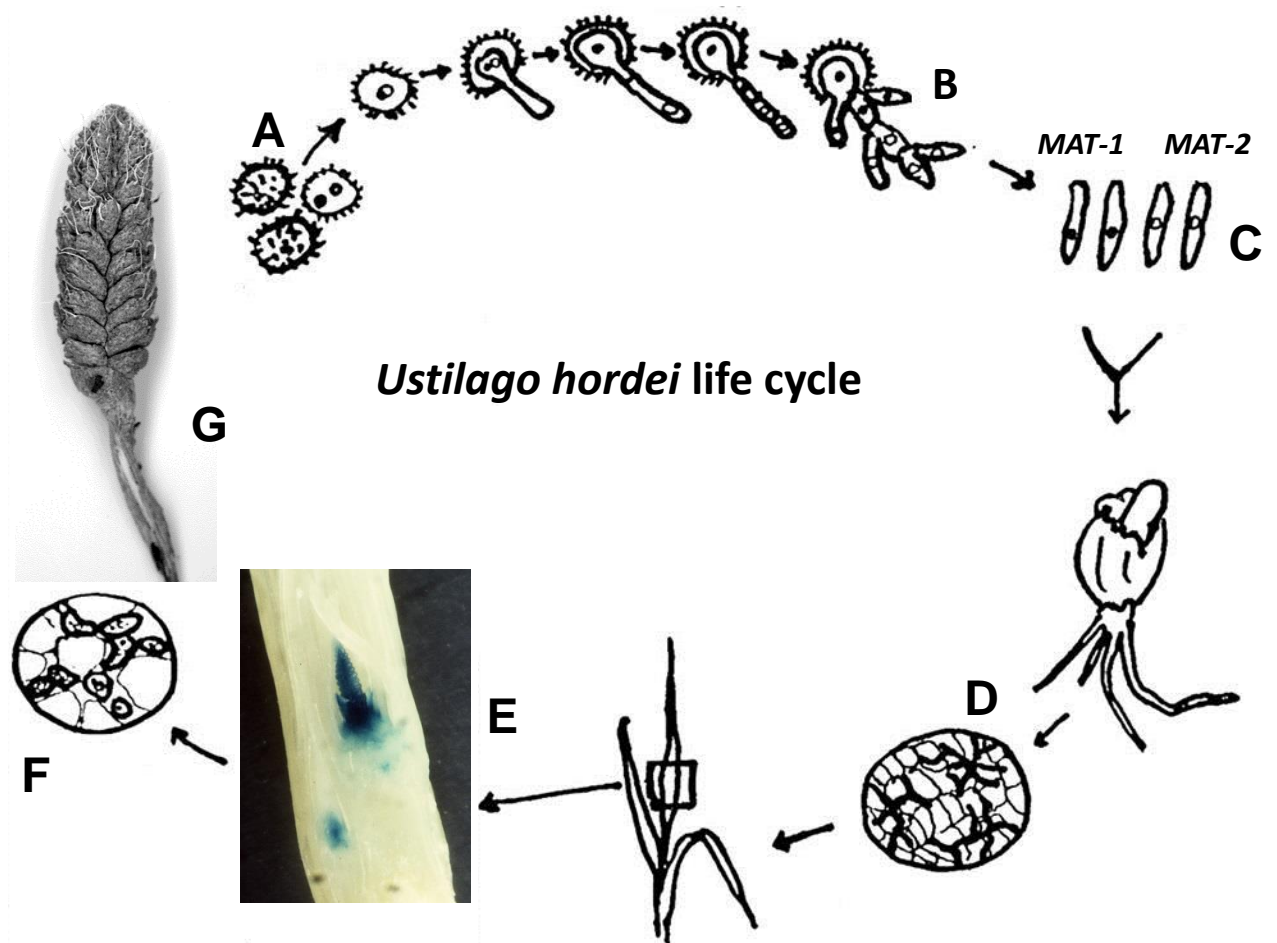

Supplement: Figure S1 — Ustilago hordei life cycle. A. Diploid (2n) teliospores are survival structures. When conditions are right, they germinate, in nature often under the hull of healthy seed that germinate at the same time. B. During teliospore germination, meiosis occurs and four haploid basidiospores are formed on the basidium. C. Mating type segregates 1∶1 (MAT-1 ∶ MAT-2). Cells of opposite mating type can sense each other through the action of pheromones and pheromone receptors upon which each partner forms thin mating hyphae. When mating hyphae meet, fusion takes place whereby the dikaryotic state, characteristic for basidiomycetes, is reconstituted. This fusion also brings together the b mating type gene products from each mating specificity, bW1 and bE2 (and bW2 and bE1) from the respective partner, which form a hetero dimeric protein. This dimer can now regulate transcription of a large number of genes involved in the switch from budding growth to filamentous, pathogenic growth able to infect host tissues. The formed dikaryotic hyphae represent the biotrophic cell type that requires the barley host for completion of the life cycle; haploid cells are saprobic, non-pathogenic and can be manipulated in the lab. Dikaryotic hyphae grow over the surface of the germinating barley coleoptile, led by a cytoplasm-filled growing point and leaving behind septated empty hyphae, until direct penetration through a swelling at the hyphal tip (an appressorium-like structure) leads to infection of epidermal cells. Infection can only occur at early seed germination. D. Hyphae grow inter- and intracellularly, penetrating cell layers to reach the meristematic region of the growing point. Without causing visible symptoms, the fungus only starts proliferating once the barley meristematic region develops into seed spike tissue: E. β-glucuronidase -expressing fungus stained with X-gluc). F. Cells round off and form spore walls. G. Massive sporulation takes place in the developing head where seeds are r [file ppat.1004223.s001.pdf]

Figure S2. Ali *et al.*

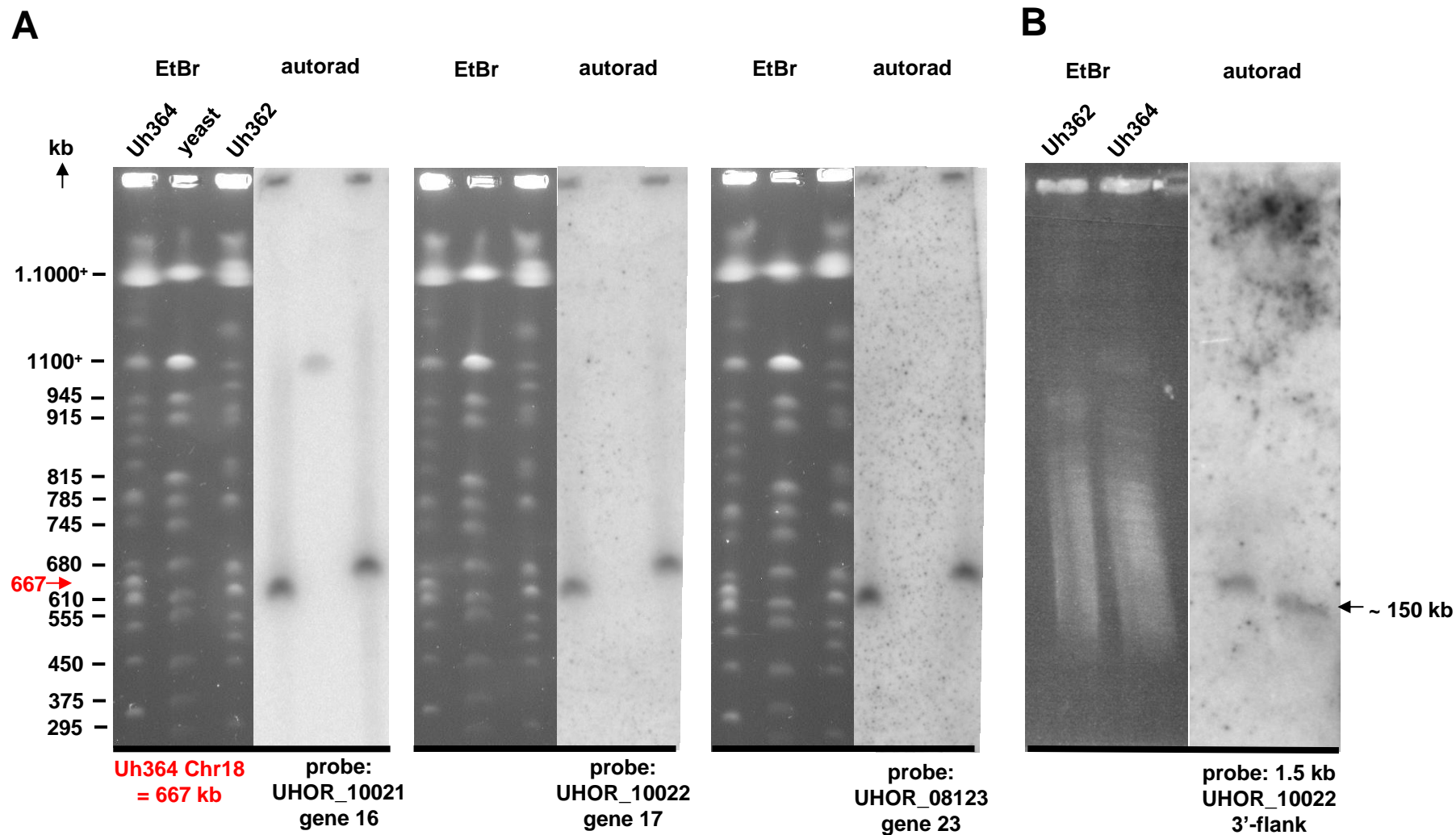

Supplement: Figure S2 — Identification of the U. hordei chromosomes and Not1 fragments harboring UHOR_10022 sequences in strain Uh364, avirulent, and strain Uh362, virulent on cv. Hannchen. A. Chromosomes were separated on a CHEF gel as described [41] with sizes of yeast chromosomes in kilobases (kb) as markers on the left. UhAvr1 has been identified on chromosome 18 (chr18) of an estimated 667 kb in isolate Uh364 [32]. EtBr, Ethidium bromide-stained agarose gel; autorad, DNA blot of the corresponding gel on the left, hybridized to 32P-labeled gene fragments as indicated ( Figure 1 ). Three separate gel panels were used. B. CHEF gel of Not1-digested gDNA fragments revealing a polymorphism by the UhAvr1 gene as a probe. (PDF) [file ppat.1004223.s002.pdf]

**Figure S4.** Ali *et al.*

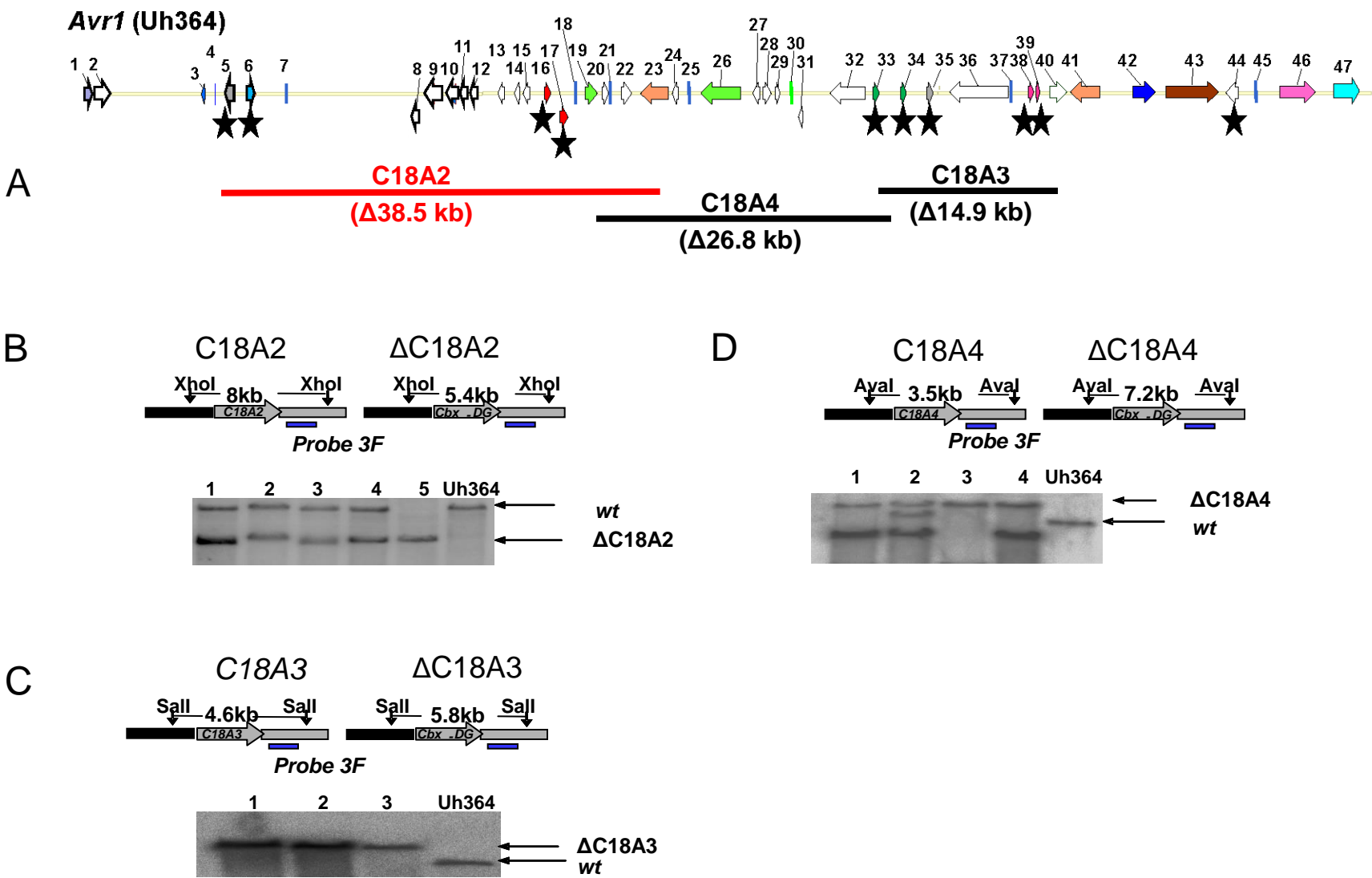

Supplement: Figure S4 — Deletion analysis of the UhAvr1 locus. A. The three overlapping bars (C18A2, C18A4 and C18A3) represent the fragments (with their sizes in kb) that were deleted in the three independent deletion mutants, respectively. The genomic region of the parental avirulent strain Uh364 with all predicted genes is given above (see Figure 1 ). B. DNA blot analysis of genomic DNA of C18A2 transformants, digested with XhoI. One of the transformants, number 5, revealed a band of 5.4 kb expected for the correct deletion mutant and was used for pathogenicity analysis; transformants 1 to 4 revealed both wild-type (wt) and the deletion construct fragments, indicative of an ectopic integration event. C. DNA blot analysis of genomic DNA of C18A3 transformants, digested with SalI. All transformants revealed a fragment of 5.8 kb expected for the correct deletion mutant. D. DNA blot analysis of genomic DNA of C18A4 transformants, digested with AvaI. Transformant 3 revealed a band of the expected size of 7.2 kb to replace the wild-type fragment and was used for pathogenicity analysis. The cartoon above each gel is a schematic representation of the wild-type region in Uh364 (left) and deletion mutant (right). The probe used for each individual analysis was part of the 3′-flanking fragment of the deletion construct used and is indicated as a solid blue line in these cartoons. (PDF) [file ppat.1004223.s004.pdf]

Figure S5. Ali *et al.*

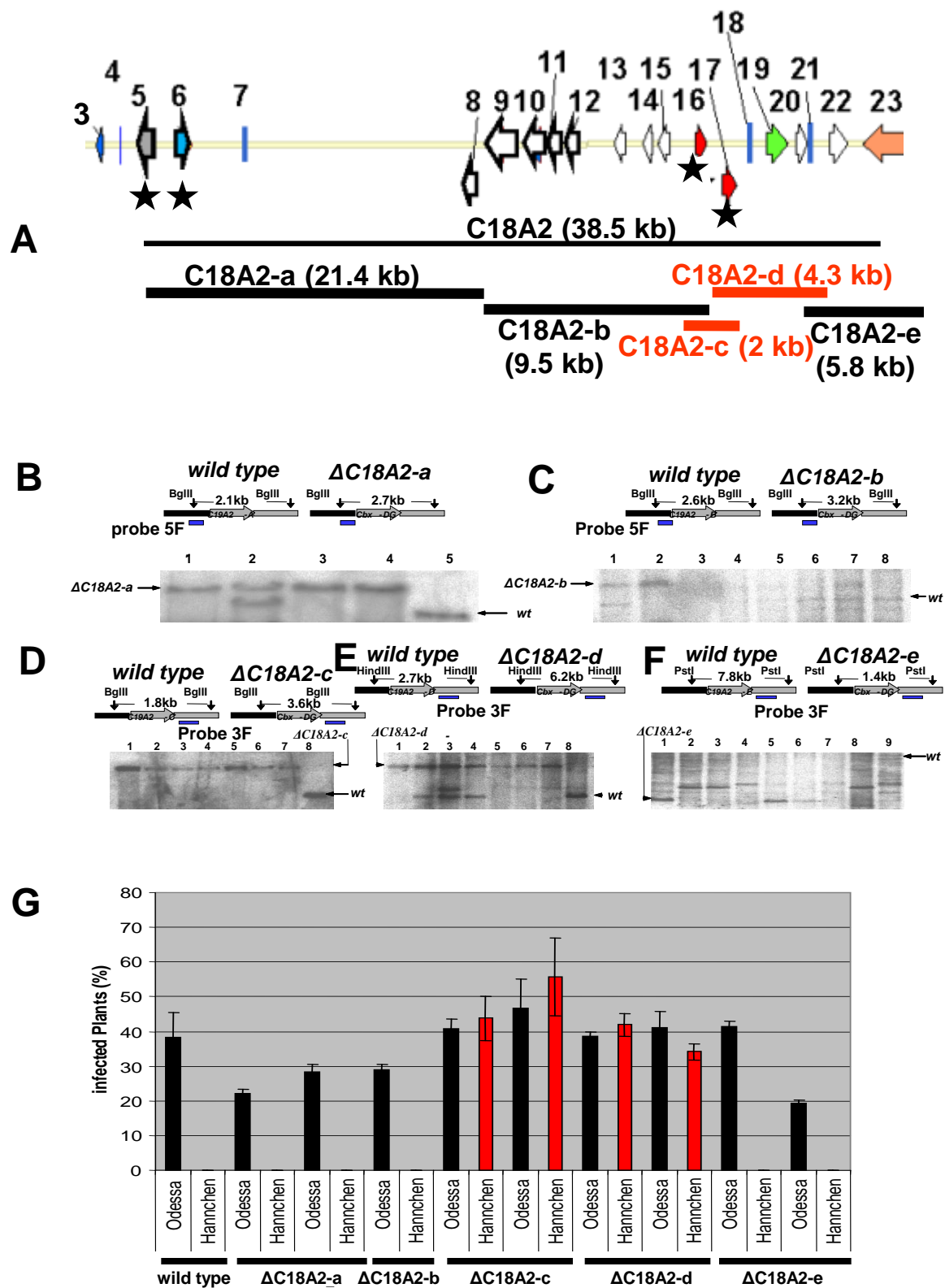

Supplement: Figure S5 — Deletion analysis of fragment C19A2 and pathogenicity tests. A. The five overlapping bars (C18A2-a to e) represent the fragments (with their sizes in kb) that were deleted in the five independent deletion mutants, respectively. The genomic region of the parental avirulent strain Uh364 with all predicted genes is given above (see Figure 1 ). B. DNA blot analysis of genomic DNA of C18A2-a transformants, digested with BglII. Three of the transformants (lanes 1, 3 and 4) show a band of 2.7 kb expected for a proper gene deletion compared to the 2.1 kb fragment present in the wild-type Uh364 strain (wt). C. DNA blot analysis of genomic DNA of C18A2-b transformants, digested with BglII. One of the transformants (lane 2) shows a band of 3.2 kb expected for a proper gene deletion. D. DNA blot analysis of genomic DNA of C18A2-c transformants, digested with BglII. All transformants contained a band of 3.6 kb expected for a proper gene deletion. E. DNA blot analysis of genomic DNA of C18A2-d transformants, digested with HindIII. Four transformants (lanes 1, 5, 6 and 7) show a band of 6.2 kb expected for a proper gene deletion. F. DNA blot analysis of genomic DNA of C18A2-e transformants, digested with PstII. Three transformants (lanes 1, 5 and 6) show a band of 1.4 kb expected for a proper gene deletion. The cartoon above each gel is a schematic representation of the wild-type region in Uh364 (left) and deletion mutant (right). The probes used for each individual analysis were part of the 3′- (3F) or 5′- (5F) flanking fragment of the respective deletion construct used and are indicated as a solid blue line in these cartoons. G. Pathogenicity test of the deletion mutants (two per deletion as indicated on the X-axis). All mutants were crossed with Uh362 (Uhavr1). Mutants deleted for fragments C18A2-c and C18A2-d were virulent towards Hannchen, shown by red bars in the figure, indicating that the functional UhAvr1 gene is located on these fragments. All other deletion mutants and [file ppat.1004223.s005.pdf]

**Figure S6.** Ali *et al.*

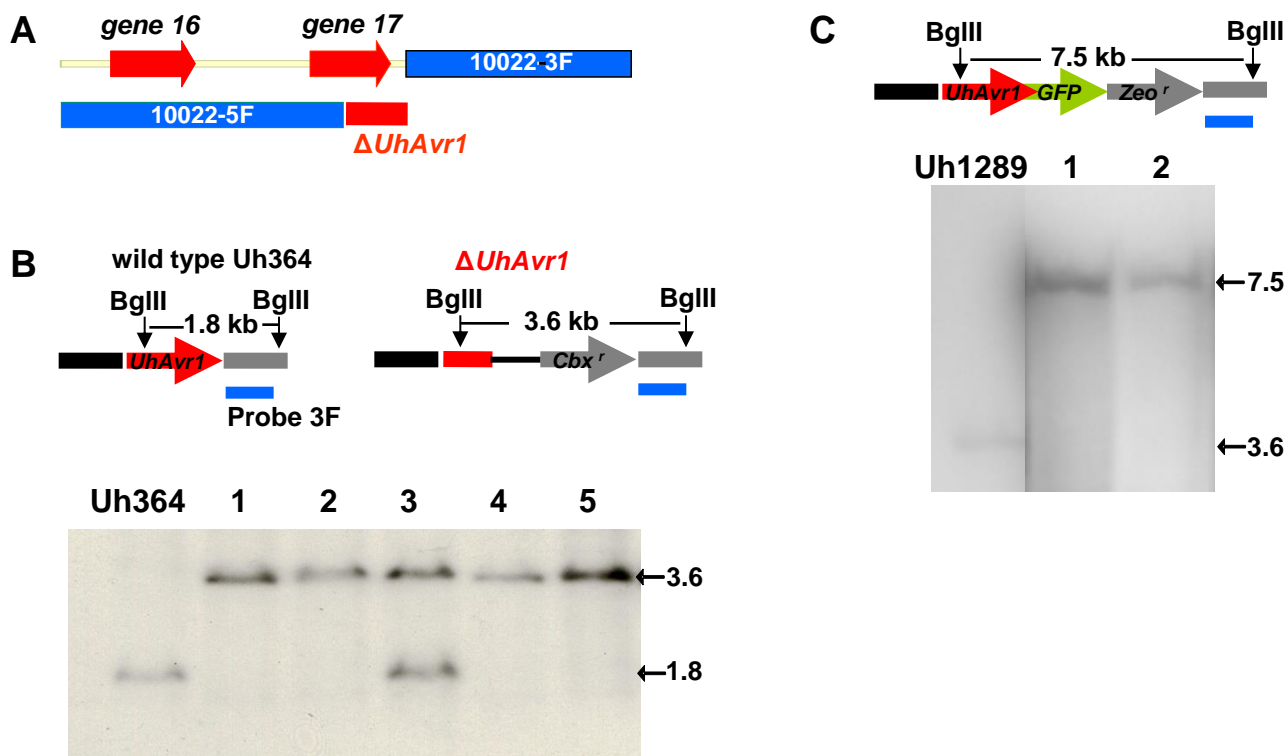

Supplement: Figure S6 — Construction of Δ UhAvr1 and UhAvr1:gfp chimeric replacement mutants. A. Schematic representation of the deletion mutant with the red bar representing the 3′-part of UhAvr1 that was deleted; the blue bars represent the flanks used in the deletion construct. B. Cartoon showing the 3′-end of UhAvr1 replaced by the carboxin resistance gene (Cbxr) and DNA blot analysis of several transformants; total gDNA was digested with BglII and the blue bars indicate the probe. Two of the deletion mutant strains showing a band of expected size of 3.6 kb were used in pathogenicity tests. C. Cartoon showing the replacement construct which reconstitutes the complete UhAvr1 ORF minus STOP codon while linking a GFP moiety to the C-terminus; Cbxr is replaced by zeomycin resistance (Zeor). DNA blot analysis of several transformants; total gDNA was digested with BglII and the blue bar indicates the probe. (PDF) [file ppat.1004223.s006.pdf]

Figure S7. Ali *et al.*

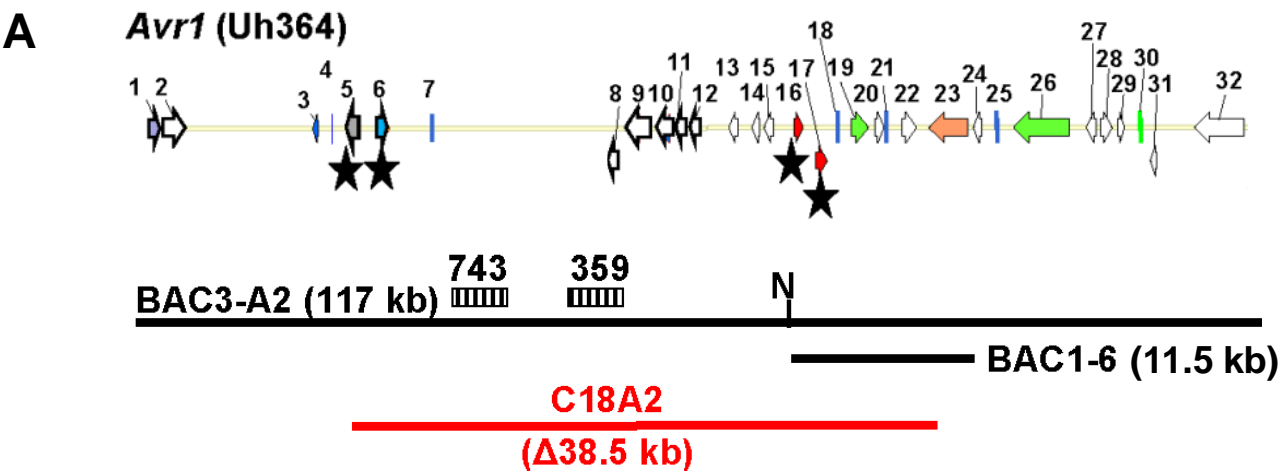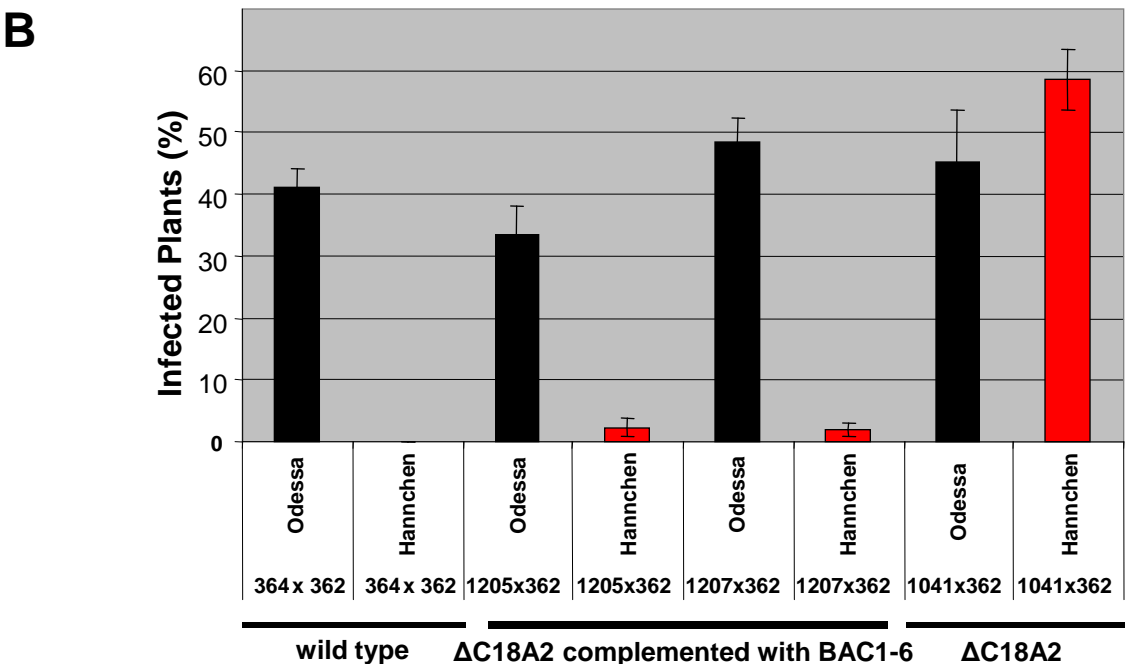

Supplement: Figure S7 — Clone BAC1-6 restores avirulence to the virulent C18A2 deletion mutant. A. The 38.5 kb-fragment C18A2 deleted in the respective mutant Uh1041 is enlarged to show the location of the different ORFs; asterisks indicate the predicted CSEPs (compare Figure 1 ). The position of the complementing 11.5 kb-fragment in BAC1-6 clone is represented by a black line; the overlap contains two predicted CSEPs: gene 16 (UHOR_10021) and gene 17 (UhAvr1). B. Pathogenicity test of the deletion mutant strain (Uh1041) complemented with BAC1-6 as indicated on the X-axis. All mutants were crossed with Uh362 (Uhavr1). While the deletion mutants are fully virulent towards Hannchen, the complemented strains cause very low disease on Hannchen even though they are fully virulent towards Odessa (as a control for infection). This indicates BAC1-6 harbors UhAvr1. The Y-axis shows the percent of infected plants out of the total inoculated plants. The data shown here is an average of three independent experiments with standard deviation as the error bar. (PDF) [file ppat.1004223.s007.pdf]

Figure S9. Ali *et al.*

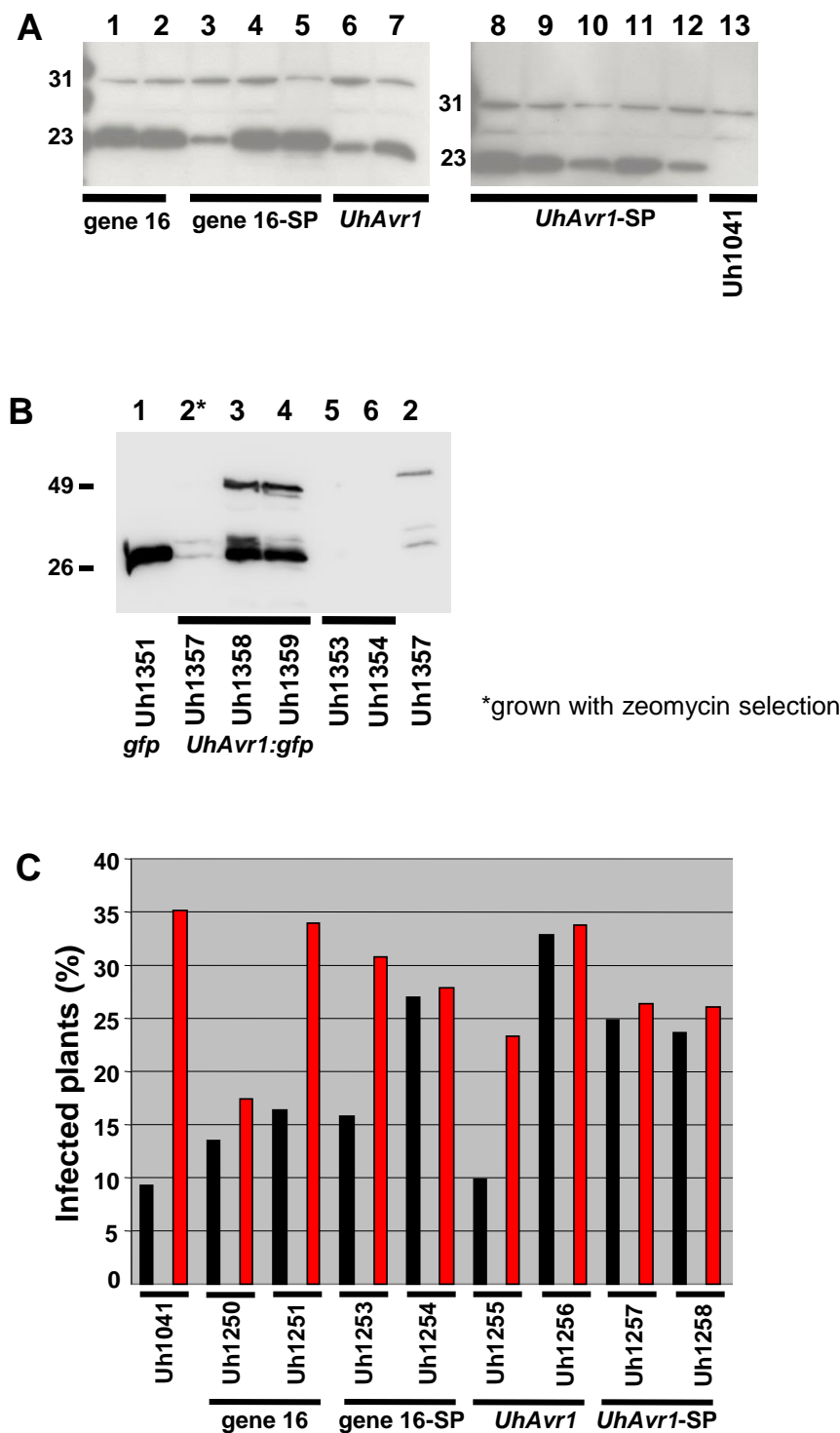

Supplement: Figure S9 — Complementation analysis of deletion mutants transformed with genes 16 and UhAvr1 and their virulence toward barley. A. Protein blot analysis of the deletion mutant Uh1041 (ΔC18A2) as control (lane 13) and Uh1041 complemented with the full length ORFs of genes 16 and UhAvr1 with or without their respective signal peptides (-SP) as indicated under the lanes. All genes, lacking a STOP codon, were expressed from the constitutive U. maydis Hsp70 promoter, attaching the HA epitope tag at the C-terminal end. Cells were grown in liquid medium prior to mating with compatible strain Uh362 for pathogenicity tests and protein was extracted from a sample. Proteins were detected using anti-HA antibody. The relevant sizes in kDa are indicated on the left. B. Protein blot analysis of Uh1289 (ΔUhAvr1) complemented with otef:UhAvr1:gfp (lanes 2–4). In strains Uh1353 and Uh1354, UhAvr1:gfp is located at the original UhAvr1 site and has its native promoter; under these conditions it is not expressed (lanes 5 and 6). Lane 1: Uh1351 is Uh364 expressing just GFP from the otef promoter and is used as a control. C. Results of the pathogenicity tests with the deletion mutant strain Uh1041 and its transformants complemented with each individual gene with C-terminal HA tag as indicated and described in A. Black bars, results on cv. Odessa; red bars, results on cv. Hannchen. The Y-axis indicates the disease incidence as percentage of diseased plants among the total number of inoculated plants. Results of the pathogenicity tests for the GFP-chimers are given in Table S3. (PDF) [file ppat.1004223.s009.pdf]

**Figure S10.** Ali *et al.*

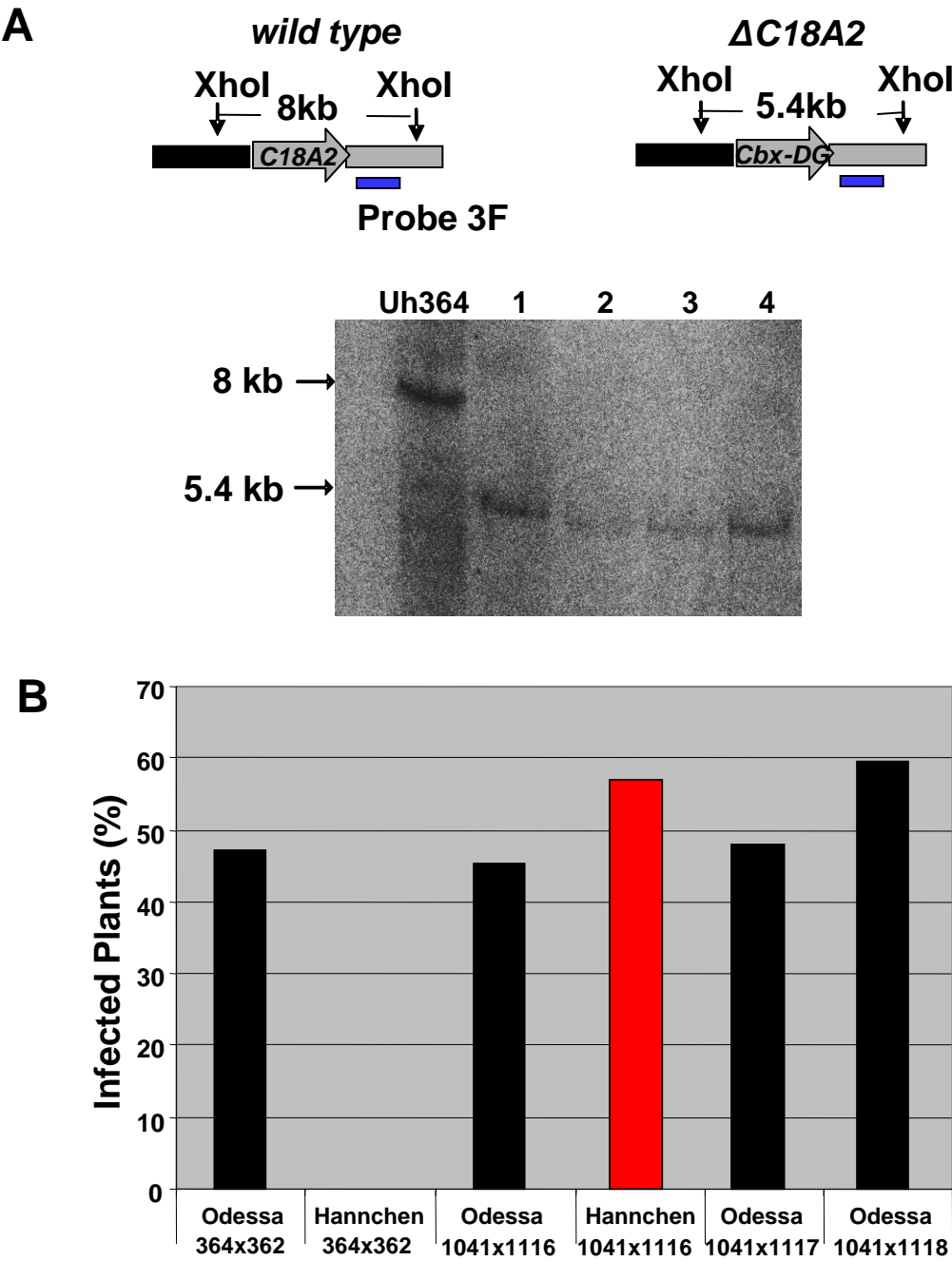

Supplement: Figure S10 — Analysis of virulence towards barley cultivars of a cross of strains both deleted for the C18A2 fragment. A. To obtain a C18A2 deletion strain with mating type 2 (MAT-2) for back crossing with mutant strain Uh1041 (MAT-1 ΔC18A2), Uh1041 was first crossed with Uh362 (MAT-2 Uhavr1) and teliospores were produced on Hannchen. Random haploid MAT-2 progeny basidiospores were obtained from germinated teliospores that were carboxin-resistant ensuring the presence of Uh364-inherited chromosome 18. DNA blot analysis of genomic DNA of these progeny confirmed that the proper C18A2 deletion was inherited, presumably replacing the Uh362 chr18. DNA was digested with XhoI and probed with the 3′-flank that was used for construction of the original deletion construct. Three of the deletion mutants that showed a fragment of 5.4 kb (lane 2: Uh1116; lane 3: Uh1117; lane 3: Uh1118) were used for the pathogenicity tests. B. Pathogenicity tests of crosses between mating partners both deleted for fragment C18A2 (Table S2). Virulence towards both barley cultivars Odessa and Hannchen seemed not significantly different. The Y-axis shows the disease incidence as a percent of infected plants out of total inoculated plants. (PDF) [file ppat.1004223.s010.pdf]
