# Supplementary material for: An Immunity-Triggering Effector from the Barley Smut Fungus Ustilago hordei Resides in an Ustilaginaceae-Specific Cluster Bearing Signs of Transposable Element-Assisted Evolution
Source: PLoS Pathog. 2014 Jul 3;10(7):e1004223. doi: 10.1371/journal.ppat.1004223 (PMC4081816; doi:10.1371/journal.ppat.1004223)
Supplement: Figure S3 — DNA sequence comparisons of alleles from U. hordei isolates from various genes at the UhAvr1 locus. DNA sequence alignments in ClustalX covering: A. gene 16 (UHOR_10021), B. the gene 16-UhAvr1 (UHOR_10022) intergenic region in avirulent strains, C. the gene 16-UhAvr1 intergenic region in virulent strains, D. gene UhAvr1, E. gene 30 (UHOR_08130), F. gene 34 (UHOR_08132), G. gene 38 (UHOR_13916), and H. gene 39 (UHOR_10033), among various isolates. The highlighted asterisks or sequences indicate the ORFs, and the highlighted isolate names indicate an UhAvr1 genotype (see Table S2). (PDF) [file ppat.1004223.s003.pdf]

Figure S3A

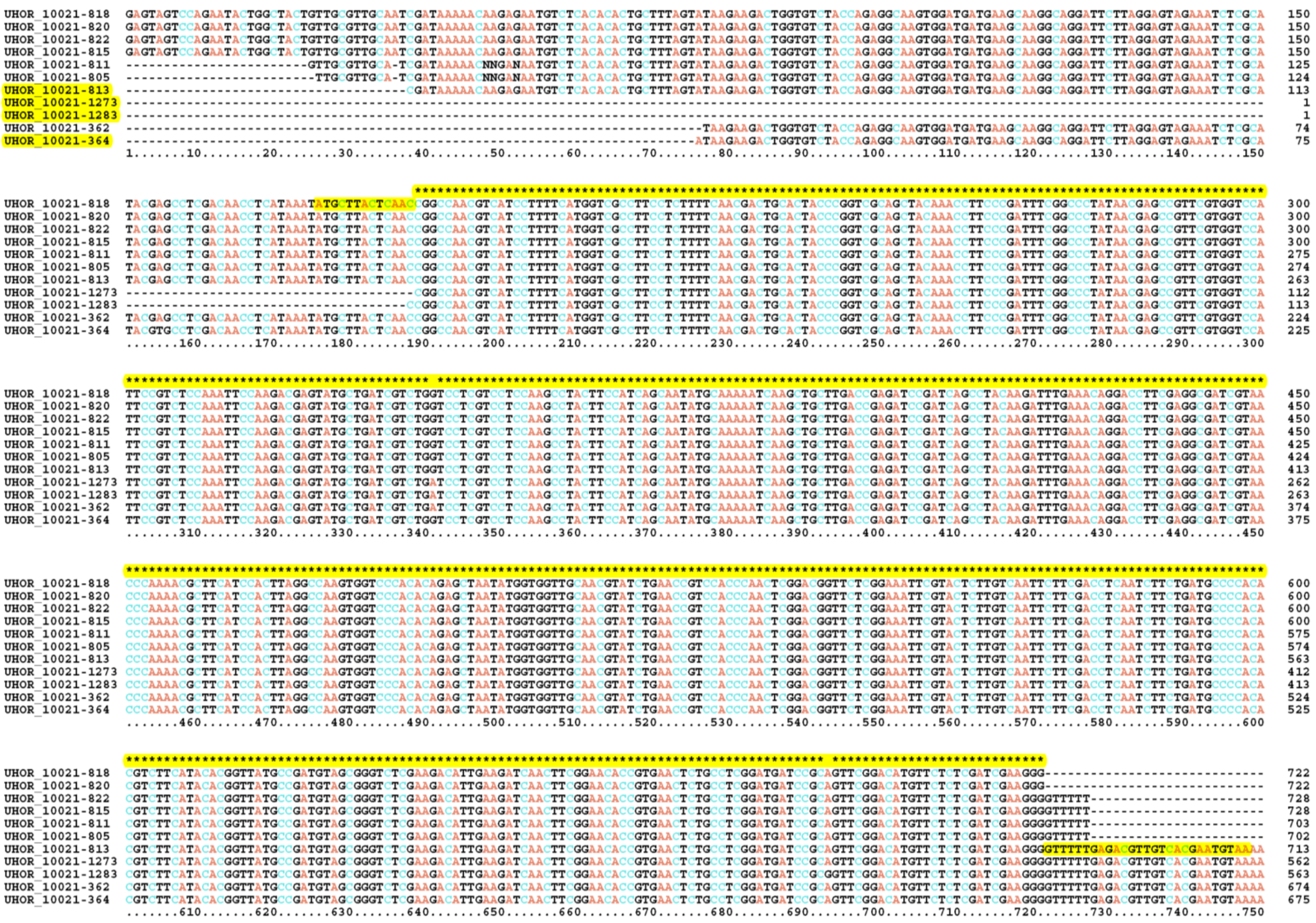

Figure S3A, continued

```
UHOR_10021-818   --------------------------------------------------------------------------------------------------   722
UHOR_10021-820   --------------------------------------------------------------------------------------------------   722
UHOR_10021-822   --------------------------------------------------------------------------------------------------   728
UHOR_10021-815   --------------------------------------------------------------------------------------------------   728
UHOR_10021-811   --------------------------------------------------------------------------------------------------   703
UHOR_10021-805   --------------------------------------------------------------------------------------------------   702
UHOR_10021-813   GCAATGGGTGGTCTCACTGTTCGGATCACGTAACCTAACAACGTTTGTTCAGCCCTGTTACAGTCCAGTGTGTGTGTGAGTGGGTTAATTGAAGATAA   811
UHOR_10021-1273  GCAATGGGTGGTCTCACTGTTCGGATCACGTAACCTAACAACGTTTGTTCAGCCCTGTTACAGTCCAGTGTGTGTGTGAGTGGGTTAATTGAAGATAA   660
UHOR_10021-1283  GCAATGGGTGGTCTCACTGTTCGGATCACGTAACCTAACAACGTTTGTTCAGCCCTGTTACAGTCCAGTGTGTGTGTGAGTGGGTTAATTGAAGATAA   661
UHOR_10021-362   GCAATGGGTGGTCTCACTGTTCGGATCACGTAACCTAACAACGTTTGTTCAGCCCTGTTACAGTCCAGTGTGTGTGTGAGTGGGTTGCTCAGACTGAA   772
UHOR_10021-364   GCAATGGGTGGTCTCACTGTTCGGATCACGTAACCTAACAACGTTTGTTCAGCCCTGTTACAGTCCAGTGTGTGTGTGAGTGGGTTGCTCAGACTGAA   773
                 .......760.......770.......780.......790.......800.......810.......820.......830.......840........
```

Figure S3B

UHOR_10021 – UHOR_10022 intergenic region

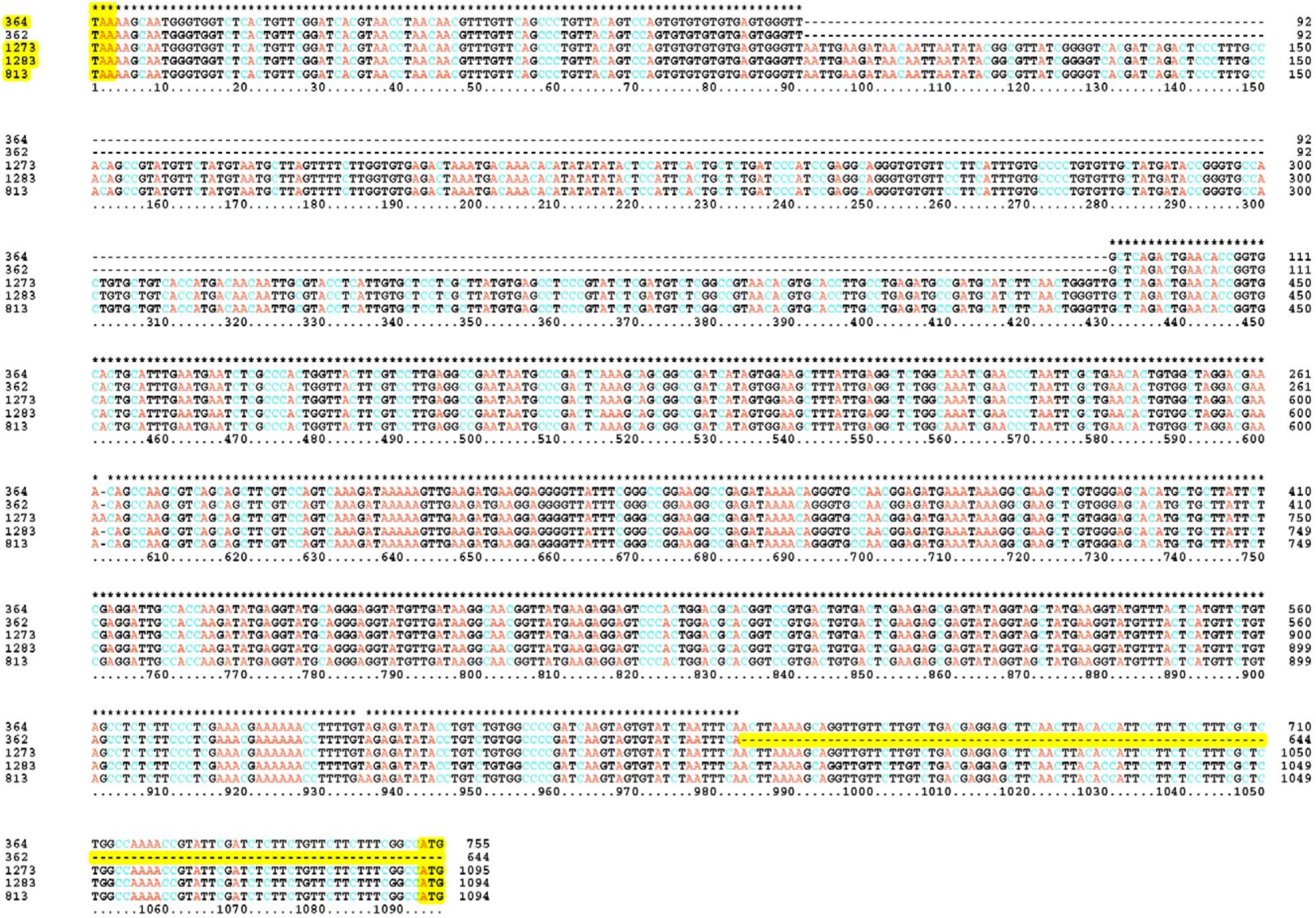

Figure S3C

UHOR_10021 – *Uhavr1* locus region in virulent isolates

```
                                             983
Uh364brk    CAAGTAGTGTATCTAATTTCA----------------------------------------------------------------------------
Uh822       CAAGTAGTGTATCTAATTTCAGTTGAAGTCATTCGTCAGCGTCTTGGCGTCATTCGTCAGAGNTTGGGGTTGAGGTGTCTTTTGTTGATGATGGTAG
Uh811       CAAGTAGTGTATCTAATTTCAGTTGAAGTCATTCGTCAGCGTCTTGGCGTCATTCGTCAGAGGTTGGGGTTGAGGTGTCTTTTGTTGATGATGGTAG
Uh818       CAAGTAGTGTATCTAATTTCAGTTGAAGTCATTCGTCAGCGTCTTGGCGTCATTCGTCAGAGGTTGGGGTTGAGGTGTCTTTTGTTGATGATGGTAG
Uh815       CAAGTAGTGTATCTAATTTCAGTTGAAGTCATTCGTCAGCGTCTTGGCGTCATTCGTCAGAGGTTGGGGTTGAGGTGTCTTTTGTTGATGATGGTAG
Uh805       CAAGTAGTGTATCTAATTTCAGTTGAAGTCATTCGTCAGCGTCTTGGCGTCATTCGTCAGAGGTTGGGGTTGAGGTGTCTTTTGTTGATGATGGTAG
Uh362       CAAGTAGTGTATCTAATTTCAGTTGAAGTCATTCGTCAGCGTCTTGGCGTCATTCGTCAGAGGTTGGGGTTGAGGTGTCTTTTGTTGATGATGGTAG
Uh820       CAAGTAGTGTATGTAATTTCAGTTGAAGTCATTCGTCAGCGTCTNGGNGTCATTCGTCAGAGGTTGGGGNTGAGGTGTCTTTTGTTGATGATGGTAG
            ************ ********

Uh364brk    ---------------------------------------------------------------------------------------------------
Uh822       GAGAGAGAGAGAGC----------TAGGAGTCTATATATACAAAAGTAGAGCGGCGGACTGCGGAGCATAACAAAGCATTTGCCTTGAGTAGGTTGTGA
Uh811       GAGAGAGAGAGAGC----------TAGGAGTCTATATATACAAAAGTAGAGCGGCGGACTGCGGAGCATAACAAAGCATTTGCCTTGAGTAGGTTGTGA
Uh818       GAGAGAGAGAGAGC----------TAGGAGTCTATATATACAAAAGTAGAGCGGCGGACTGCGGAGCATAACAAAGCATTTGCCTTGAGTAGGTTGTGA
Uh815       GAGAGAGAGAGAGCGAGAGAGAGCTAGGAGTCTATATATACAAAAGTAGAGCGGCGGACTGCGGAGCATAACAAAGCATTTGCCTTGAGTAGGTTGTGA
Uh805       GAGAGAGAGAGAGCGAGAGAGAGCTAGGAGTCTATATATACAAAAGTAGAGCGGCGGACTGCGGAGCATAACAAAGCATTTGCCTTGAGTAGGTTGTGA
Uh362       GAGAGAGAGAGAGCGAGAGAGAGCTAGGAGTCTATATATACAAAAGTAGAGCGGCGGACTGCGGAGCATAACAAAGCATTTGCCTTGAGTAGGTTGTGA
Uh820       GAGAGAGAGAGAGCGAGAGAGAGCTAGGAGTCTATATATACAAAAGTAGAGCGGCGGACTGCGGAGCATAACAAAGCATTTGCCTTGAGTAGGTTGTGA
```

Comparison of *Uhavr1* locus sequences among seven virulent isolates, focusing on the region after the 'breakpoint' where Uh362 (grey) diverges from sequences in avirulent isolate Uh364 (brk, highlighted in yellow). The 10-bp repeat unit is in red. The base position is as in panel B.

Figure S3D

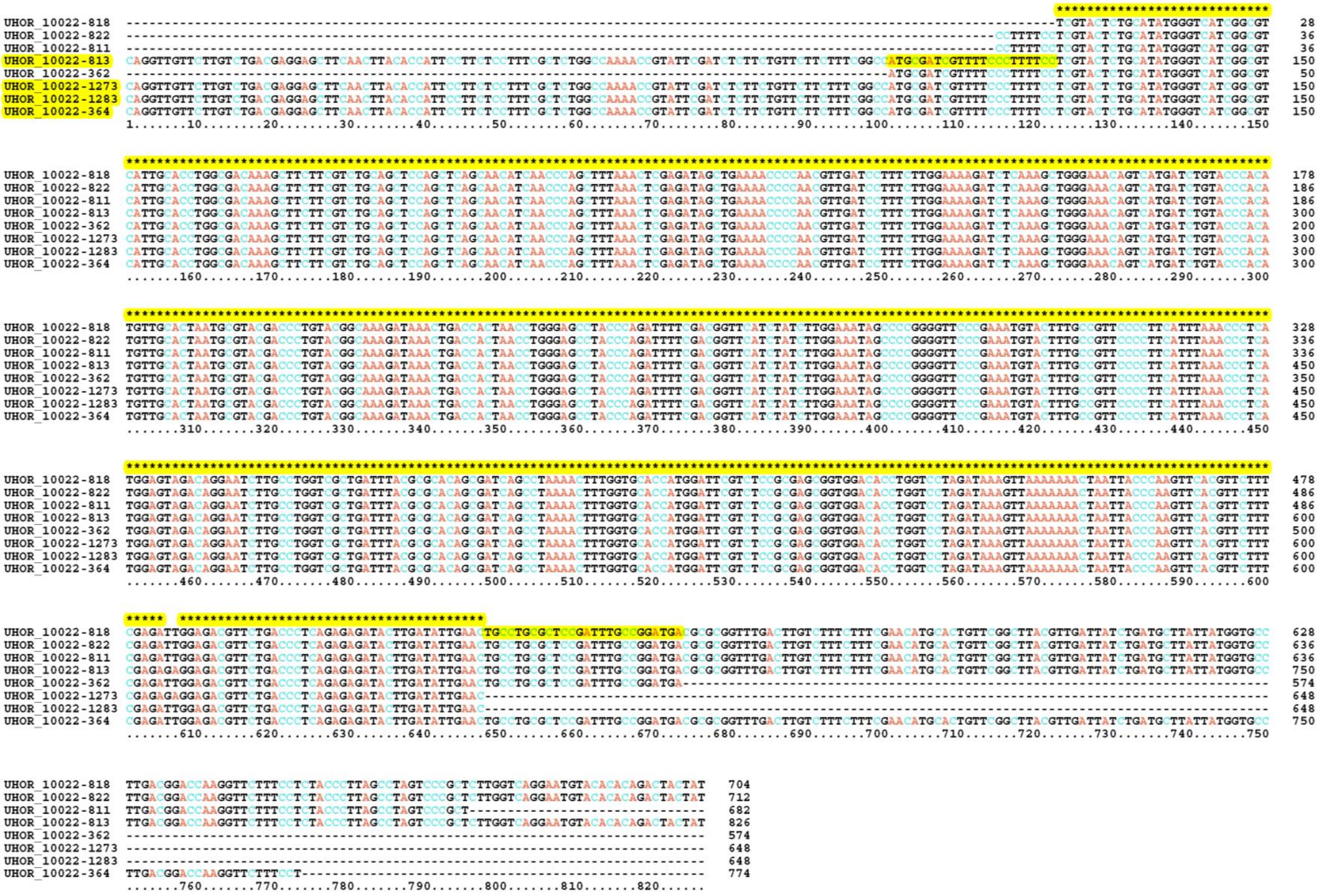
UHOR_10022-818
UHOR_10022-822
UHOR_10022-811
UHOR_10022-813
UHOR_10022-362
UHOR_10022-1273
UHOR_10022-1283
UHOR_10022-364

Figure S3E

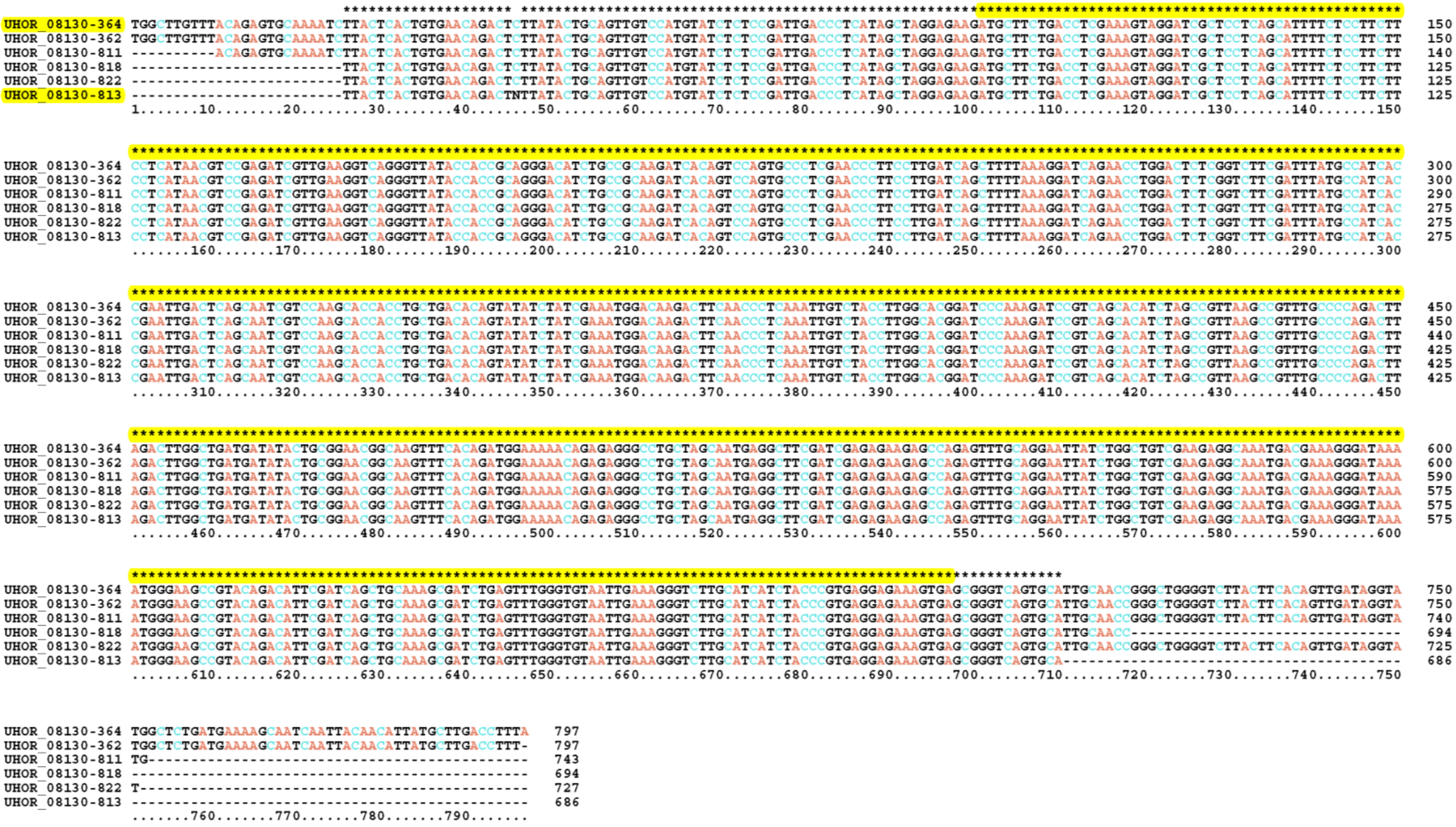
UHOR_08130-364
UHOR_08130-362
UHOR_08130-811
UHOR_08130-818
UHOR_08130-822
UHOR_08130-813

Figure S3F

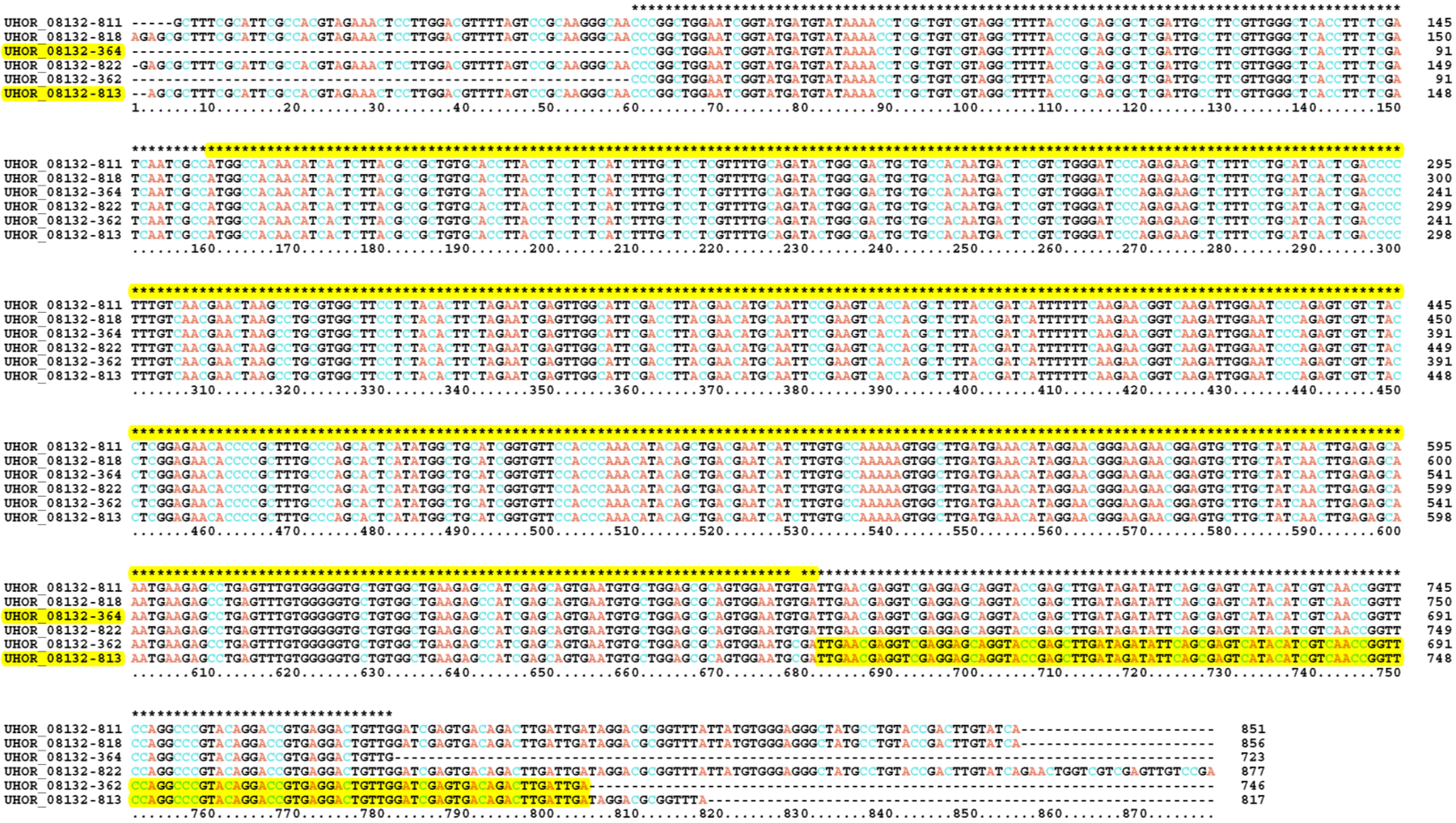
UHOR_08132-811
UHOR_08132-818
UHOR_08132-364
UHOR_08132-822
UHOR_08132-362
UHOR_08132-813

Figure S3G

```
                                                                                              ***********************************************************************
UHOR_13916-364 -------------------------------------------------------------------------------TTTTTAGAGGTGTGTAGGCGAGGCTGGTGCGGACCTGTGATAAGGGGTAACGACGTAGACGATCTTCCCCC   71
UHOR_13916-362 --------------------------------------------------------------------------------TTTTAGAGGTGTGTAGGCGAGGCTGGTGCGGACCTGTGATAAGGGGTAACGACGTAGACGATCTTCCCCC   70
UHOR_13916-811 -----------------------------------AGGTATACGTCATGCGATCATGTTGGAGCGGTATAAACATCTGATTTTTAGAGGTGTGTAGGCGAGGCTGGTGCGGACCTGTGATAAGGGGTAACGACGTAGACGATCTTCCCCC  115
UHOR_13916-813 ----------------------------------------TACGTCATGCGATCATGTTGGAGCGGTATAAACATCTGATTTTTAGAGGTGTGTAGGCGAGGCTGGTGCGGACCTGTGATAAGGGGTAACGACGTAGACGATCTTCCCCC  110
UHOR_13916-822 ----------------------------------------TACGTCATGCGATCATGTTGGAGCGGTATAAACATCTGATTTTTAGAGGTGTGTAGGCGAGGCTGGTGCGGACCTGTGATAAGGGGTAACGACGTAGACGATCTTCCCCC  110
UHOR_13916-818 CCAACAGGGGTTTTGTGGGAGTGATTGAGAAAGGAAGGTATACGTCATGCGATCATGTTGGAGCGGTATAAACATCTGATTTTTAGAGGTGTGTAGGCGAGGCTGGTGCGGACCTGTGATAAGGGGTAACGACGTAGACGATCTTCCCCC  150
               1.......10........20........30........40........50........60........70........80........90.......100.......110.......120.......130.......140.......150

               ******************************************************************************************************************************************************
UHOR_13916-364 CACCCCCCTATCTGGCTCTCTTCTCCAATATGACCCGTCTCAAGCAAAACATTGCCTCTTTCATCACTCACCTTCCCATCCTCCTCCTCCTCCTCGCCACTTCCTACACCTCTCGATTCACTCTCATACCCGAGAGAGGTAACCCTCACG  221
UHOR_13916-362 CACCCCCCTATCTGGCTCTCTTCTCCAATATGACCCGTCTCAAGCAAAACATTGCCTCTTTCATCACTCACCTTCCCATCCTCCTCCTCCTCCTCGCCACTTCCTACACCTCTCGATTCACTCTCATACCCGAGAGAGGTAACCCTCACG  220
UHOR_13916-811 CACCCCCCTATCTGGCTCTCTTCTCCAATATGACCCGTCTCAAGCAAAACATTGCCTCTTTCATCACTCACCTTCCCATCCTCCTCCTCCTCCTCGCCACTTCCTACACCTCTCGATTCACTCTCATACCCGAGAGAGGTAACCCTCACG  265
UHOR_13916-813 CACCCCCCTATCTGGCTCTCTTCTCCAATATGACCCGTCTCAAGCAAAACATTGCCTCTTTCATCACTCACCTTCCCATCCTCCTCCTCCTCCTCGCCACTTCCTACACCTCTCGATTCACTCTCATACCCGAGAGAGGTAACCCTCACG  260
UHOR_13916-822 CACCCCCCTATCTGGCTCTCTTCTCCAATATGACCCGTCTCAAGCAAAACATTGCCTCTTTCATCACTCACCTTCCCATCCTCCTCCTCCTCCTCGCCACTTCCTACACCTCTCGATTCACTCTCATACCCGAGAGAGGTAACCCTCACG  260
UHOR_13916-818 CACCCCCCTATCTGGCTCTCTTCTCCAATATGACCCGTCTCAAGCAAAACATTGCCTCTTTCATCACTCACCTTCCCATCCTCCTCCTCCTCCTCGCCACTTCCTACACCTCTCGATTCACTCTCATACCCGAGAGAGGTAACCCTCACG  300
               .......160.......170.......180.......190.......200.......210.......220.......230.......240.......250.......260.......270.......280.......290.......300

               ******************************************************************************************************************************************************
UHOR_13916-364 GCGCACTAGCCTTCTCCACCTTCAATTTTCTACACGAACGCACCGGGAAATGGAAACATGCGGTTACGGCACAGTACAGTACTAAGTATTACCCCTGGACTACGAGCCGAAGTGTTGGGTGAAAGGCATCTGACGGAGGATTTGGTAGGG  371
UHOR_13916-362 GCGCACTAGCCTTCTCCACCTTCAATTTTCTACACGAACGCACCGGGAAATGGAAACATGCGGTTACGGCACAGTACAGTACTAAGTATTACCCCTGGACTACGAGCCGAAGTGTTGGGTGAAAGGCATCTGACGGAGGATTTGGTAGGG  370
UHOR_13916-811 GCGCACTAGCCTTCTCCACCTTCAATTTTCTACACGAACGCACCGGGAAATGGAAACATGCGGTTACGGCACAGTACAGTACTAAGTATTACCCCTGGACTACGAGCCGAAGTGTTGGGTGAAAGGCATCTGACGGAGGATTTGGTAGGG  415
UHOR_13916-813 GCGCACTAGCCTTCTCCACCTTCAATTTTCTACACGAACGCACCGGGAAATGGAAACATGCGGTTACGGCACAGTACAGTACTAAGTATTACCCCTGGACTACGAGCCGAAGTGTTGGGTGAAAGGCATCTGACGGAGGATTTGGTAGGG  410
UHOR_13916-822 GCGCACTAGCCTTCTCCACCTTCAATTTTCTACACGAACGCACCGGGAAATGGAAACATGCGGTTACGGCACAGTACAGTACTAAGTATTACCCCTGGACTACGAGCCGAAGTGTTGGGTGAAAGGCATCTGACGGAGGATTTGGTAGGG  410
UHOR_13916-818 GCGCACTAGCCTTCTCCACCTTCAATTTTCTACACGAACGCACCGGGAAATGGAAACATGCGGTTACGGCACAGTACAGTACTAAGTATTACCCCTGGACTACGAGCCGAAGTGTTGGGTGAAAGGCATCTGACGGAGGATTTGGTAGGG  450
               .......310.......320.......330.......340.......350.......360.......370.......380.......390.......400.......410.......420.......430.......440.......450

               *************************************************************************
UHOR_13916-364 AGGGAATTACACGAGGTACCGAAGAAGTTGCACAAGAAAGCGATTTGGAATCAAGGAGAGATAGCGGGGTTT------------------------------------------------------------------------------  444
UHOR_13916-362 AGGGAATTACACGAGGTACCGAAGAAGTTGCACAAGAAAGCGATTTGGAATCAAGGAGAGATAGCGGGGTTT------------------------------------------------------------------------------  443
UHOR_13916-811 AGGGAATTACACGAGGTACCGAAGAAGTTGCACAAGAAAGCGATTTGGAATCAAGGAGAGATAGCGGGGTTTTTTTGGTAGCTCTGCGGTTAGGATGTGAGGTGAGGGGTCACGTGGAAGGAGAAATGGGTAGCGCTGAGTGGTCGACGC  565
UHOR_13916-813 AGGGAATTACACGAGGTACCGAAGAAGTTGCACAAGAAAGCGATTTGGAATCAAGGAGAGATAGCGGGGTTTTTTTGGTAGCTCTGCGGTTAGGATGTGAGGTGAGGGGTCACGTGGAAGGAGAAATGGGTAGCGCTGAGTGGTCGACGC  560
UHOR_13916-822 AGGGAATTACACGAGGTACCGAAGAAGTTGCACAAGAAAGCGATTTGGAATCAAGGAGAGATAGCGGGGTTTTTTTGGTAGCTCTGCGGTTAGGATGTGAGGTGAGGGGTCACGTGGAAGGAGAAATGGGTAGCGCTGAGTGGTCGACGC  560
UHOR_13916-818 AGGGAATTACACGAGGTACCGAAGAAGTTGCACAAGAAAGCGATTTGGAATCAAGGAGAGATAGCGGGGTTTTTTTGGTAGCTCTGCGGTTAGGATGTGAGGTGAGGGGTCACGTGGAAGGAGAAATGGGTAGCGCTGAGTGGTCGACGC  600
               .......460.......470.......480.......490.......500.......510.......520.......530.......540.......550.......560.......570.......580.......590.......600

UHOR_13916-364 ---------------------------------------------------------------------------------------------------------------------------------------------  444
UHOR_13916-362 ---------------------------------------------------------------------------------------------------------------------------------------------  443
UHOR_13916-811 TGCGAATGAAAAGGGGGAATCGGAAAGTTCGGTAAGAAGTTCCGTTGGGTGATGAGGGTGGAGGTTAATGATGATGTCGGATGTGGCTTTATACGATCAATTCAAAGCTGGAGGTATGGTCACGATGGGTTTACCGTGGCT  706
UHOR_13916-813 TGCGAATGAAAAGGGGGAATCGGAAAGTTCGGTAAGAAGTTCCGTTGGGTGATGAGGGTGGAGGTTAATGATGATGTCGGATGTGGCTTTATACGATCAATTCAAAGCTGGAGGTATGGTCACGATGGGTTTACCGTGGCT  701
UHOR_13916-822 TGCGAATGAAAAGGGGGAATCGGAAAGTTCGGTAAGAAGTTCCGTTGGGTGATGAGGGTGGAGGTTAATGATGATGTCGGATGTGGCTTTATACGATCAATTCAAA-----------------------------------  667
UHOR_13916-818 TGCGAATGAAAAGGGGGAATCGGAAAGTTCGGTAAGAAGTTCCGTTGGGTGATGAGGGTGGAGGTTAATGATGATGTCGGATGTGGCTTTATACGATCAATTCAAAGCTGGAGGTATGGTCACGATGGGTTTACCGTGGCT  741
               .......610.......620.......630.......640.......650.......660.......670.......680.......690.......700.......710.......720.......730.......740.
```

Figure S3H

```
UHOR_10033-813 --AAGCTGGAGGTATGGTCACGATGGGTTTACCGTGGCTTAAAAGANGGAAGTGACGGAAGGGACCCTGATACGCAGCACCATCCGTGATGAGAGCCCTTGAGCTTGACTTGAATGATGGGCGAGCGAGGCTCCGTGTCAATCGTGTTGC   148
UHOR_10033-822 CAAAGCTGGAGGTATGGTCACGATGGGTTTACCGTGGCTTAAAAGATGGAAGTGACGGAAGGGACCCTGATACGCAGCACCATCCGTGATGAGAGCCCTTGAGCTTGACTTGAATGATGGGCGAGCGAGGCTCCGTGTCAATCGTGTTGC   150
UHOR_10033-818 CAAAGCTGGAGGTATGGTCACGATGGGTTTACCGTGGCTTAAAAGATGGAAGTGACGGAAGGGACCCTGATACGCAGCACCATCCGTGATGAGAGCCCTTGAGCTTGACTTGAATGATGGGCGAGCGAGGCTCCGTGTCAATCGTGTTGC   150
UHOR_10033-811 ------------------------------------------------------------------------------------------------------------------------------------------------------     1
UHOR_10033-364 ------------------------------------------------------------------------------------------------CCTTGAGCTTGACTTGAATGATGGGCGAGCGAGGCTCCGTGTCAATCGTGTTGC    54
UHOR_10033-362 -------------------------------------------------------------------------------------------------CTTGAGCTTGACTTGAATGATGGGCGAGCGAGGCTCCGTGTCAATCGTGTTGC    53
               1.......10........20........30........40........50........60........70........80........90.......100.......110.......120.......130.......140.......150

                                                             ********************************************************************************************************
UHOR_10033-813 TCTACTTATCTCAAACTCAAGCTCACAAACTGACACAACAATCACGATGCGCTTCGCATCCCTCGTCATCCTCCTTACTCTCCTCTCCACTGCCGCTCTCGCATCCATGGAGCTGGAAGAACCAGAATCAGAACCACACCCCGACATCCT   298
UHOR_10033-822 TCTACTTATCTCAAACTCAAGCTCACAAACTGACACAACAATCACGATGCGCTTCGCATCCCTCGTCATCCTCCTTACTCTCCTCTCCACTGCCGCTCTCGCATCCATGGAGCTGGAAGAACCAGAATCAGAACCACACCCCGACATCCT   300
UHOR_10033-818 TCTACTTATCTCAAACTCAAGCTCACAAACTGACACAACAATCACGATGCGCTTCGCATCCCTCGTCATCCTCCTTACTCTCCTCTCCACTGCCGCTCTCGCATCCATGGAGCTGGAAGAACCAGAATCAGAACCACACCCCGACATCCT   300
UHOR_10033-811 ----------------------------------------------ATGCGCTTCGCATCCCTCGTCATCCTCCTTACTCTCCTCTCCACTGCCGCTCTCGCATCCATGGAGCTGGAAGAACCAGAATCAGAACCACACCCCGACATCCT   104
UHOR_10033-364 TCTACTTATCTCAAACTCAAGCTCACAAACTGACACAACAATCACGATGCGCTTCGCATCCCTCGTCATCCTCCTTACTCTCCTCTCCACTGCCGCTCTCGCATCCATGGAGCTGGAAGAACCAGAATCAGAACCACACCCCGACATCCT   204
UHOR_10033-362 TCTACTTATCTCAAACTCAAGCTCACAAACTGACACAACAATCACGATGCGCTTCGCATCCCTCGTCATCCTCCTTACTCTCCTCTCCACTGCCGCTCTCGCATCCATGGAGCTGGAAGAACCAGAATCAGAACCACACCCCGACATCCT   203
               .......160.......170.......180.......190.......200.......210.......220.......230.......240.......250.......260.......270.......280.......290.......300

               ******************************************************************************************************************************************************
UHOR_10033-813 TCACTCCATCTCTTCCAACTCCTTCGCTCAACACCACGCTCGATCCGTCATCTCCTACCTTTCACTTCCCTCTTCCACCATCACCCGTGTAGACCCACCCGATTACAGACCCGACCTTCGCTTCCTTGTCCATGACAACACCGACAGCTC   448
UHOR_10033-822 TCACTCCATCTCTTCCAACTCCTTCGCTCAACACCACGCTCGATCCGTCATCTCCTACCTTTCACTTCCCTCTTCCACCATCACCCGTGTAGACCCACCCGATTACAGACCCGACCTTCGCTTCCTTGTCCATGACAACACCGACAGCTC   450
UHOR_10033-818 TCACTCCATCTCTTCCAACTCCTTCGCTCAACACCACGCTCGATCCGTCATCTCCTACCTTTCACTTCCCTCTTCCACCATCACCCGTGTAGACCCACCCGATTACAGACCCGACCTTCGCTTCCTTGTCCATGACAACACCGACAGCTC   450
UHOR_10033-811 TCACTCCATCTCTTCCAACTCCTTCGCTCAACACCACGCTCGATCCGTCATCTCCTACCTTTCACTTCCCTCTTCCACCATCACCCGTGTAGACCCACCCGATTACAGACCCGACCTTCGCTTCCTTGTCCATGACAACACCGACAGCTC   254
UHOR_10033-364 TCACTCCATCTCTTCCAACTCCTTCGCTCAACACCACGCTCGATCCGTCATCTCCTACCTTTCACTTCCCTCTTCCACCATCACCCGTGTAGACCCACCCGATTACAGACCCGACCTTCGCTTCCTTGTCCATGACAACACCGACAGCTC   354
UHOR_10033-362 TCACTCCATCTCTTCCAACTCCTTCGCTCAACACCACGCTCGATCCGTCATCTCCTACCTTTCACTTCCCTCTTCCACCATCACCCGTGTAGACCCACCCGATTACAGACCCGACCTTCGCTTCCTTGTCCATGACAACACCGACAGCTC   353
               .......310.......320.......330.......340.......350.......360.......370.......380.......390.......400.......410.......420.......430.......440.......450

               *****************************************************************
UHOR_10033-813 TCGAAACGTCCCTTTGTGCTATCGTGACGAACACTGTCTGATGGTCTCTCTGTACGAACTCTGACAGTATAATAGAAGAGGTGAGATGGAAAAGAAGGCGAAAGTGTTGATGACCAGG--------------------------------   567
UHOR_10033-822 TCGAAACGTCCCTTTGTGCTATCGTGACGAACACTGTCTGATGGTCTCTCTGTACGAACTCTGACAGTATAATAGAAGAGGTGAGATGGAAAAGAAGGCGAAAGTGTTGATGACCAGGTT------------------------------   571
UHOR_10033-818 TCGAAACGTCCCTTTGTGCTATCGTGACGAACACTGTCTGATGGTCTCTCTGTACGAACTCTGACAGTATAATAGAAGAGGTGAGATGGAAAAGAAGGCGAAAGTGTTGATGACCAGGTT------------------------------   571
UHOR_10033-811 TCGAAACGTCCCTTTGTGCTATCGTGACGAACACTGTCTGATGGTCTCTCTGTACGAACTCTGA-------------------------------------------------------------------------------------   319
UHOR_10033-364 TCGAAACGTCCCTTTGTGCTATCGTGACGAACACTGTCTGATGGTCTCTCTGTACGAACTCTGACAGTATAATAGAAGAGGTGAGATGGAAAAGAAGGCGAAAGTGTTGATGACCAGGTTGACGAGGAAGGGAGAGGTGGTGGAGCCAGC   504
UHOR_10033-362 TCGAAACGTCCCTTTGTGCTATCGTGACGAACACTGTCTGATGGTCTCTCTGTACGAACTCTGACAGTATAATAGAAGAGGTGAGATGGAAAAGAAGGCGAAAGTGTTGATGACCAGGTTGACGAGGAAGGGAGAGGTGGTGGAGCCAGC   503
               .......460.......470.......480.......490.......500.......510.......520.......530.......540.......550.......560.......570.......580.......590.......600

UHOR_10033-813 --------------   567
UHOR_10033-822 --------------   571
UHOR_10033-818 --------------   571
UHOR_10033-811 --------------   319
UHOR_10033-364 AGGGAAATTGCATT   518
UHOR_10033-362 AGGGAAATTGCATT   517
               .......610....
```
