# Supplementary material for: An Immunity-Triggering Effector from the Barley Smut Fungus Ustilago hordei Resides in an Ustilaginaceae-Specific Cluster Bearing Signs of Transposable Element-Assisted Evolution
Source: PLoS Pathog. 2014 Jul 3;10(7):e1004223. doi: 10.1371/journal.ppat.1004223 (PMC4081816; doi:10.1371/journal.ppat.1004223)
Supplement: Figure S8 — Identification of specific domains in UhAVR1p and comparison to other Ustilaginaceae effectors. A. Secondary structure prediction using SWISS-MODEL. B. A CLUSTAL 2.1 multiple sequence alignment of UhAVR1p and three effector homologs from U. maydis and Sporisorium reilianum. (PDF) [file ppat.1004223.s008.pdf]

Figure S8

A

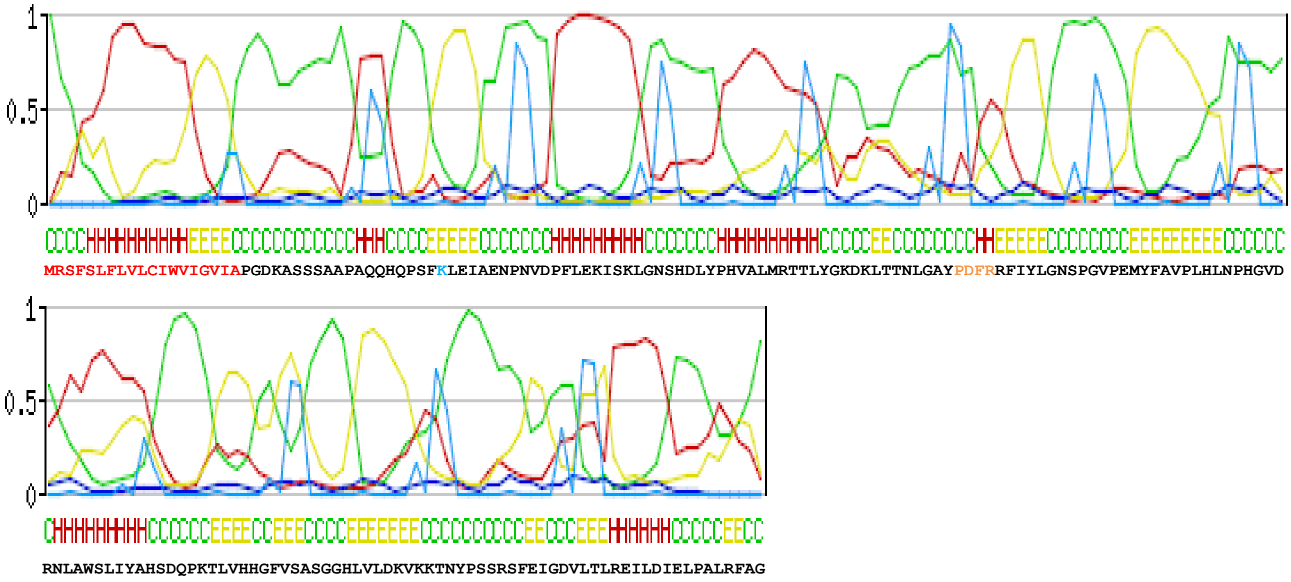

B

```
um10554         IRLDLHLYQSVGLRNYHVSELTSITLNLRELEYQLRYNRSLRQFLHLGPAMGELPGIAAG 111
Sr10052         IRLDLHLYP-LYLRNYRVEEIDKSSLTLRGLENQLRHKRSLRRFLHLGPAEGNRDGFVVG 110
um05295         ARLENHIGDVWQVPNVHVEPVELQSFSPTDLRANFNYDRNLRRHLYLYNTYSNFPDMVFA 120
UhAVR1p         EKIS-KLGNSHDLYP-HVALMRTTLYGKDKLTTNLGAYPDFRRFIYLGNSPG-VPEMYFA 109
                 ::. ::     :   :*  :         *  ::    .:*:.::*  : .    :  .
```

Identification of specific domains in UhAVR1p and comparison to other Ustilaginaceae effectors. Full-length UhAVR1p is 190 aa and has a calculated Mw of 21 kDa and an estimated pI of 8.17 (Protein Calculator v3.3). SignalP 4.1 predicts a 19 aa signal peptide (SP, in red) resulting in a processed protein of 18.9 kDa and an estimated pI=7.75. If 20 aa are cleaved off, then the protein is predicted as myristoylated (prediction by http://mendel.imp.ac.at/myristate/SUPLpredictor.htm). K39 (in blue) has a high probability of being a sumoylation site / SUMO protein attachment site (score 0.85 in SUMOplot Analysis Program, http://www.abgent.com/sumoplot, and 0.967 in http://sumosp.biocuckoo.org/index.php).
A. Secondary structure prediction using SWISS-MODEL http://swissmodel.expasy.org/workspace [100]; C, coil; E, extended beta; H, helix. **B**. A CLUSTAL 2.1 multiple sequence alignment of UhAVR1p and three effector homologs from *U. maydis* and *Sporisorium reilianum* (um05295 , um10554 and Sr10052). The RxLR motifs (highlighted) which have been implicated in membrane PI3P binding and effector uptake in other fungal and oomycete effectors, line up with the PDFR motif in UhAVR1p (orange in A).

100. Arnold K, Bordoli L, Kopp J, Schwede T (2006) The SWISS-MODEL workspace: a web-based environment for protein structure homology modelling. Bioinformatics 22: 195-201
