# Supplementary material for: An Immunity-Triggering Effector from the Barley Smut Fungus Ustilago hordei Resides in an Ustilaginaceae-Specific Cluster Bearing Signs of Transposable Element-Assisted Evolution
Source: PLoS Pathog. 2014 Jul 3;10(7):e1004223. doi: 10.1371/journal.ppat.1004223 (PMC4081816; doi:10.1371/journal.ppat.1004223)
Supplement: Table S1 — U. hordei genes located on BAC3-A2 (117 kb) and their homologs in U. maydis . (PDF) [file ppat.1004223.s011.pdf]

Table S1. *U. hordei* genes located on BAC3-A2 (117 kb) and their homologs in *U. maydis*

Number[1]	*U. hordei* MIPS ID[2]	*U. maydis* homolog[3]	*Um* number[4]	E-Value[5]	Percent Identity[6]	Percent Similarity[7]	score/selfscore ratio query[8]	Function[9]
1	UHOR_08121	um05292	4	0	82.8	94.4	0.85	related to DigA protein
2	UHOR_13886	um10151		2.1E-38	42.9	63.9	0.15	hypothetical protein
3	UHOR_13887	no hit in Um						hypothetical protein
4	UHOR_13888	um04317		0.04	36	52	0.13	hypothetical protein
5	UHOR_08127	um04656		0.02	26.4	45.5	0.06	hypothetical protein *
6	UHOR_08128	um05306	18	1.5E-44	34.2	62.6	0.28	conserved hypothetical Ustilaginaceae-specific protein *
7	UHOR_13890	um00543		0.01	36.4	56.8	0.14	hypothetical protein
8	UHOR_10014	um02565		0	62.4	88.6	0.54	conserved hypothetical protein
9	UHOR_10015	um12288		0	33.5	56.3	0.29	conserved hypothetical protein
10	UHOR_10016	um02285		4.6E-05	20.3	54.2	0.05	hypothetical protein
11	UHOR_13893	um11726		0.01	25	52	0.1	hypothetical protein
12	UHOR_10017	um02745		2.4E-16	30.6	57.5	0.23	conserved hypothetical protein
13	UHOR_10018	um00776		6E-18	46.1	68.5	0.18	conserved hypothetical Ustilaginaceae-specific protein
14	UHOR_10019	um06249		8.8E-05	25.3	53.2	0.11	hypothetical protein
15	UHOR_10020	um02747		4E-12	25.8	60.3	0.14	conserved hypothetical protein
16	UHOR_10021	um05294	6	2.3E-15	27.8	59.3	0.17	conserved hypothetical Ustilaginaceae-specific protein *
		um10554	10	3.0E-14	26.1	61.1	0.16	
		um05295	7	3.8E-12	23.8	57.9	0.15	
		um12302	8	2.6E-11	25	58.7	0.14	
		um10553	9	1.8E-04	22.3	54.2	0.09	

17	UHOR_10022	um12302<br>um05295	8<br>7	1.9E-16<br>2.7E-15	30.5<br>29.5	65.2<br>60.4	0.18<br>0.17	conserved hypothetical Ustilaginaceae-specific protein, UhAvr1 *
18	UHOR_13899	no hit in Um						hypothetical protein
19	UHOR_10023	um04172		0.0007	18.7	56	0.05	probable transposase
20	UHOR_10024	um03280		0.0003	29.4	51	0.09	hypothetical protein
21	UHOR_13901	no hit in Um						*U. hordei*-specific protein (no proper gene call / truncated?)
22	UHOR_10025	um06075		0.008	27	57	0.05	probable transposase
23	UHOR_08123	um05293	5	0	91.6	97.2	0.9	probable oligosaccharyltransferase
24	UHOR_13903	no hit in Um						hypothetical protein
25	UHOR_13904	no hit in Um						hypothetical protein
26	UHOR_10026	um04367		0	58.3	75.4	0.12	related to Gag-pol polyprotein
27	UHOR_10027	um10618		1.1E-11	32.9	71.4	0.12	hypothetical protein
28	UHOR_10028	um02565		0	63	87.3	0.49	conserved hypothetical protein
29	UHOR_13907	no hit in Um						hypothetical protein
30	UHOR_10029	no hit in Um						hypothetical protein
31	UHOR_10030	um05274		0.02	31.2	64.1	0.1	hypothetical protein
32	UHOR_10031	um02565		0	46.1	63	0.12	related to retrotransposon protein
33	UHOR_08130	um05311<br>um05309<br>um05310	23<br>21<br>22	5.9E-21<br>2.4E-10<br>8.1E-10	36.8<br>25.6<br>26.8	67.2<br>60.6<br>64.3	0.21<br>0.13<br>0.13	conserved hypothetical Ustilaginaceae-specific protein *
34	UHOR_08132	um05311<br>um05310<br>um05309	23<br>22<br>21	2.0E-21<br>1.9E-13<br>4.4E-12	33.1<br>30.6<br>26.4	64.5<br>61.8<br>68.0	0.24<br>0.17<br>0.16	conserved hypothetical Ustilaginaceae-specific protein *

35	UHOR_08134	um05312<br>um05317<br>um10557<br>um05318<br>um05314	24<br>29<br>27<br>30<br>26	2.3E-43<br>2.9E-14<br>6.8E-11<br>2.9E-10<br>5.3E-09	45.8<br>25.7<br>28.6<br>26.8<br>25.2	74.8<br>60.3<br>59.7<br>58.5<br>63.1	0.38<br>0.16<br>0.14<br>0.13<br>0.12	conserved hypothetical Ustilaginaceae-specific protein *
36	UHOR_10032	um04367		0	57.9	75.4	0.08	related to pol protein
37	UHOR_13915	no hit in Um						hypothetical protein
38	UHOR_13916	um05318	30	2.0E-08	58.3	83.3	0.28	hypothetical protein *
39	UHOR_10033	um05319	31	4.2E-13	36.7	65.1	0.28	conserved hypothetical Ustilaginaceae-specific protein *
40	UHOR_08135	um10558<br>um05320	32	4.9E-30	93.2	96.8	0.93	probable tubulin beta chain
41	UHOR_08136	um02237		0	26.7	53.9	0.13	conserved hypothetical protein
42	UHOR_08137	um10560	35	0	49.1	73.3	0.4	conserved hypothetical protein
43	UHOR_08138	um10561	36	0	81.6	93.4	0.84	related to VPS10 domain-containing receptor SorCS1 precursor
44	UHOR_08139	um03753<br>um03752		8.7E-19<br>2.2E-14	23.4<br>25.2	55.7<br>52.7	0.13<br>0.11	conserved hypothetical Ustilaginaceae-specific protein *
45	UHOR_13921	no hit in Um						hypothetical protein
46	UHOR_13922	um05325	37	0	64.9	78.4	0.66	conserved hypothetical protein
47	UHOR_13925	um05326	38	0	54.3	72	0.5	conserved hypothetical protein

[1] Number corresponds to predicted genes in the figures
[2] MIPS *U. hordei* strain Uh364 *(MAT-1)* Database gene ID number available at http://www.helmholtz-muenchen.de/en/ibis/institute/groups/fungal-microbial-genomics/resources/muhdb/index.html; color indicates homology / likely family members, and corresponds to Figure 3
[3] Best hits against MIPS *U. maydis* Database: gene ID number (alternate) available at http://www.helmholtz-muenchen.de/en/ibis/institute/groups/fungal-microbial-genomics/resources/mumdb/index.html
[4] arbitrary number given to the corresponding *U. maydis* gene in the Figures

[5] reported expect value based on BLASTp [101]
[6] percent identical amino acids over the length of the matching protein sequences
[7] percent similar amino acids over the length of the matching protein sequences
[8] SIMAP results of the best hit; SIMAP is a program that measures protein similarity based on identities of amino acids in homologous fragments multiplied by the length of the homologous region and divided by the protein length [46]
[9] annotated function of *U. hordei* gene; predicted SSPs indicated with an asterisk

46. Rattei T, Arnold R, Tischler P, Lindner D, Stumpflen V, et al. (2006) SIMAP: the similarity matrix of proteins. Nucleic Acids Res 34: D252-256.
101. Altschul SF, Gish W, Miller W, Myers EW, Lipman DJ (1990) Basic local alignment search tool. J Mol Biol 215: 403-410.
