# Supplementary material for: An Immunity-Triggering Effector from the Barley Smut Fungus Ustilago hordei Resides in an Ustilaginaceae-Specific Cluster Bearing Signs of Transposable Element-Assisted Evolution
Source: PLoS Pathog. 2014 Jul 3;10(7):e1004223. doi: 10.1371/journal.ppat.1004223 (PMC4081816; doi:10.1371/journal.ppat.1004223)
Supplement: Table S2 — Strains used in this work. (PDF) [file ppat.1004223.s012.pdf]

Table S2. Strains used in this work

Strain ID	Relevant Genotype	Comment / Source or origin
Uh359	*MAT-1 Uhavr1*	wild type, alias Uh4854-4, virulent on cv. Hannchen [38]
Uh362	*MAT-2 Uhavr1*	wild type, alias Uh4854-10, virulent on cv. Hannchen [38]
Uh364	*MAT-1 UhAvr1*	wild type, alias Uh4857-4, avirulent on cv. Hannchen [38]
Uh365	*MAT-2 UhAvr1*	wild type, alias Uh 4857-5, avirulent on cv. Hannchen [38]
Um324	*a2b2*	wild type; Um521 [102]
Uh951	Uh364 (*MAT-1*, Δ*UHOR_08134*); hyg^R	this work
Uh1041	Uh364 (*MAT-1*, Δ*18A2*); cbx^R	deletion of fragment 18A2 (see Figure 1); this work
Uh1046	Uh364 (*MAT-1*, Δ*18A3*); cbx^R	deletion of fragment 18A3 (see Figure 1); this work
Uh1051	Uh364 (*MAT-1*, *18A4*); cbx^R	deletion of fragment 18A4 (see Figure 1); this work
Uh1116	*MAT-2*, Δ*18A2* (31); cbx^R	progeny #31 (*MAT-2*) of cross Uh362xUh1041; this work
Uh1117	*MAT-2*, Δ*18A2* (33); cbx^R	progeny #33 (*MAT-2*) of cross Uh362xUh1041; this work
Uh1118	*MAT-2*, Δ*18A2* (35); cbx^R	progeny #35 (*MAT-2*) of cross Uh362xUh1041; this work
Uh1131	Uh364 (*MAT-1*, Δ*18A2-b*); cbx^R	deletion of fragment 18A2-b, clone 52 (see Figure 1); this work
Uh1137	Uh364 (*MAT-1*, Δ*18A2-c*); cbx^R	deletion of fragment 18A2-c, clone 19 (see Figure 1); this work
Uh1142	Uh364 (*MAT-1*, Δ*18A2-c*); cbx^R	deletion of fragment 18A2-c, clone 59 (see Figure 1); this work
Uh1149	Uh364 (*MAT-1*, Δ*18A2-d*); cbx^R	deletion of fragment 18A2-d, clone 1 (see Figure 1); this work
Uh1155	Uh364 (*MAT-1*, Δ*18A2-d*); cbx^R	deletion of fragment 18A2-d, clone 82 (see Figure 1); this work
Uh1166	Uh364 (*MAT-1*, Δ*18A2-a*); cbx^R	deletion of fragment 18A2-a, clone 76 (see Figure 1); this work
Uh1173	Uh364 (*MAT-1*, Δ*18A2-a*); cbx^R	deletion of fragment 18A2-a, clone 316 (see Figure 1); this work
Uh1189	Uh364 (*MAT-1*, Δ*18A2-e*); cbx^R	deletion of fragment 18A2-e, clone 64 (see Figure 1); this work
Uh1197	Uh364 (*MAT-1*, Δ*18A2-e*); cbx^R	deletion of fragment 18A2-e, clone 109 (see Figure 1); this work
Uh1205	Uh1041 [BAC1-6]; cbx^R hyg^R	BAC clone pBAC1-6 (Figure 1A) randomly integrated, clone 2; this work
Uh1207	Uh1041 [BAC1-6]; cbx^R hyg^R	BAC clone pBAC1-6 (Figure 1A) randomly integrated; clone 8; this work
Uh1250	Uh1041 [HSP70:*UHOR_10021:HA*]; cbx^R zeo^R	complemented with randomly integrated effector UHOR_10021:C-terminal HA tag, expressed from constitutive HSP70 promoter, clone 3; this work
Uh1251	Uh1041 [HSP70:*UHOR_10021:HA*]; cbx^R zeo^R	complemented with randomly integrated effector UHOR_10021:C-terminal HA tag, expressed from constitutive HSP70 promoter, clone 4; this work
Uh1253	Uh1041 [HSP70:*UHOR_10021-SP:HA*]; cbx^R zeo^R	complemented with randomly integrated effector UHOR_10021:C-terminal HA tag, minus signal peptide, expressed from constitutive HSP70 promoter, clone 2; this work
Uh1254	Uh1041 [HSP70:*UHOR_10021-SP:HA*]; zeo^R	complemented with randomly integrated effector UHOR_10021:C-terminal HA tag, minus signal peptide, expressed from constitutive HSP70 promoter, clone 3; this work
Uh1255	Uh1041 [HSP70:*UhAvr1:HA*]; cbx^R zeo^R	complemented with randomly integrated effector UHOR_10022:C-terminal HA tag, expressed from constitutive HSP70 promoter, clone 1; this work
Uh1256	Uh1041 [HSP70:*UhAvr1:HA*]; cbx^R zeo^R	complemented with randomly integrated effector UHOR_10022:C-terminal

Strain ID	Relevant Genotype	Comment / Source or origin
		HA tag, expressed from constitutive HSP70 promoter, clone 4; this work
Uh1257	Uh1041 [HSP70:*UhAvr1-SP:HA*]; cbx^{R} zeo^{R}	complemented with randomly integrated effector UHOR_10022:C-terminal HA tag, minus signal peptide, expressed from constitutive HSP70 promoter, clone 4; this work
Uh1258	Uh1041 [HSP70:*UhAvr1-SP:HA*]; cbx^{R} zeo^{R}	complemented with randomly integrated effector UHOR_10022:C-terminal HA tag, minus signal peptide, expressed from constitutive HSP70 promoter, clone 6; this work
Uh1289	Uh364 (*MAT-1*, Δ*UhAvr1*); cbx^{R}	single *UhAvr1* deletion, clone 37; this work
Uh1297	Uh364 (*MAT-1*, Δ*UhAvr1*); cbx^{R}	single *UhAvr1* deletion, clone 106; this work
Uh1351	Uh364 (*MAT-1*, *Avr1* [otef:*gfp*]); zeo^{R}	GFP expressed from strong constitutive *Ustilago* otef promoter; this work
Uh1353	Uh1289 [*UhAvr1:gfp*]; zeo^{R}	Δ*UhAvr1*, replacing deletion by UHOR_10022:GFP chimer, clone 2; this work
Uh1354	Uh1289 [*UhAvr1:gfp*]; zeo^{R}	Δ*UhAvr1*, replacing deletion by UHOR_10022:GFP chimer, clone 3; this work
Uh1355	Uh1289 [*UhAvr1:gfp*]; zeo^{R}	Δ*UhAvr1*, replacing deletion by UHOR_10022:GFP chimer, clone 4; this work
Uh1357	Uh1289 [otef:*UhAvr1:gfp*]; zeo^{R} cbx^{R}	Δ*UhAvr1*, randomly integrated effector UHOR_10022:GFP chimer driven from strong otef promoter, clone 1; this work
Uh1358	Uh1289 [otef:*UhAvr1:gfp*]; zeo^{R} cbx^{R}	Δ*UhAvr1*, randomly integrated effector UHOR_10022:GFP chimer driven from strong otef promoter, clone 2; this work
Uh1359	Uh1289 [otef:*UhAvr1:gfp*]; zeo^{R} cbx^{R}	Δ*UhAvr1*, randomly integrated effector UHOR_10022:GFP chimer driven from strong otef promoter, clone 3; this work
Uh1361	Uh1289 [HSP70:*UhAvr1:HA*]; zeo^{R} cbx^{R}	Δ*UhAvr1*, randomly integrated effector UHOR_10022:C-terminal HA tag chimer, driven from strong HSP70 promoter, clone 1; this work
Uh1362	Uh1289 [HSP70: *UhAvr1:HA*]; zeo^{R} cbx^{R}	Δ*UhAvr1*, randomly integrated effector UHOR_10022:C-terminal HA tag chimer, driven from strong HSP70 promoter, clone 2; this work
Uh1363	Uh1289 [HSP70:*UhAvr1-SP:HA*]; zeo^{R} cbx^{R}	Δ*UhAvr1*, randomly integrated effector UHOR_10022:C-terminal HA tag chimer minus SP, driven from strong HSP70 promoter, clone 1; this work
Uh1369	Uh1289 [HSP70:*UhAvr1*]1; zeo^{R} cbx^{R}	Δ*UhAvr1*, randomly integrated wild type effector UHOR_10022 ORF, driven from strong HSP70 promoter, clone 1; this work
Uh1370	Uh1289 [HSP70: *UhAvr1*]4; zeo^{R} cbx^{R}	Δ*UhAvr1*, randomly integrated wild type effector UHOR_10022 ORF, driven from strong HSP70 promoter, clone 4; this work
Uh1371	Uh1289 [HSP70: *UhAvr1*]9; zeo^{R} cbx^{R}	Δ*UhAvr1*, randomly integrated wild type effector UHOR_10022 ORF, driven from strong HSP70 promoter, clone 9; this work
Uh1372	Uh1289 [complete *UhAvr1* gene]1; zeo^{R} cbx^{R}	Δ*UhAvr1*, randomly integrated complete wild type effector UHOR_10022 gene; transformant 1; this work
Uh1373	Uh1289 [complete *UhAvr1* gene]2; zeo^{R} cbx^{R}	Δ*UhAvr1*, randomly integrated complete wild type effector UHOR_10022 gene; transformant 2; this work

Strain ID	Relevant Genotype	Comment / Source or origin
Uh1374	Uh1289 [complete *UhAvr1* gene]3; zeo^R cbx^R	Δ*UhAvr1*, randomly integrated complete wild type effector UHOR_10022 gene; transformant 3; this work
**World-wide field isolates**		
Uh795	*MAT-1 UhAvr1*	unknown
Uh798	*MAT-2 UhAvr1*	unknown
Uh805	*MAT-1 Uhavr1*	Kenya
Uh811	*MAT-1 Uhavr1*	Ethiopia
Uh813	*MAT-1 UhAvr1*	Iran
Uh815	*MAT-2 Uhavr1*	Canary Island
Uh818	*MAT-1 Uhavr1*	Spain
Uh820	*MAT-2 Uhavr1*	Tunisia
Uh822	*MAT-1 Uhavr1*	Canada
Uh1273	*MAT-1 UhAvr1*	ICARDA Azerbaijan
Uh1278	*MAT-1 Uhavr1*	Hama Hamra, Syria
Uh1283	*MAT-1 UhAvr1*	Turkey
Uh2001-246	*MAT-1 Uhavr1*	Turkey

Uh, *U. hordei*; Um, *U. maydis*. All mutants were generated in the Uh364 background. R, resistant to the indicated antibiotic: hyg, hygromycin B; zeo, zeomycin / zeocin; cbx, carboxin; integrative complementing plasmids are in between square brackets. Δ, deletion mutant, indicating specific gene or region.

38. Linning R, Lin D, Lee N, Abdennadher M, Gaudet D, et al. (2004) Marker-based cloning of the region containing the *UhAvr1* avirulence gene from the basidiomycete barley pathogen *Ustilago hordei*. Genetics 166: 99-111.

102. Kronstad JW, Leong SA (1989) Isolation of two alleles of the b locus of Ustilago maydis. Proc Natl Acad Sci U S A 86: 978-982.
