# Supplementary material for: An Immunity-Triggering Effector from the Barley Smut Fungus Ustilago hordei Resides in an Ustilaginaceae-Specific Cluster Bearing Signs of Transposable Element-Assisted Evolution
Source: PLoS Pathog. 2014 Jul 3;10(7):e1004223. doi: 10.1371/journal.ppat.1004223 (PMC4081816; doi:10.1371/journal.ppat.1004223)
Supplement: Table S3 — Pathogenicity data of U. hordei controls, deletion mutants and complementing transformants. Results from pathogenicity tests of C18A2 deletion mutant complemented with UHOR_10022 with various C-terminal moieties (HA or GFP) on barley cultivars ‘Odessa’ and ‘Hannchen’. (PDF) [file ppat.1004223.s013.pdf]

Table S3. Pathogenicity data of *U. hordei* controls, deletion mutants and complementing transformants.

cross	Strains [1]	barley cv	# plants diseased [2]	plants inoculated	%
1	Uh359 (*MAT-1, Uhavr1*) x Uh362 (*MAT-2, Uhavr1*)	Odessa	6	36	17
1	Uh359 (*MAT-1, Uhavr1*) x Uh362 (*MAT-2, Uhavr1*)	Hannchen	8	40	20
2	Uh362 (*MAT-2, Uhavr1*) x Uh364 (*MAT-1, UhAvr1*)	Odessa	22, 7	54, 32	41, 22
2	Uh362 (*MAT-2, Uhavr1*) x Uh364 (*MAT-1, UhAvr1*)	Hannchen	0, 0	59, 32	0, 0
3	Uh362 (*MAT-2*, *Uhavr1*) x U1041 = Uh364 (*MAT-1*, *Δ18A2*)	Odessa	3	43	7
3	Uh362 (*MAT-2*, *Uhavr1*) x U1041 = Uh364 (*MAT-1*, *Δ18A2*)	Hannchen	12	34	35
4	Uh362 (*MAT-2*, *Uhavr1*) x Uh1255 = Uh1041 (*Δ18A2* [HSP70: *UhAvr1:HA*])1	Odessa	7	79	9
4	Uh362 (*MAT-2*, *Uhavr1*) x Uh1255 = Uh1041 (*Δ18A2* [HSP70: *UhAvr1:HA*])1	Hannchen	14	69	20
5	Uh362 (*MAT-2*, *Uhavr1*) x Uh1256 = Uh1041 (*Δ18A2*, [HSP70: *UhAvr1:HA*])4	Odessa	18	74	24
5	Uh362 (*MAT-2*, *Uhavr1*) x Uh1256 = Uh1041 (*Δ18A2*, [HSP70: *UhAvr1:HA*])4	Hannchen	12	62	19
6	Uh362 (*MAT-2*, *Uhavr1*) x Uh1289 = Uh364 (*MAT-1*, *ΔUhAvr1*)	Odessa	25, 14	40, 33	63, 42
6	Uh362 (*MAT-2*, *Uhavr1*) x Uh1289 = Uh364 (*MAT-1*, *ΔUhAvr1*)	Hannchen	26, 12	56, 33	46, 36
7	Uh362 (*MAT-2*, *Uhavr1*) x Uh1353 = Uh1289 (*MAT-1*, *ΔUhAvr1* [UHOR_10022:GFP])2	Odessa	6	55	11
7	Uh362 (*MAT-2*, *Uhavr1*) x Uh1353 = Uh1289 (*MAT-1*, *ΔUhAvr1* [UHOR_10022:GFP])2	Hannchen	13	56	23
8	Uh362 (*MAT-2*, *Uhavr1*) x Uh1354 = Uh1289 (*MAT-1*, *ΔUhAvr1* [UHOR_10022:GFP])3	Odessa	24	41	59
8	Uh362 (*MAT-2*, *Uhavr1*) x Uh1354 = Uh1289 (*MAT-1*, *ΔUhAvr1* [UHOR_10022:GFP])3	Hannchen	16	57	28
9	Uh362 (*MAT-2*, *Uhavr1*) x Uh1355 = Uh1289 (*MAT-1*, *ΔUhAvr1* [UHOR_10022:GFP])4	Odessa	13	40	33
9	Uh362 (*MAT-2*, *Uhavr1*) x Uh1355 = Uh1289 (*MAT-1*, *ΔUhAvr1* [UHOR_10022:GFP])4	Hannchen	7	37	19
10	Uh362 (*MAT-2*, *Uhavr1*) x Uh1372 = Uh1289 (*MAT-1*, *ΔUhAvr1* [complete *UhAvr1* gene])1	Odessa	6	32	19
10	Uh362 (*MAT-2*, *Uhavr1*) x Uh1372 = Uh1289 (*MAT-1*, *ΔUhAvr1* [complete *UhAvr1* gene])1	Hannchen	0	34	0
11	Uh362 (*MAT-2*, *Uhavr1*) x Uh1373 = Uh1289 (*MAT-1*, *ΔUhAvr1* [complete *UhAvr1* gene])2	Odessa	14	35	40
11	Uh362 (*MAT-2*, *Uhavr1*) x Uh1373 = Uh1289 (*MAT-1*, *ΔUhAvr1* [complete *UhAvr1* gene])2	Hannchen	0	31	0
12	Uh362 (*MAT-2*, *Uhavr1*) x Uh1374 = Uh1289 (*MAT-1*, *ΔUhAvr1* [complete *UhAvr1* gene])3	Odessa	9	34	26
12	Uh362 (*MAT-2*, *Uhavr1*) x Uh1374 = Uh1289 (*MAT-1*, *ΔUhAvr1* [complete *UhAvr1* gene])3	Hannchen	0	32	0
13	Uh362 (*MAT-2*, *Uhavr1*) x Uh1357 = Uh1289 (*MAT-1*, *ΔUhAvr1* [otef:*UhAvr1:GFP*])1	Odessa	9	33	27
13	Uh362 (*MAT-2*, *Uhavr1*) x Uh1357 = Uh1289 (*MAT-1*, *ΔUhAvr1* [otef: *UhAvr1:GFP*])1	Hannchen	10	33	30
14	Uh362 (*MAT-2*, *Uhavr1*) x Uh1358 = Uh1289 (*MAT-1*, *ΔUhAvr1* [otef: *UhAvr1:GFP*])2	Odessa	7	32	22
14	Uh362 (*MAT-2*, *Uhavr1*) x Uh1358 = Uh1289 (*MAT-1*, *ΔUhAvr1* [otef: *UhAvr1:GFP*])2	Hannchen	5	40	13
15	Uh362 (*MAT-2*, *Uhavr1*) x Uh1359 = Uh1289 (*MAT-1*, *ΔUhAvr1* [otef: *UhAvr1:GFP*])3	Odessa	13	33	39
15	Uh362 (*MAT-2*, *Uhavr1*) x Uh1359 = Uh1289 (*MAT-1*, *ΔUhAvr1* [otef: *UhAvr1:GFP*])3	Hannchen	17	39	44
16	Uh362 (*MAT-2*, *Uhavr1*) x Uh1361 = Uh1289 (*MAT-1*, *ΔUhAvr1* [HSP70:*UhAvr1:HA*])1	Odessa	14, 16	31, 32	45, 50
16	Uh362 (*MAT-2*, *Uhavr1*) x Uh1361 = Uh1289 (*MAT-1*, *ΔUhAvr1* [HSP70: *UhAvr1:HA*])1	Hannchen	5, 15	33, 36	15, 42

17	Uh362 (*MAT-2*, *Uhavr1*) x Uh1362 = Uh1289 (*MAT-1*, Δ*UhAvr1* [HSP70:*UhAvr1:HA*])2	Odessa	2, 13	29, 31	7, 42
17	Uh362 (*MAT-2*, *Uhavr1*) x Uh1362 = Uh1289 (*MAT-1*, Δ*UhAvr1* [HSP70: *UhAvr1:HA*])2	Hannchen	1, 5	33, 35	3, 14
18	Uh362 (*MAT-2*, *Uhavr1*) x Uh1363 = Uh1289 (*MAT-1*, Δ*UhAvr1* [HSP70:*UhAvr1-SP:HA*])1	Odessa	12	28	43
18	Uh362 (*MAT-2*, *Uhavr1*) x Uh1363 = Uh1289 (*MAT-1*, Δ*UhAvr1* [HSP70: *UhAvr1-SP:HA*])1	Hannchen	8	31	26
19	Uh362 (*MAT-2*, *Uhavr1*) x Uh1369 = Uh1289 (*MAT-1*, Δ*UhAvr1* [HSP70:*UhAvr1*])1	Odessa	11	33	33
19	Uh362 (*MAT-2*, *Uhavr1*) x Uh1369 = Uh1289 (*MAT-1*, Δ*UhAvr1* [HSP70: *UhAvr1*])1	Hannchen	13	33	39
20	Uh362 (*MAT-2*, *Uhavr1*) x Uh1370 = Uh1289 (*MAT-1*, Δ*UhAvr1* [HSP70: *UhAvr1*])4	Odessa	11	36	31
20	Uh362 (*MAT-2*, *Uhavr1*) x Uh1370 = Uh1289 (*MAT-1*, Δ*UhAvr1* [HSP70: *UhAvr1*])4	Hannchen	12	35	34
21	Uh362 (*MAT-2*, *Uhavr1*) x Uh1371 = Uh1289 (*MAT-1*, Δ*UhAvr1* [HSP70: *UhAvr1*])9	Odessa	7	30	23
21	Uh362 (*MAT-2*, *Uhavr1*) x Uh1371 = Uh1289 (*MAT-1*, Δ*UhAvr1* [HSP70: *UhAvr1*])9	Hannchen	12	31	33

[1] For descriptions of strains and deletion mutants, see Table S2. Complementing plasmid constructs are given between square brackets, expressing the corresponding gene chimer (either linked to GFP or the HA epitope tag) from either the *otef* or *U. maydis* HSP70 promoter. –SP indicates the effector gene is lacking the predicted signal peptide sequence.

[2] Pathogenicity tests are variable; ratings for the same cross should be compared with respect to infection on universal suscept 'Odessa'
