# Supplementary material for: An Immunity-Triggering Effector from the Barley Smut Fungus Ustilago hordei Resides in an Ustilaginaceae-Specific Cluster Bearing Signs of Transposable Element-Assisted Evolution
Source: PLoS Pathog. 2014 Jul 3;10(7):e1004223. doi: 10.1371/journal.ppat.1004223 (PMC4081816; doi:10.1371/journal.ppat.1004223)
Supplement: Table S4 — Primers used in this work. (PDF) [file ppat.1004223.s014.pdf]

Table S4. Primers used in this work

#	Name of the primer	Sequence	Purpose
661	Uh5311R2	GCATTCGGCCTGATACCAAC	Sequencing of UHOR_08132
727	Uh05318homo_r	GCGCACTAGTTCAATTCAAAGCTGGAGGTATG	Sequencing of UHOR_10033
728	Uh05294plus_f	GGAACTGTGCTCTGGTAGTGG	Sequencing of UHOR_10021
730	Uh05306plus_f	ATAGCCCTCCTTCATGGTGT	Sequencing of UHOR_08128
731	Uh05306plus_r	TCGCAGTGCCTTTCATATTG	Sequencing of UHOR_08128
736	Uh05311aPlus_f	GCTTTTCATCAGAGCCATACCT	Sequencing of UHOR_08130
737	Uh05311aPlus_r	TGGCTTGTTTACAGAGTGCAA	Sequencing of UHOR_08130
966	5319 plus_r	TCTCCCTTCCTCGTCAACCTG	Sequencing of UHOR_10033
1012	*UHOR_08134* nested_f	TGAAGGCGCGCCATGTCAGAGGAAGCGAG	To generate deletion construct
1035	*UHOR_08134* 5′-flank_f	GGTACGCCGATCACTTCAAC	5F of deletion construct
1038	Hyg- *UHOR_08134* 5′-flank	ATAATCCTTAAAAACTCCATTTCCACCCCTGTAGACAGATGTACCTTCAT	5F of deletion construct
1221	Hyg- *UHOR_08134* 3′-flank	ACTTTATTGTCATAGTTTAGATCTATTTTGACTCCTGTGACCATTCGAGG	3F of deletion construct
1222	*UHOR_08134* 3′-flank_r	CCGATACCGAGACAATAGCTG	3F of deletion construct
1223	*UHOR_08134* nested_r	GTGCATGGTGTTGGAGGTC	To generate deletion construct
1152	UH_08134_Fw	CACCATGAAGGTACATCTGTCTAC	Sequencing of UHOR_08134
1154	UH_08134_Rev	GCCTCGAATGGTCACAGG	Sequencing of UHOR_08134
1244	UH_13897_Fw	**CACC**ATGCTTACTCAACCGGCCAAC	Cloning of UHOR_10021
1245	UH_13897-SP_Fw	**CACC**ATGGCACTACCCGGTCGCAGCTAC	Cloning of UHOR_10021
1246	UH_13897_Rev	CATTCGTGACAACGTCTCAAAAAC	Cloning and sequencing of UHOR_10021
1247	UH_10022_Fw	**CACC**ATGCGATCGTTTTCCCTTTTCC	Cloning and sequencing of UHOR_10022
1248	UH_10022-SP_Fw	**CACC**ATGCCTGGCGACAAAGCTTCTTC	Cloning of UHOR_10022
1249	UH_10022_Rev	TCCGGCAAATCGGAGCGCAG	Cloning and sequencing of UHOR_10022
1253	UH_08132_Fw	CACCATGGCCACAACATCACTCTTAC	Sequencing of Uh08132
1256	UH_08128_Fw	CACCATGCTTTTCTTTATTCTCGCC	Sequencing of UHOR_08128
1258	UH_08128_Rev	AGAAAAGTGCGGCAGTGATGC	Sequencing of UHOR_08128
1261	UH_08127_Rev	TCCGTGGCCTCTAACAGCAAG	Sequencing of UHOR_08127
1265	UH_08139_Fw	CACCATGTTCCGAATCGGCTTTGTC	Sequencing of UHOR_08139

#	Name of the primer	Sequence	Purpose
1267	UH_08139_Rev	TCCAGGCAATCTGATCAGGC	Sequencing of UHOR_08139
1272	Uh08127-plusL	GCAGAGCATGACGTGAAACTAC	Sequencing of UHOR_08127
1273	Uh08127-plusR	GTTCAAGGCCCTATATCCCTCT	Sequencing of UHOR_08127
1277	Uh10024-plusL	TGAGATCTGTCATAGAGCTGTTTC	Sequencing of UHOR_10024
1278	Uh10024-plusR	GCATCTTCGGATGTCAGGTAGT	Sequencing of UHOR_10024
1281	Uh08132-plus-L	CGCTATGGAAGCACTTCATTTT	Sequencing of UHOR_08132
1282	Uh10033-plus-L	GGTCAAGTCGACCTCCAACAG	Sequencing of UHOR_13916
1283	Uh10033-plus-R	GTCCCTTCCGTCACTTCCAT	Sequencing of UHOR_13916
1284	C19-A1-5L-1Sce-1F	**AAAATAGGGATAACAGGGTAAT**CGACAGATCTCGAGGAAACC	5F of deletion construct C18A2
1285	C19-A1-5R-flank–attB1	**GGGGACAAGTTTGTACAAAAAAGCAGGCTAT**TGAATTGTTTGCCACACCTG	5F deletion construct C18A2
1286	C19-A1-ko-5 FlankL	TCACTTCAGGAGGTGATCAAGA	Confirmation of deletion mutant C18A2
1289	C19-A2-3L- attB2	**GGGGACCACTTTGTACAAGAAAGCTGGGTA**GGAGAGAAGAAGCAGAGCT	3F of deletion construct C18A2
1290	C19-A2-3R-1Sce-1R	**AAAAATTACCCTGTTATCCCTA**TTGTGCTTCACTGCACCTTC	3F of deletion construct C18A2
1291	C19-A2-ko-3 FlankR	TCCCTGTCGGTGTCTTCTTACT	Confirmation of deletion mutant C18A2
1292	C19-A3-5L-1sce-1F	**AAAATAGGGATAACAGGGTAAT**CCTGTCGATTGCTAGGAAGG	5F deletion construct C18A3
1293	C19-A3-5R–attB1	**GGGGACAAGTTTGTACAAAAAAGCAGGCTAT**TGAGGGTCAATCGGAGAGAT	5F deletion construct C18A3
1294	C19-A3-ko-5 FlankL	TTGTTGTCTTGGTTTCCTGTGT	Confirmation of deletion mutant C18A2-A
1295	C19-A4-5L-1Sce-1F	**AAAATAGGGATAACAGGGTAAT**AAGCCCTGCTTCTTCTCTCC	3F of deletion construct C18A3
1296	C19-A4-5R-attB1	**GGGGACAAGTTTGTACAAAAAAGCAGGCTAT**GAGTGGATCCCCATTGTCAT	3F of deletion construct C18A3
1297	C19-A4-ko-5 FlankL	AGCTTGCAGTCTGTTCATCATC	Confirmation of deletion mutant C18A5
1298	C19-A4-3L attB2	**GGGGACCACTTTGTACAAGAAAGCTGGGTA**CGTACAGGACCGTGAGGACT	5F deletion construct C18A4
1299	C19-A4-3R-1Sce-1-R	**AAAAATTACCCTGTTATCCCTA**GTGGATCAGCTGTTCACTCG	5F deletion construct C18A4
1428	C19A2-A-3F-attB2-L	**GGGGACCACTTTGTACAAGAAAGCTGGGTA**GCATTGTGCTCAAGCTGTGT	3F deletion construct C18A-A
1429	C19A2-A-3F-1sce1R-R	**AAAAATTACCCTGTTATCCCTA**ACTGCTGGGCAAGAATGACT	3F deletion construct C18A-A
1430	C19A2-b-5F-1sce1F-L	**AAAATAGGGATAACAGGGTAAT**CTCAAACCCAATCTGCAGTG	5F deletion construct C18A-B
1431	C19A2-b-5F-attB1-R	**GGGGACAAGTTTGTACAAAAAAGCAGGCTAT**AGGTTAGCGGTCCAGATCAA	5F deletion construct C18A-B
1432	C19A2-b-3F-attB2-L	**GGGGACCACTTTGTACAAGAAAGCTGGGTA**CTAGGACGAAACAGCCAAGC	3F deletion construct C18A-B
1433	C19A2-b-3F-1SceR-R	**AAAAATTACCCTGTTATCCCTA**ACTCCAATCACGGGAATCAC	3F deletion construct C18A-B
1434	C19A2-c-5F-1sce1F-L	**AAAATAGGGATAACAGGGTAAT**TGGGTAGAGGTTTGGTGAGG	5F deletion construct C18A-C
1435	C19A2-c-5F-attB1-R	**GGGGACAAGTTTGTACAAAAAAGCAGGCTAT**AAGAATCCTGCCTTGCTTCA	5F deletion construct C18A-C

#	Name of the primer	Sequence	Purpose
1436	C19A2-c-3F-attB2-L	**GGGGACCACTTTGTACAAGAAAGCTGGGTA**CCTTAGCCTAGTCCCGCTCT	3F deletion construct C18A-C
1437	C19A2-c-3F-1SceR-R	**AAAAATTACCCTGTTATCCCTA**GAGAAGAAGCAGGGCTTTCA	3F deletion construct C18A-C
1438	C19A2-d-5F-1sce1F-L	**AAAATAGGGATAACAGGGTAAT**TTCATCTTCGCCCATTCTTC	5F deletion construct C18A-D
1439	C19A2-d-5F-attB1-R	**GGGGACAAGTTTGTACAAAAAAGCAGGCTAT**TTGAAGCTCCTCGTCAGACA	5F deletion construct C18A-D
1440	C19A2-d-3F-attB2-L	**GGGGACCACTTTGTACAAGAAAGCTGGGTA**CATCATCATAGGCTGAGTGGA	3F deletion construct C18A-D
1441	C19A2-d-3F-1SceR-R	**AAAAATTACCCTGTTATCCCTA**GGCAAGCTTTGACTTGGAAT	3F deletion construct C18A-D
1446	C19A2-e-3F-attB2-L	**GGGGACCACTTTGTACAAGAAAGCTGGGTA**GAGACGATCGTGCGTATGTG	3F deletion construct C18A-E
1447	C19A2-e-3F-1SceR-R	**AAAAATTACCCTGTTATCCCTA**TTCACTGCGATCTGCCATAG	3F deletion construct C18A-E
1506	C19A2-A-KO-3F	TTACAATTGCAGGCAACCAG	Confirmation of deletion mutant C18A2-A
1507	C19A2-B-KO-5F	GCATATGGCTTCTTGCCATT	Confirmation of deletion mutant C18A2-B
1508	C19A2-D-KO-3F	TGTCATACAGCCCCAGATCA	Confirmation of deletion mutant C18A2-D
1511	Uh13899-L	CGTTTGACAAGGACGACAGA	For PCR amplification
1513	VirC17R1	CTGCAGGTCGACTCTAGAGG	For PCR of transposable element
1551	C19A2-E-ko-3F	TGATGCTCATGCTGATTTCA	Confirmation of deletion mutant C18A2-D
1614	Uh10022-5F-attBI	**GGGGACAAGTTTGTACAAAAAAGCAGGCTAT**AGGTTAGTGGTCAGTTTATC	5F deletion construct UHOR_10022
1615	Uh10022F	CACCGTGCACCATGGATTCGTCT	for RNAi
1668	10026F	TGTCGGTTGTTGGTCTTCCC	For PCR amplification
1669	10027R	TGATCAACCACATGGGTGCT	For PCR amplification
1670	10028F	CCAGTAGCCTGGAAGTCAGC	For PCR amplification
1671	10028R	TAGACTTTCGTGCGTTGTGC	For PCR amplification
1672	13901F	GAATTTCCGAGTCGATCCAA	For PCR amplification
1673	10030R	GCAAGAGGGAGCAACAAGTC	For PCR amplification
1685	10021F1	CGATGTAGCGGGTCTCGAAG	For qPCR and PCR amplification
1689	10022 qPCR-L	GGTGGACACCTGGTCCTAGA	Flanking primer for UhAvr1
1798	Avr1249NEST	CAGGCAGTTCAATATCAAG	Nested primer for UhAvr1
1799	Avr1689NEST	AGATAAAGTTAAAAAAACT	Nested primer for UhAvr1
1804	UH07772-5PRIME-B	AACTTCTGCGCGACACAC	Flanking primer for eIF-B2
1805	UH07772-3PRIME-B	ATACCTGATGACCTCTTCTG	Flanking primer for eIF-B2
1811	Avr1804NEST	CACGCAAAGTACTTTAAG	Nested primer for eIF-B2
1812	Avr1805NEST	CTGCCGCCTTCCTCAACTGC	Nested primer for eIF-B2

#	Name of the primer	Sequence	Purpose
1815	10022SP-ATG-rev	GGAAAAGGGAAAACGATCGCAT	For (inverse) PCR amplification
1816	10022SP-5start-fw	CTCTGCATATGGGTCATCGGCG	For inverse PCR amplification
1890	Uh362C17R2	GCCTCCCAATGGGTTTCG	For PCR of transposable element
1891	Uh10026-L58790	AATGGGACTACAGAGTACAAGG	For PCR of transposable element
1904	UhAvr1gene_fw	CGTGGGATCCTCAGACTGAACACCGGTGCACTGC	to clone complete wt *UhAvr1* gene in pUBle3 – BamHI sites
1905	UhAvr1gene-rev	CGTCGGATCCATGGTCAAGATCCTTGCGCAGCTCG	to clone complete wt *UhAvr1* gene in pUBle3 – BamHI sites

#, primer inventory number. F, forward; R, reverse. 3F and 5F indicates primers were used for the amplification of 3'- and 5'-ends of deleted regions. The I-SceI recognition sequence is in bold type and underlined, while only bold type represents the *attB1* and *attB2* sequences on the primers used for the deletion constructs. The tetranucleotide CACC in bold type indicates the sequence used for directional cloning in the pENTR/D^{TM} Gateway plasmid (Invitrogen).
