# Supplementary material for: An Immunity-Triggering Effector from the Barley Smut Fungus Ustilago hordei Resides in an Ustilaginaceae-Specific Cluster Bearing Signs of Transposable Element-Assisted Evolution
Source: PLoS Pathog. 2014 Jul 3;10(7):e1004223. doi: 10.1371/journal.ppat.1004223 (PMC4081816; doi:10.1371/journal.ppat.1004223)
Supplement: Table S5 — Annotated genes in the region of the U. maydis 19A cluster. (PDF) [file ppat.1004223.s015.pdf]

Table S5. Annotated genes in the region of the *U. maydis* 19A cluster.

Number [1]	*U. maydis* MIPS ID [2]	*U. maydis*, alternate MIPS ID [2]	Coordinates on chrom 19		Function [3]
1	um10705	um05289	131692	132810	cyclin-dependent kinase 1
2	um05290		134828	133131	putative protein
3	um05291	umd191-470	136367	135366	conserved hypothetical protein
4	um05292		137169	141062	related to DigA protein
5	um05293		142124	144523	probable oligosaccharyltransferase
6	um05294		145921	146535	conserved hypothetical Ustilago-specific protein *
7	um05295		147006	147635	conserved hypothetical Ustilago-specific protein *
8	um12302	um05296	148362	148916	conserved hypothetical Ustilago-specific protein *
9	um10553	um05297	150051	150695	conserved hypothetical Ustilago-specific protein *
10	um10554	um05298	151334	151915	conserved hypothetical Ustilago-specific protein *
11	um05299		153386	154090	conserved hypothetical Ustilago-specific protein *
12	um05300		154615	155271	conserved hypothetical Ustilago-specific protein *
13	um05301		156973	157728	conserved hypothetical Ustilago-specific protein *
14	um05302		159396	160019	hypothetical protein *
15	um05303		160415	160996	hypothetical protein *
16	um10555	um05304	161948	162550	conserved hypothetical protein *
17	um05305		164040	164723	conserved hypothetical Ustilago-specific protein *
18	um05306		165279	166142	conserved hypothetical Ustilago-specific protein *
19	um10556	um05307	167168	166284	hypothetical protein *
20	um05308		169924	170652	hypothetical protein *
21	um05309		173231	173818	conserved hypothetical Ustilago-specific protein *

22	um05310		174557	175144	conserved hypothetical Ustilago-specific protein *
23	um05311		176039	176701	conserved hypothetical Ustilago-specific protein *
24	um05312		177055	177603	conserved hypothetical Ustilago-specific protein *
25	um05313		178662	180186	hypothetical protein
26	um05314		181718	182314	conserved hypothetical Ustilago-specific protein *
27	um10557	um05315	183874	184416	conserved hypothetical Ustilago-specific protein *
28	um05316	umd191-730	184994	185813	related to transposase, pseudogene
29	um05317		186566	187102	conserved hypothetical Ustilago-specific protein *
30	um05318		187537	188088	hypothetical protein *
31	um05319		188591	189148	conserved hypothetical Ustilago-specific protein *
32	um10558	um05320	189722	191330	probable tubulin beta chain
33	um10559	um05321	194157	191683	conserved hypothetical protein
34	um05322		196067	195681	hypothetical protein
35	um10560	um05323	196939	198717	conserved hypothetical protein
36	um10561	um05324	200035	204555	related to VPS10 domain-containing receptor SorCS1 precursor
37	um05325		207057	209936	conserved hypothetical protein
38	um05326		211663	217938	conserved hypothetical protein

[1] Number corresponds to predicted *U. maydis* genes in Figure 4
[2] MIPS *U. maydis* Database gene ID number available at http://mips.helmholtz-muenchen.de/genre/proj/ustilago/; color indicates homology / likely family members, and corresponds to Figure 4
[3] annotated function of *U. maydis* gene; the 24 predicted SSPs are indicated with an asterisk [26]
